# Iron *N*‑Heterocyclic Carbene Photoactive Complexes with Rigid Phenylethynyl Substituents as Ligand π‑System Extensions

**DOI:** 10.1021/acs.inorgchem.5c01461

**Published:** 2025-06-09

**Authors:** Samuel Persson, Raj Kumar Koninti, Mariam Barakat, Abhishek Mishra, Fredrik Lindgren, Tore Ericsson, Lennart Häggström, Sven Lidin, Ana Gonzalez, Elena Jakubikova, Reiner Lomoth, Kenneth Wärnmark

**Affiliations:** † Centre for Analysis and Synthesis, Department of Chemistry, 530031 Lund University, Box 124, SE-22100 Lund, Sweden; ‡ Department of Chemistry − Ångstrom Laboratory, Uppsala University, Box 523, SE- 75120 Uppsala, Sweden; § 6798 North Carolina State University, Department of Chemistry, Raleigh, North Carolina 27695, United States; ∥ Department of Physics − Ångström Laboratory, Uppsala University, Box 530, SE-751 21 Uppsala, Sweden; ⊥ BioMAX, MAX IV, Lund University, Box 188, SE-221 00 Lund, Sweden

## Abstract

The design of iron complexes with long-lived charge transfer states suitable for applications as photosensitizers remains a formidable challenge. Here, we investigated the effect of an extended ligand π-system on the ground- and excited-state properties of iron­(II) complexes with *N*-heterocyclic carbene (NHC) ligands. For this purpose, three iron complexes based on the established [Fe­(II)­(pbmi)_2_]^2+^ motif (pbmi = (1,1′-(pyridine-2,6-diyl)­bis­(3-methylimidazole-2-ylidene))) have been modified with phenylethynyl moieties attached to the pyridine part of the ligand. In general, the introduction of the phenylethynyl units served to red shift the main absorption band, as well as to increase the extinction coefficient of the same, compared to the parent complex. The lowered MLCT energies are in line with the electrochemical data that revealed substantially easier reduction of the phenylethynyl-modified ligands, while the potentials of the Fe­(III/II) couple are only moderately increased. Only minor modifications of the electronic effect intrinsic to the phenylethynyl moieties could be implemented with bromide and dimethylamino substituents on the phenylene units. As a result, all three complexes experience similar stabilization of their ^3^MLCT states, about 0.3 eV compared to the parent complex, and feature transient absorption data in line with ES dynamics that are dominated by a moderately long-lived (∼17 ps) ^3^MLCT state. These values exceed the ^3^MLCT lifetimes reported for the parent complex (up to 9 ps) and resemble the results for carboxylic acid and imidazolinium derivatives with comparable ^3^MLCT energies and lifetimes.

## Graphical abstract

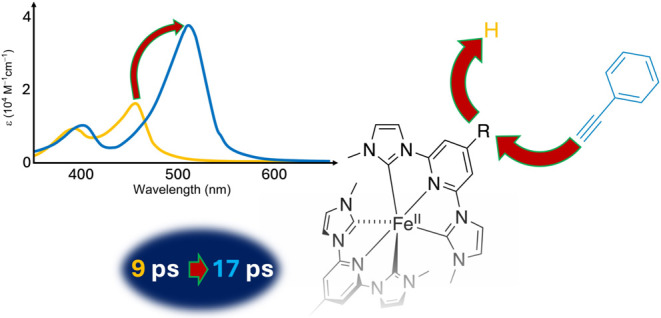

## Introduction

The use of metal-based photosensitizers has long focused on the use of transition-metal complexes that exhibit long-lived metal-to-ligand charge transfer (MLCT) states. These complexes have in the past mostly been based on scarce metals, such as Ru and Ir, as these metals yield complexes with large ligand field splitting, conducive to achieving long-lived MLCT states.^1^ The ligands in these metal complexes have traditionally been based on polypyridyl ligands,^2^ some having cyclometallating groups as a part of the framework.^3^ The use of rare metals does however have certain drawbacks: large-scale application is held back both by the cost of these materials as well as their fundamentally limited amount available on the planet.^4,5^

There has for this reason been a push toward establishing photosensitizers of various earth-abundant metals, such as Co, W, Mo, Cr, Mn, Ni, Zn, V, Ti, and Cu.^6–11^ Iron has a special place in this research due to it sharing a group in the periodic table with ruthenium, a metal well-established for photosensitizer complexes.^11,12^ Iron is also the most abundant transition metal on Earth, making it a prime candidate for investigations into large-scale applications. However, despite its similarities to ruthenium, the MLCT state in iron polypyridyl systems has a lifetime on a subpicosecond timespan, as opposed to the nanosecond–microsecond lifetimes found in analogous ruthenium complexes.^13^ The smaller polarizability of electron densities of iron gives efficient deactivation pathways for the charge transfer states to decay into ligand field states.^14^ The use of strongly σ-donating *N*-heterocyclic carbene (NHC) ligands has been explored to counteract these problems with iron. The strongly σ-donating character of NHCs works to somewhat overcome the issues of iron’s poor polarizability.^15–17^ The use of hexacarbene structures has furthermore been successful in establishing Fe­(II) complexes with MLCT states with lifetimes of 0.5 ns,^18^ as well as Fe­(III) complexes with ligand-to-metal charge transfer (LMCT) states with lifetimes in the 0.1–2 ns time range.^19,20^ However, by having all coordination sites at the iron occupied by NHC units, tuning of the properties of the metal becomes more challenging. This is due to less direct communication between substituents and the metal being possible across the carbene units (due to their lower π-accepting ability) compared to other motives such as those including pyridine groups bound to iron. Furthermore, the six-carbene coordination environment makes the Fe­(II) state susceptible to oxidation to Fe­(III).^20^ The use of fewer carbenes has however not yielded equally impressive results, with regard to increasing the charge transfer state lifetimes of iron complexes; however, the incorporation of cyclometallating moieties in a polypyridine ligand framework has led to a 1 ns lifetime of its ^3^MLCT state.^21^ This improvement comes, however, at the price of a severely lowered excited-state energy that limits the range of potential applications as a photosensitizer.

An alternative strategy to increase the lifetime of the MLCT state is by extending of the ligand π-system, which stabilizes the energies of the unoccupied ligand π* orbitals.^22^ This strategy has successfully been incorporated by fusing a phenylene moiety to the 3-methylimidazole-2-ylidene moieties of the first-generation iron-NHC complex for photophysical applications, [Fe­(pbmi)_2_]­(PF_6_)_2_ (complex **1** in Figure 1; pbmi = 1,1′-(pyridine-2,6-diyl) bis­(3-methylimidazole-2-ylidene)), extending the lifetime from up to 9 ps of the ^3^MLCT state,^15^ to 26 ps for the resulting analogous iron-NHC complex having a ligand based on the 3-methylbenzimidazole-2-ylidene moiety.^23^ Exchanging the central pyridine moiety for a π-electron-deficient 1,4-diazine moiety lowered the energy of the ^3^MLCT state, leading to an increase of the excited-state lifetime to 36 ps of a ^3^MLCT/equilibrated ^3^MC state.^24^

**Figure 1:**
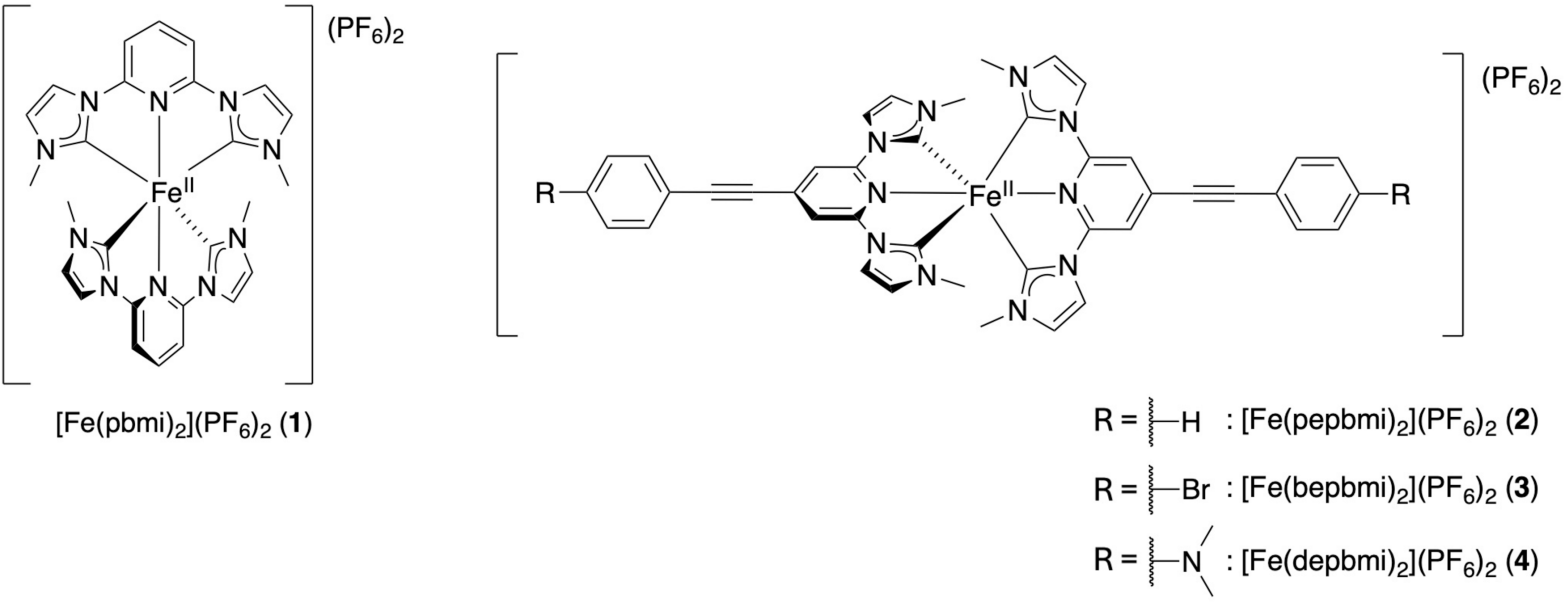
Structure of the complexes investigated herein.

Another strategy to increase the π-system can be achieved by introducing ethynyl substituents into the core ligand. This approach has been used previously in iron-NHC complexes.^25–28^ While this includes bistridentate Fe­(II) complexes with arylethynyl moieties, the effect of these substituents on redox properties and excited-state dynamics as compared to complexes with unsubstituted ligand motifs has not been addressed in previous reports. In this study, we investigated the effect of phenylethynyl moieties on the ground- and excited-state properties of bistridentate Fe­(II) complexes with methylimidazole-based NHC ligands. Furthermore, for parent complex **1**,^15^ recent studies indicate that its excited-state dynamics is highly sensitive to the effect of electron-withdrawing substituents on the energy of the ^3^MLCT state,^29,30^ showing that substituents with extended π-system/electron-withdrawing groups favor excited-state dynamics in which the deactivation of the ^3^MLCT state goes exclusively via the ^3^MC state, with a robust lifetime of around 20 ps for the former state.

For the purpose above, we have prepared a series of complexes with phenylethynyl moieties in the 4-position of each pyridine moiety of the NHC containing ligand (Figure 1). These moieties were decorated with different functional groups at the terminal position of phenylenes to modulate their electron-withdrawing effect.

Our results demonstrate that modification of the pbmi ligands with phenylethynyl moieties results in increased extinction coefficients and lowered energies of the MLCT absorption bands. Stabilization of the ^3^MLCT state apparently disfavors deactivation via metal-centered (MC) states and enables ^3^MLCT lifetimes on par with values previously reported for imidazolium and carboxylic acid derivatives of the pbmi ligand motif.^30,31^

## Results and Discussion

### Synthesis

Complexes [Fe­(pepbmi)_2_]­(PF_6_)_2_ (**2**), [Fe­(bepbmi)_2_]­(PF_6_)_2_ (**3**), and [Fe­(depbmi)_2_]­(PF_6_)_2_ (**4**) (Figure 1) (pepbmi = 1,1′-(((phenyl)­ethynyl)­pyridine-2,6-diyl) bis­(3-methylimidazole-2-ylidene); bepbmi = 1,1′-(((4-bromophenyl)­ethynyl)­pyridine-2,6-diyl) bis­(3-methylimidazole-2-ylidene); and dpbmi = 1,1′-(((4-(*N*,*N*-dimethylamino)­phenyl)­ethynyl)­pyridine-2,6-diyl) bis­(3-methylimidazole-2-ylidene)) (Figure 1) were prepared as described below.

#### Ligand Synthesis

Syntheses of the three ligands were carried out according to a shared principle with certain modifications implemented depending on the ligand. The first step in each synthetic pathway is the introduction of two imidazolyl units to the core pyridine via a S_N_Ar reaction with 2,6-fluoro-4-iodopyridine **5**. This reaction proceeded cleanly and in good yield of 93%, with minimal purification needed to obtain compound **6** (Scheme 1).

**Scheme 1:**
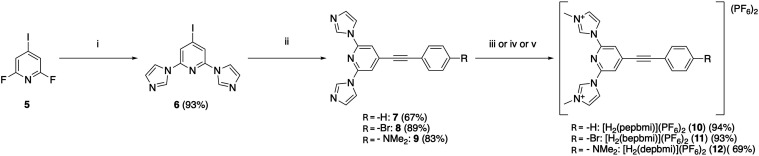
Synthetic Procedures Used to Obtain Precarbene Ligands (**10**–**12**) Used to Achieve New Complexes in This Work^a^

a (i) Sodium imidazolate, DMF, 50 °C. (ii) Phenylacetylene/1-bromo-4-ethynylbenzene/4-ethynyl-*N*,*N*-dimethylaniline, Pd­(PPh_3_)_2_Cl_2_, Cu, DMF, DIPA, rt. (iii) MeI, DMF, 130 °C (used for **10**). (iv) MeOTf, DCM, rt (used for **11**). (v) MeI, MeCN, 85 °C (used for **12**).

From this intermediate, each phenylethynyl moiety was introduced via a Sonogashira cross-coupling reaction with the appropriate ethynylbenzene derivative. These reactions also proceed with relatively good yields of 67–89%, without much difficulty, to give intermediates **7**–**9** (Scheme 1).

Finally, the precarbene methyl-imidazolium moieties were created by treating the intermediate with a methylating agent (MeOTf or MeI). Somewhat different conditions were needed for each derivative. Especially, the dimethylamino substituent was troublesome, yielding impurities not found in the other cases and requiring flash chromatography to access the pure material. The other two derivatives (**10** and **11**) were pure after simple precipitation from the reaction mixture. The dimethylamino derivative **12** thus also gave a lower yield of 69%, as opposed to the other two derivatives **10** and **11**, which are methylated with yields above 90% (Scheme 1). See the Supporting Information for full synthetic procedures (NMR spectra of new intermediates in Figures S1–S28).

#### Syntheses of Iron Complexes

Each of the three complexes were synthesized according to the same general principle as other iron-NHC complexes of a similar core structure.^15,16^ This consisted of first deprotonating the NHC moieties of the ligand with a strong base, creating free carbene. After allowing the carbene time to form, iron was introduced in the form of iron­(II) bromide, leading to formation of the desired homoleptic complex (Scheme 2).

**Scheme 2:**
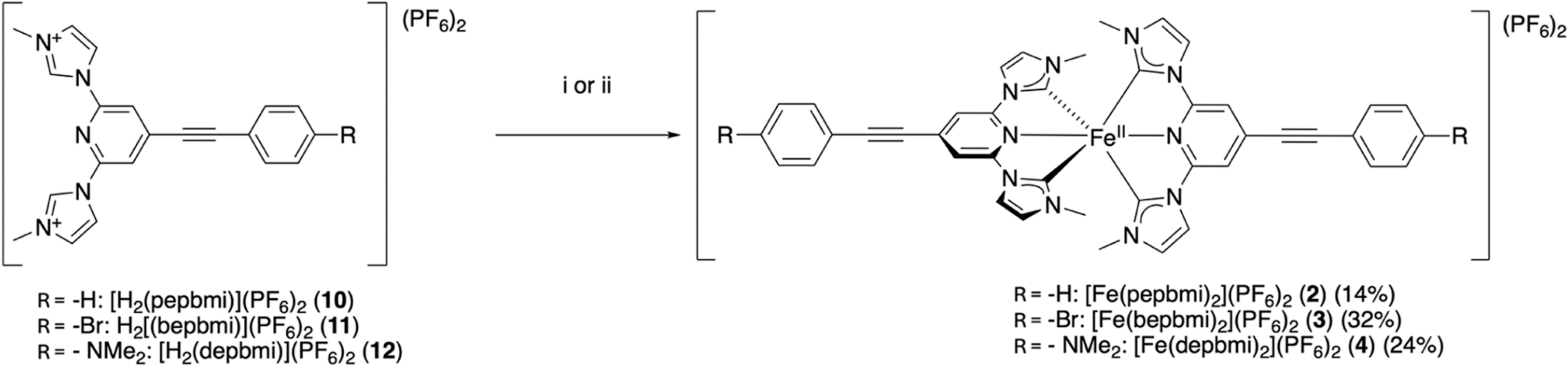
Synthetic Procedures Used to Obtain Various Complexes (**2**–**4**) Investigated in This Study^a^

a (i) (1) KO*t*-Bu, THF, −78 °C; (2) FeBr_2_, THF, rt (used for **2** and **4**). (ii) (1) LDA, THF, −40 °C; (2) FeBr_2_, THF, rt (used for **3**).

In the case of these complexes, it was found that depending on the ligand used, different bases gave different yields. For the bromo-substituted ligand **11**, the use of LDA as a base generated the complex [Fe­(bepbmi)_2_]­(PF_6_)_2_ (**3**) in a yield of 32%. The use of the more commonly applied KO*t*-Bu as a base gave only minor amounts of the complex. On the contrary, use of LDA with the dimethylamino ligand **12** failed to give any of the desired complex [Fe­(depbmi)_2_]­(PF_6_)_2_ (**4**), and use of KO*t*-Bu gave the complex in 24%. The unsubstituted ligand gave similar results with either base, with KO*t*-Bu working slightly better and giving a yield of 14% for the formation of the complex [Fe­(pepbmi)_2_]­(PF_6_)_2_ (**2**).

For all complexes, it was found that size-exclusion chromatography was the most efficient way of purification. This chromatography removed an unidentifiable impurity that was present in all cases and seemed to be of a similar nature in each case. A small amount of unreacted ligand was also present from each reaction, and this was also removed by size-exclusion chromatography. See the Supporting Information for full synthetic procedures (NMR spectra of new complexes in Figures S29–S40).

### XRD Structure Determination

Single crystals of **2** and **4** suitable for XRD were obtained by slow diffusion of Et_2_O into a solution of the complex in a 1:1 mixture of MeCN and toluene. Single crystals of **3** suitable for XRD were obtained by dissolving the complex in a 1:1 mixture of MeCN and toluene and slowly letting the solvent evaporate, leaving only toluene. A full refinement of the molecular structure of **4** could not be obtained due to symmetry-breaking features in the structure (see the Supporting Information). The environment around the metal center could therefore not be determined with good accuracy, and the bond lengths reported hold a substantial amount of uncertainty. We wholly refrain from reporting data about the bond angles around the iron center of complex **4** as we consider these values to give little insight to the given structure. The geometry of the extended ligands of the complex could, however, be ascertained. Molecular structures obtained through XRD can be found in Figure 2.

**Figure 2:**
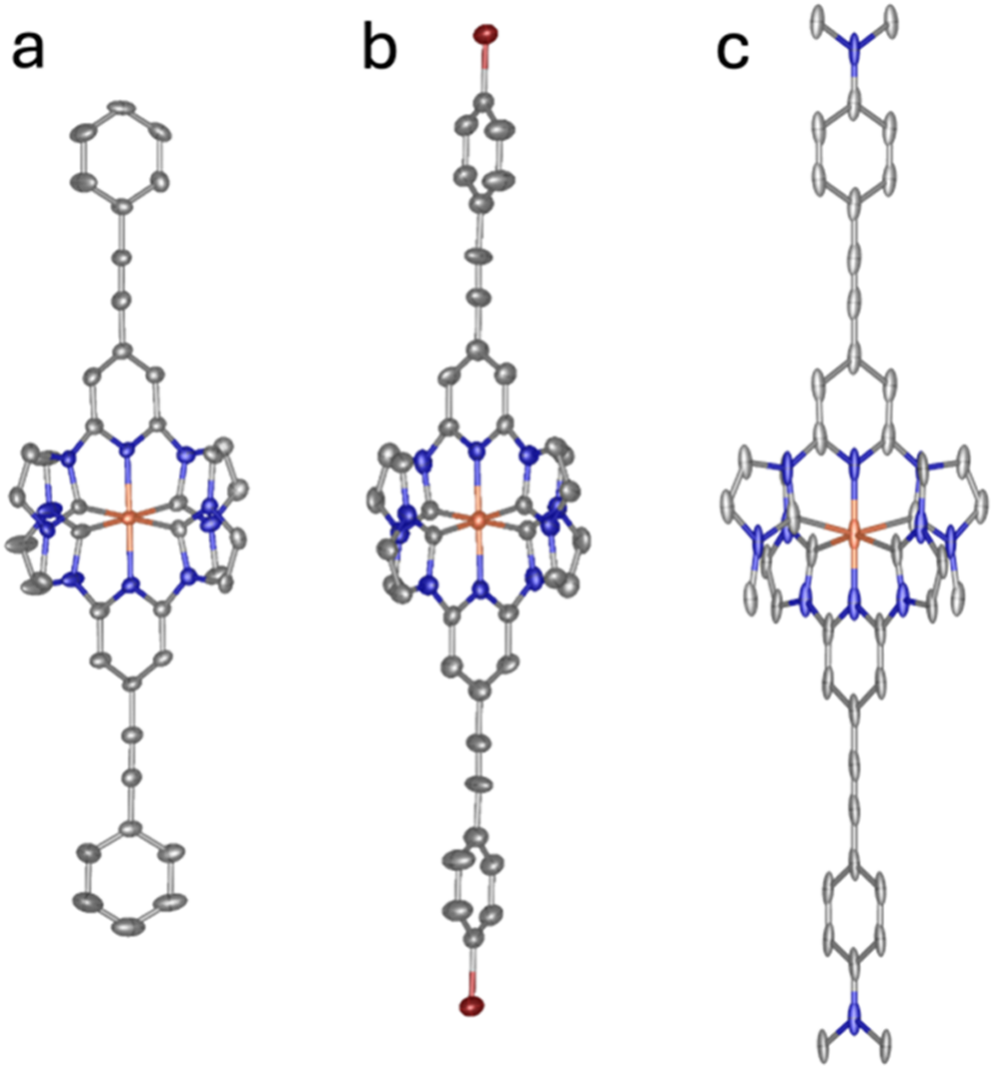
Molecular structures of (a) [Fe­(II)­(pepbmi)_2_]­(PF_6_)_2_ (**2**), (b) [Fe­(II)­(bepbmi)_2_]­(PF_6_)_2_ (**3**), and (c) [Fe­(II)­(depbmi)_2_]­(PF_6_)_2_ (**4**), as determined by SC-XRD. Counterions and solvent molecules are omitted for clarity. Displayed atoms are Fe, orange; C, gray/black; N, blue; Br, dark red.

Bond lengths and angles are largely unchanged by the introduction of a phenylethynyl group compared to parent complex **1**. The extended complexes retain the distorted octahedral geometry around the iron center, with the bond angles between carbene atoms in transposition being around 160° (Table 1). The dihedral angle between the phenylene ring and the pyridine ring is around 16° for complexes **2** and **3**, with complex **3** showing a slightly larger angle between the rings (Tables 1 and S2–S3). This suggests that at least a fair amount of electronic communication over the entire complex in the solid state could be possible. In complex **4**, the dihedral angle between the phenylene ring and the pyridine ring was shown to be significantly smaller, around 4°. Furthermore, a small angle (2.5°) was likewise found between the dimethylamino substituent and the phenylene ring (Table S4). This suggests that the electronic communication in complex **4** could be greater than for the other two phenylethynyl-substituted complexes.

**Table 1:** Average Selected Bond Lengths and Angles as Well as Angles between the Pyridine and Phenylene Rings, as Determined by SC-XRD Measurements^a^

complex	Fe–C (Å)	Fe–N (Å)	C–Fe–C_trans_ (°)	N–Fe–N (°)	Py–Ph (°)
[Fe(pbmi)_2_](PF_6_)_2_ (**1**)	1.967^b^	1.925^b^	158.3^b^	178.6^b^	
[Fe(pepbmi)_2_](PF_6_)_2_ (**2**)	1.943	1.901	158.9	179.4	15.2
[Fe(bepbmi)_2_](PF_6_)_2_ (**3**)	1.944	1.894	159.4	177.3	17.2
[Fe(depbmi)_2_](PF_6_)_2_ (4)	(2.043)	(1.972)			3.7

a Values in parentheses denote values of greater uncertainty due to low resolution of data for complex **4**.

b Values taken from ref 9.

### Mössbauer Spectroscopy

The fitting results for the ^57^Fe Mössbauer spectra of the iron-NHC samples at 295 and 85 K reveal a quadrupole split doublet structure (Figure 3). The fitting results are listed in Table 2.

**Figure 3:**
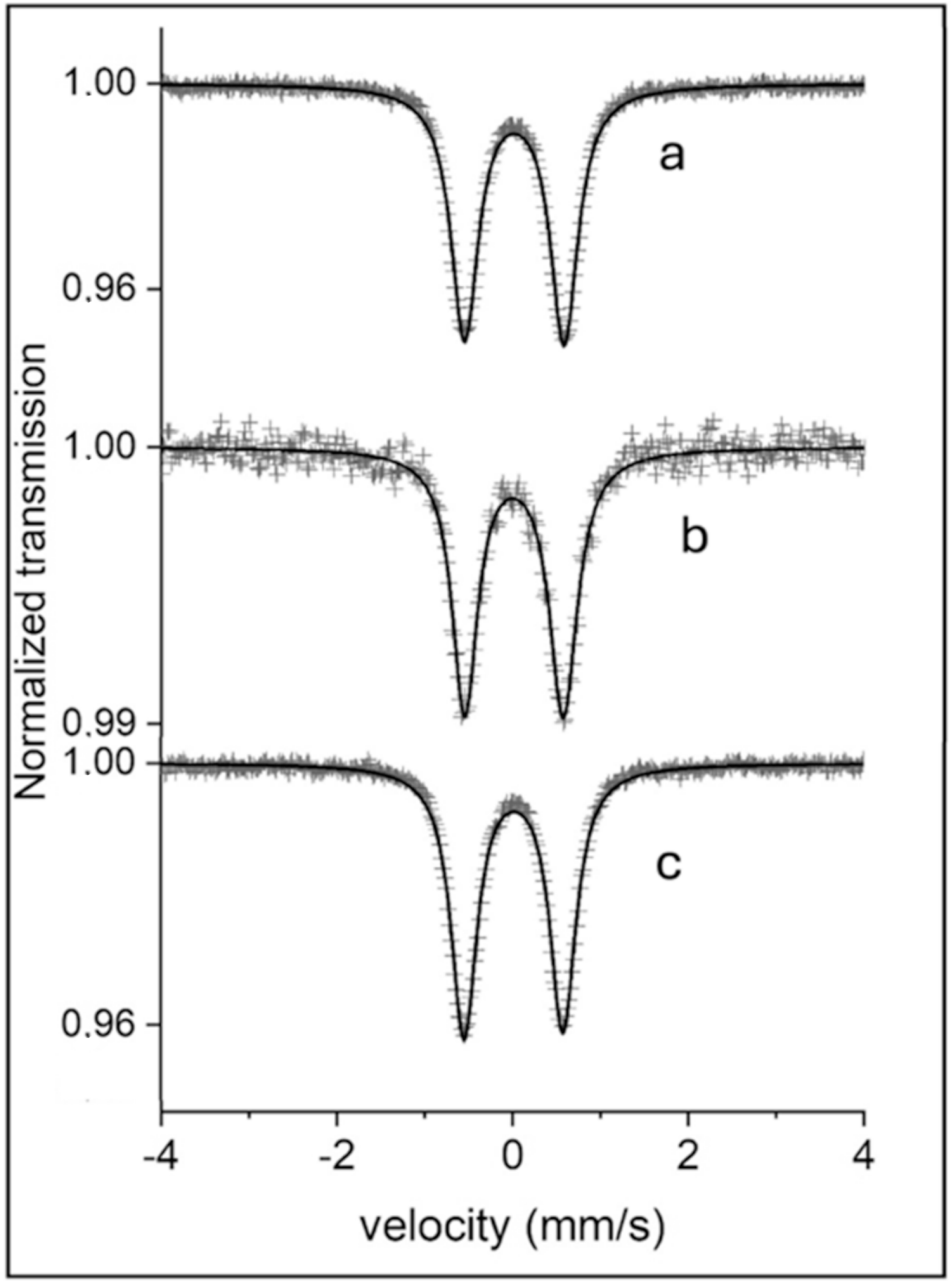
Mössbauer spectra of (a) [Fe­(pepbmi)_2_]­(PF_6_)_2_ (**2**), (b) [Fe­(bepbmi)_2_]­(PF_6_)_2_ (**3**), and (c) [Fe­(depbmi)_2_]­(PF_6_)_2_ (**4**). All complexes were recorded at 85 K.

**Table 2:** Results of the Fitting Procedure of the 85 K Mössbauer Spectra^a^

complex	CS (mm/s)	QS (mm/s)	W_+_(mm/s)
[Fe(pepbmi)_2_](PF_6_)_2_ (**2**)	0.017	1.126	0.360
[Fe(bepbmi)_2_](PF_6_)_2_ (**3**)	0.017	1.132	0.378
[Fe(depbmi)_2_](PF_6_)_2_ (**4**)	0.013	1.120	0.386

a CS is the center shift relative natural α-Fe held at 295 K, |QS| is the magnitude of the electric quadrupole splitting, and Γ is the FWHM Lorentzian line width. Error in these parameters: ± 0.005 mm/s.

The found center shifts (CS) and magnitude of the electric quadrupole splittings (|QS|) for Fe in all samples at 85 K (Table 2) fall close to the low-spin Fe­(II) values found for other samples measured in this type of Fe complexes.^30,32^

The center shifts found at 295 K are ∼0.08 mm/s lower than the values at 85 K, partly due to the second-order Doppler shift, between 295 and 85 K, which is ∼0.12 mm/s, assuming a Debye temperature θ_D_ = 300 K.

The largest contribution to the magnitude of |QS| in low-spin Fe­(II) complexes comes from the lattice contribution. In the present case, the near lattice surroundings for Fe are 4 C and 2 N atoms in a distorted octahedral configuration. If the two deviant atoms, the nitrogen atoms as in these cases, are in *trans* position, the contribution to the electric field gradient will be larger than for a *cis* orientation. This explains the relatively large |QS| for a low-spin Fe­(II) complex. Furthermore, the magnitude of the electric quadrupole splittings at 85 K is ∼0.07 mm/s larger than the corresponding values at 295 K. This increase in |QS| likely originates from the contraction of the lattice at lower temperatures.

It can thus be concluded that all of the iron-NHC samples can be described as low-spin Fe­(II) complexes. The assignments are based on quadrupole splitting and center shift values in connection with the ligand structure for the Fe atom. The resemblances in hyperfine parameters for the three samples reflect strong similarities in the near surroundings of the Fe ion in these complexes.

### Electronic Absorption Spectra

The absorption spectra of **2**–**4** in acetonitrile are illustrated in Figure 4. All three complexes exhibit strong absorption bands in the UV region (300–350 nm) that resemble the absorption of the ligand precursors (Figure S41) and can be attributed to ligand-centered (LC) π–π* transitions.^24^ Additionally, they show two distinct absorption features: one band with a maximum centered at 520 nm (ε = ∼35,000 M^–1^ cm^–1^) and a minor band at 400 nm (ε = ∼12,000 M^–1^cm^–1^), similar to parent complex **1**.^15^ For complexes **2** and **3**, both absorption bands in the 400–600 nm range are not observed with the corresponding ligand precursors and can be assigned to MLCT transitions, in agreement with the electrochemical data (see below) and previous assignments for related complexes.^15,30^ The significantly stronger 400 nm peak of complex **4** coincides however with a comparably intense absorption band of the dimethylamino ligand precursor [H_2_(depbmi)]­(PF_6_)_2_ (**12**). The latter is absent in the ligand precursors [H_2_(pepbmi)]­(PF_6_)_2_ (**10**) and [H_2_(bepbmi)]­(PF_6_)_2_ (**11**) and tentatively assigned to an intraligand charge transfer (ILCT) excitation from the amine lone pair to the electron-deficient C^∧^N^∧^C moiety. The 400 nm band of complex **4** might, therefore, emerge from a combination of ILCT and MLCT transitions.

**Figure 4:**
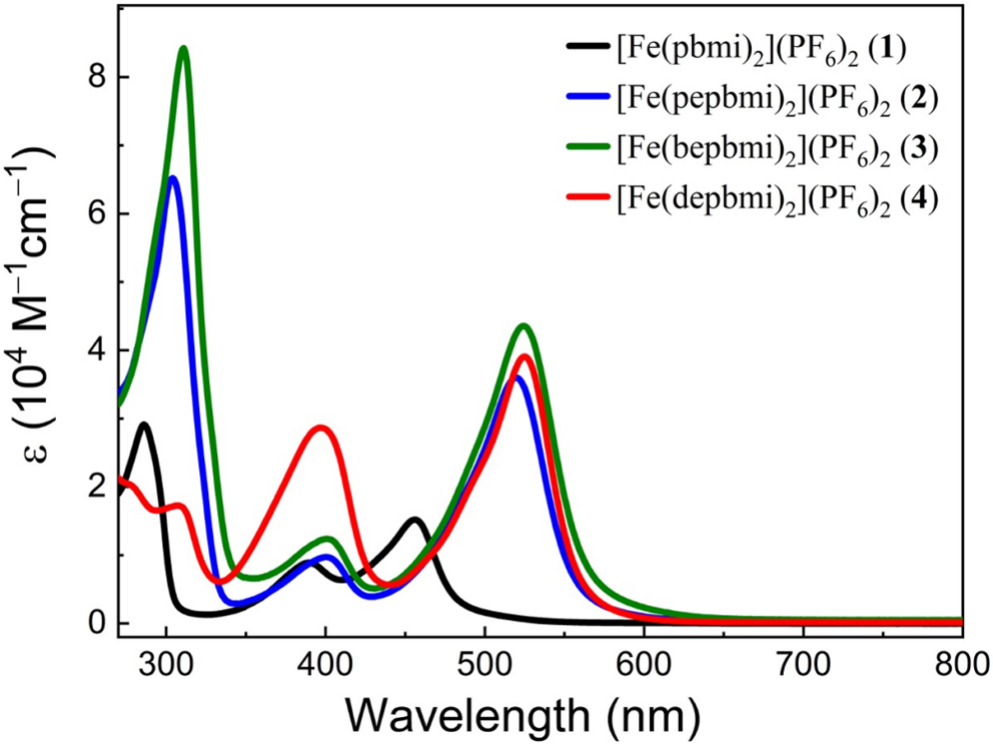
UV–vis absorption spectra of [Fe­(pbmi)_2_]­(PF_6_)_2_ (**1**), [Fe­(pepbmi)_2_]­(PF_6_)_2_ (**2**), [Fe­(bepbmi)_2_]­(PF_6_)_2_ (**3**), and [Fe­(depbmi)_2_]­(PF_6_)_2_ (**4**) in acetonitrile.

Relative to parent complex **1**,^15^ the introduction of phenylethynyl functional groups on the ligands of **2**–**4** leads to a significant red shift and an increased molar extinction coefficient of the lowest-energy absorption band.

The observed red shift of the charge transfer band can be rationalized by the electron-withdrawing character of the π-system of the phenylethynyl group and its effect on the potentials for the Fe­(III/II) couple and the first ligand reduction revealed by the electrochemical data (see below).

### Electrochemistry and Spectroelectrochemistry

The redox properties of complexes **2**–**4** were studied by cyclic and differential pulse voltammetry (Figure 5). Cyclic voltammograms of complex **2** exhibit a reversible wave at +0.46 V versus ferrocenium/ferrocene (Fc^+^/Fc) that can be assigned to the Fe­(III/II) couple. The shift toward positive potential by 150 mV compared to parent complex **1**, (Fe^(III/II)^ + 0.31 V^15^) can be attributed to the electron-withdrawing properties of the phenylethynyl moiety, yielding lower electron density at the iron center.^29,30^ The corresponding half-wave potentials for complexes **3** (+0.47 V) and **4** (+0.41 V) reflect the electron-withdrawing and electron-donating effects of bromo- and dimethylamino substituents. Complex **4** undergoes an additional oxidation close to that of the Fe­(III/II) couple that can be attributed to the amine substituents. The assignment of the first oxidation of **4** to the metal-centered couple is consistent with the expected shift in potential and the spectroelectrochemical data (see below). An even more pronounced effect of the phenylethynyl moiety is observed on the potential for the first ligand reduction of **2** and **4** that shifts by about +0.5 V relative to the parent complex. This results in estimates of the MLCT excitation energy that agree well with the position of the lowest-energy absorption bands (Table 3). The reductive voltammetry of the bromo-substituted complex (**3**) is, however, poorly resolved and provides no information on the potential of the first ligand-based reduction that correlates with the spectroscopic data.

**Figure 5:**
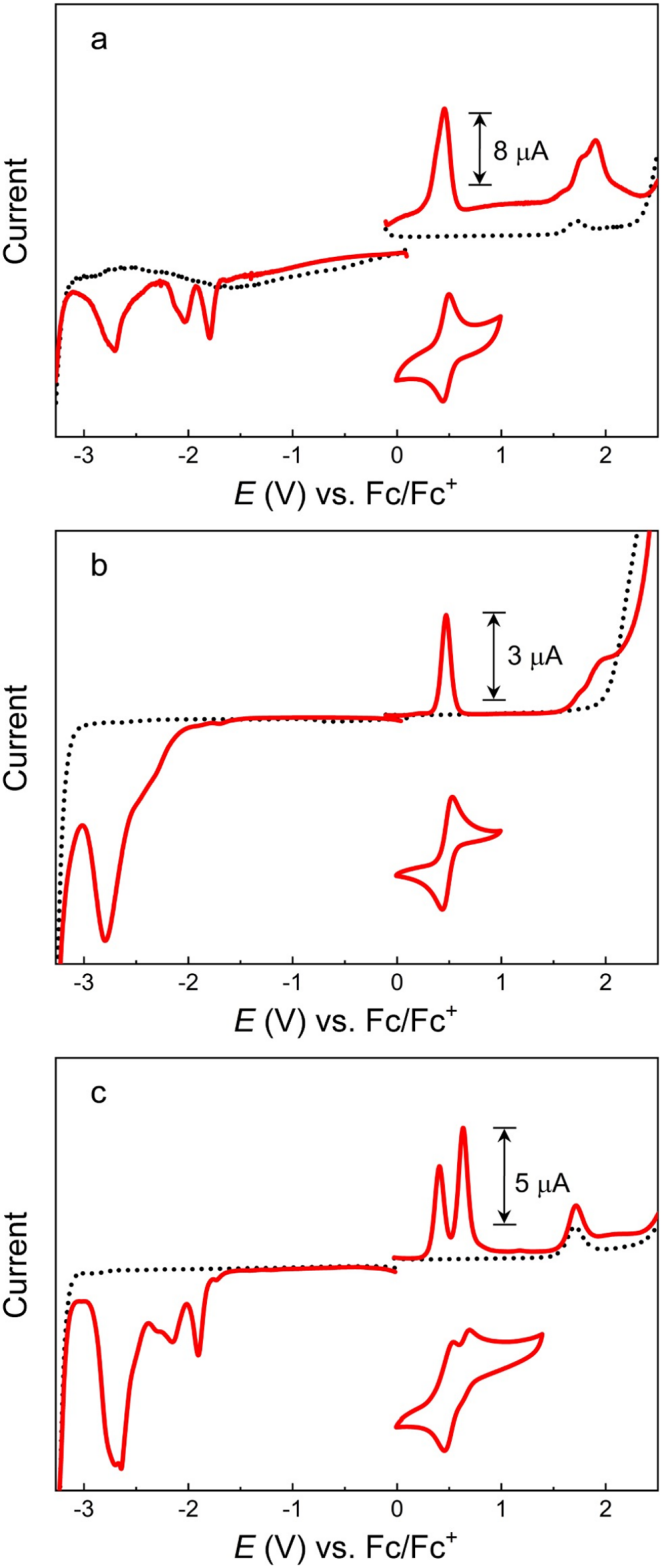
Differential pulse and cyclic voltammograms (0.05 V s^–1^) of (a) [Fe­(pepbmi)_2_]­(PF_6_)_2_ (**2**), (b) [Fe­(bepbmi)_2_]­(PF_6_)_2_ (**3**), (c) [Fe­(depbmi)_2_]­(PF_6_)_2_ (**4**), and electrolyte background (dotted black line). All complexes were present at 1 mM, in acetonitrile with 0.1 M tetrabutylammonium hexafluorophosphate (TBAPF_6_).

**Table 3:** Spectroscopic and Electrochemical Properties in Acetonitrile

		*E* (V)		
complex	λ_max_ (nm) (ε_max_(10^3^ M^–1^ cm^–1^))	Fe^III/II^ ^a^	L/L^•–^ ^b^	λ_MLCT, calc_ (nm)^c^
[Fe(pbmi)_2_](PF_6_)_2_ (**1**)	393 (9.0),^d^ 460 (15.9)^d^	+0.31^d^	–2.39^d^	459
[Fe(pepbmi)_2_](PF_6_)_2_ (**2**)	400 (9.7), 520 (36.0)	+0.46	–1.80	548
[Fe(bepbmi)_2_](PF_6_)_2_ (**3**)	400 (12.4), 524 (43.6)	+0.47	(−2.20)	
[Fe(depbmi)_2_](PF_6_)_2_ (**4**)	397 (28.6), 525 (39.1)	+0.41	–1.91	534

a CV half-wave potential vs Fc^+^/Fc.

b DPV peak potential vs Fc^+^/Fc.

c Estimated with Δ*E*_MLCT_ = *E*(Fe^III/II^) – *E*(L/L^•–^).

d Values taken from ref.^13^

The spectral characteristics of the oxidized and reduced complexes were investigated by spectroelectrochemistry (Figure 6a–c). Oxidation of the phenylethynyl-substituted complexes by controlled potential electrolysis leads to reversible spectral changes with clear isosbestic points. In all cases, oxidation of the complex leads to bleaching of the lowest-energy MLCT absorption band and the emergence of weaker, broad absorption, extending to about 780 nm. These changes are in agreement with the expected effects of metal-centered oxidation, and the low-energy product absorption can be attributed to an LMCT excitation of the resulting Fe­(III) state, consistent with the expected energy (∼1.5 eV) based on the potentials of the Fe­(III/II) couple and the first ligand-centered oxidation of **2** and **3**. The additional increase in absorption around 400 nm can be assigned to higher-energy CT transitions of Fe­(III) complexes. In the case of complex **4**, very weak product absorption extends into the near IR (NIR). The observation of very-low-energy LMCT involving oxidation localized at the amine substituent is however unlikely regarding the poor orbital overlap, and the NIR features are instead assigned to the aryl amine radical cation that is inevitably formed to some extent at potentials required for essentially exhaustive metal-centered oxidation of the complex. Interestingly, oxidation of **4** also leads to the bleaching of the intense 400 nm band characteristic of the amine-substituted complex and its ligand precursor. If the oxidation is correctly described as a metal-center process, then the 400 nm band of the complex should at least predominantly arise from CT transitions involving the metal center rather than LC transitions.

**Figure 6:**
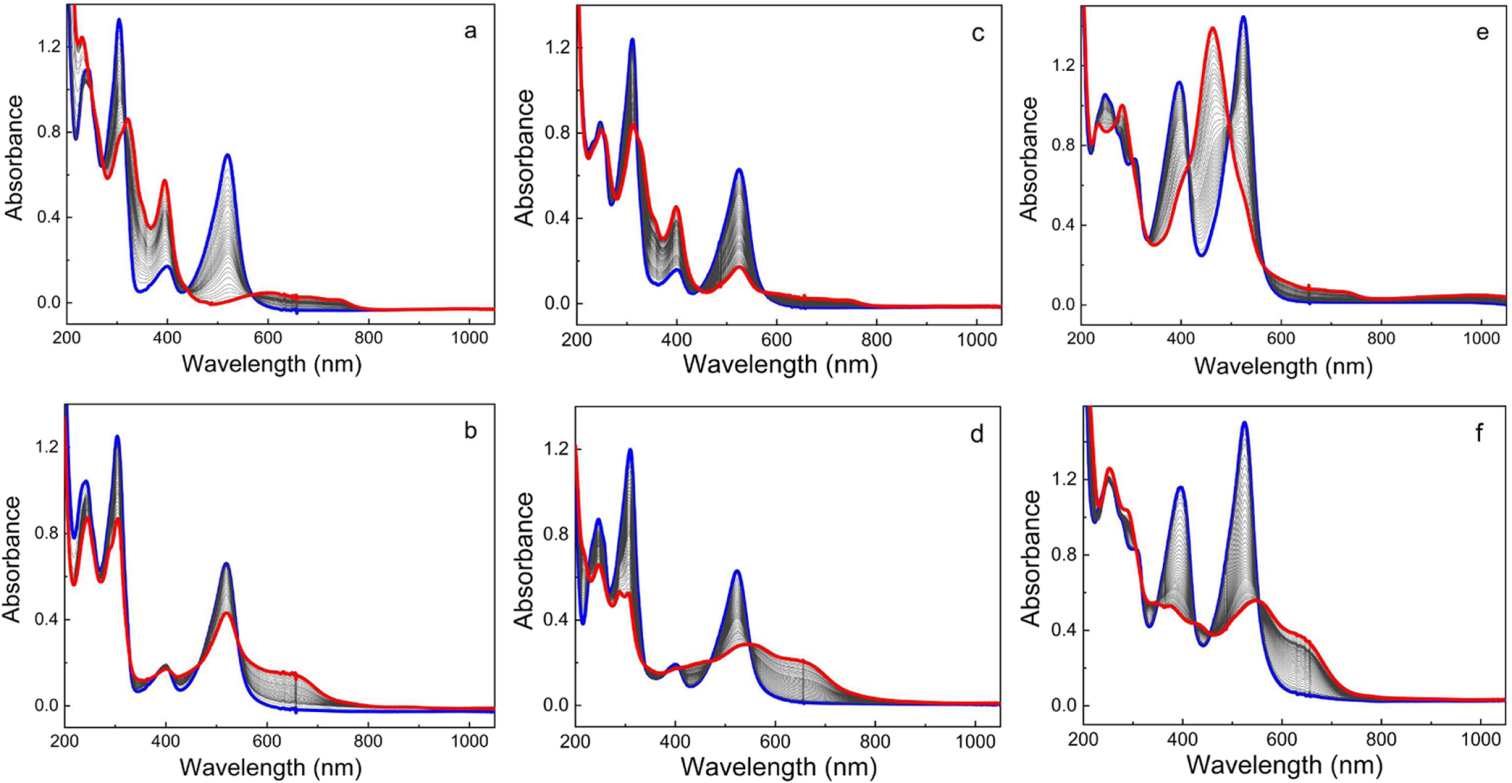
Spectroelectrochemistry monitoring changes in the optical absorption spectra (blue to red) in acetonitrile with 0.1 M TBAPF_6_ (optical path length, *l* = 1 mm). Left: [Fe­(pepbmi)_2_]­(PF_6_)_2_ (**2**) during metal oxidation at 0.70 V (a) and ligand reduction at −1.80 V (b). Middle: [Fe­(bepbmi)_2_]­(PF_6_)_2_ (**3**) during metal oxidation at 0.70 V (c) and ligand reduction at – 2.0 V (d). Right: [Fe­(depbmi)_2_]­(PF_6_)_2_ (**4**) during metal oxidation at 0.50 V (e) and ligand reduction at −2.0 V (f).

Ligand reduction of all phenylethynyl-functionalized carbene complexes leads to the expected bleaching of their MLCT bands and of part of the UV absorption associated with LC transitions. The product is primarily characterized by broad absorption extending to about 800 nm, with a peak around 550 nm and the lowest-energy shoulder at 650 nm. Several isosbestic points are maintained during electrolysis, indicating that the reduced complex does not undergo further transformations on the time scale of the spectroelectrochemisty experiments. The product spectra are hence associated with ligand radical species formed from Fe­(II) complexes.

### Quantum Chemical Calculations

Quantum chemical calculations showed that HOMO-2 through HOMO of complexes **2** and **3** exhibit a predominantly t_2g_ metal-based character, which is similar to parent complex **1**. In contrast to this, HOMO and HOMO-1 of complex **4** are predominantly localized on the phenylethynyl group, with Fe-based t_2g_ molecular orbitals (MOs) found at a lower energy (Figures S42–S43). This change is likely due to the electron-donating nature of the dimethylamino group, which destabilizes the energy of the ligand-localized π-MOs. The LUMO of all four complexes can be described as a ligand-based π* orbital associated with the C^∧^N^∧^C moiety of the ligand. LUMO energies are stabilized in complexes **2–3** relative to complex **1**, due to the presence of the phenylethynyl groups that extend π-conjugation of the C^∧^N^∧^C moiety of the ligand. The HOMO–LUMO gaps of complexes **2**–**3** also decrease relative to parent complex **1**, predominantly due to the stabilization of the LUMO energies (Figure S42), with **4** showing the smallest gap.

While the calculated spectra for complexes **2** and **3** provide a relatively good match for the experimental spectra, the calculated spectrum of **4** does not reproduce the experimental spectrum (see Figure S44). Looking more carefully at the spectra of **2** and **3**, we can observe a blue shift in the calculated MLCT bands (at 460 nm for **2** and 470 nm for **3**) relative to the experimental spectrum (520 nm for **2** and **3**). On the other hand, the calculated spectra for the protonated ligands are red-shifted with the LC transitions at 350 nm for **2** (vs 300 nm experimental) and 360 nm for **3** (vs 300 nm experimental). Despite the blue shift of the MLCT bands and the red shift of the LC bands, the MLCT bands of complexes **2** and **3** are still calculated at lower energies than the LC bands, leading to overall acceptable shape of the calculated spectra.

Analyzing the spectrum of the uncoordinated ligand (**12**) of complex **4** (see Figure S44), the red shift in the calculated ILCT transition vs the experimental spectrum is more significant than for complexes **2** and **3**, which is likely due to the presence of the strongly donating dimethylamine substituent. As shown in Figure S44, the lowest-energy transition corresponding to HOMO–LUMO excitation for complex **4** is ILCT, appearing at around 500 nm. This is consistent with similar peaks appearing in the same region of the ligand spectrum. However, MLCT states are calculated at a higher energy (400 nm) due to lower t_2g_ set in complex **4** than that in complexes **2** and **3**, which results in a blue shift when compared to the experiment. The discrepancy between the calculated and experimental spectrum of complex **4** is likely due to the TD-DFT underestimating the energy of the ILCT transitions,^33^ while slightly overestimating the energies of the MLCT bands.

The spectra of complexes **2**–**4** have been calculated at different levels of theory to see if it is possible to mitigate the problem with the red shift of the ILCT transitions vs the blue shift of the MLCT transitions. Unfortunately, the use of various functionals, including the long-range corrected functionals such as CAM-B3LYP, results in the calculated spectra of similar shape (and incorrect ordering of the MLCT vs ILCT states for complex **4**), as those obtained with the B3LYP functional. Additionally, we examined the impact of conformational flexibility on all three complexes by rotating the phenylethynyl substituent to a perpendicular position relative to the C^∧^N^∧^C moiety of the ligand (see Figure S47). The rotational barriers are around 1.5 kcal/mol for **2** and **3** and 2.7 kcal/mol for **4**, indicating that such ligand rotations are feasible. Averaging the UV–vis spectra of different conformers revealed minor effects on peak intensities rather than their positions (see Figure S48), suggesting that the observed spectral shifts are more likely due to DFT inaccuracies rather than conformational flexibility.

Despite the challenges with reproducing the experimental UV–vis spectrum of complex **4**, our DFT calculations on complexes **2**–**4**, taken together with the calculated spectra on the ligands alone (see Figure S44), allow us to assign the peaks at ∼525 nm in the experimental spectrum (see Figure 4) as the MLCT transitions, while the peaks at ∼400 nm are due to the π to π* transitions (with a mixture of ILCT and LC character) on carbene ligands. Due to the strongly electron-donating character of the dimethylamino substituent in **4**, this transition acquires a more significant charge transfer character than that in complexes **2** and **3**.

### Transient Absorption Spectroscopy

Femtosecond transient absorption spectroscopy (fs-TA) measurements were performed to probe the excited-state dynamics of phenylethynyl-functionalized complexes [Fe­(pepbmi)_2_]­(PF_6_)_2_ (**2**), [Fe­(bepbmi)_2_]­(PF_6_)_2_ (**3**), and [Fe­(depbmi)_2_]­(PF_6_)_2_ (**4**) in acetonitrile solution. Figure 7 shows the time-dependent spectral evolution of the fs-TA signal following excitation of complexes **2**, **3**, and **4** into their MLCT bands with a 525 nm pump pulse. Essentially, identical TA data was observed upon excitation at 400 nm (Figures S50–S52). For all three complexes, the initially observed TA spectra include an intense ground-state bleach (GSB) between 450 and 550 nm and broad excited-state absorption (ESA) in the >550 nm range. Additional ESA bands of similar intensity at about 340 and 420 nm together with a very minor net bleach of their weak 400 nm GS absorption are common to complexes **2** and **3**. In the case of complex **4**, a more pronounced net bleach corresponding to its intense 400 nm GS absorption band and a weaker 340 nm ESA band are observed. Comparing the early dynamics, the 420 nm ESA band of **2** and **3** decays to a large extent within the first 0.3 ps, whereas the strong 440 nm ESA band of complex **4** persists on the picosecond time scale and the subpicosecond dynamics is mostly reflected in a slight blue shift of this band. For all three complexes, the TA spectra resemble strongly the MLCT reference spectra composed of spectroelectrochemical data for their metal-centered oxidation and ligand-centered reduction (Figure 7a–c).^34^ For complexes **2** and **3**, the agreement with the MLCT reference spectra is further improved after the initial subpicosecond process, and the TA spectra observed on the picosecond time scale are therefore attributed, at least predominantly, to a relaxed ^3^MLCT state that forms via ultrafast relaxation of a hot MLCT precursor (*MLCT) on the subpicosecond time scale. With intersystem crossing (ISC) from the initially populated ^1^MLCT typically occurring on a sub-100 fs time scale in Fe­(II) complexes,^35^ this process is presumably not resolved with our time resolution. The assignment to the ^3^MLCT state is further corroborated by the good agreement with TA data previously reported for a carboxylic acid derivative of **1**.^31,36,37^ For this complex, TA spectra and lifetime resemble strongly the results obtained with **2**–**4**, and unambiguous evidence for the ^3^MLCT assignment has been obtained from vibrational coherence spectroscopy^36^ and fluorescence up-conversion spectroscopy.^38^

**Figure 7:**
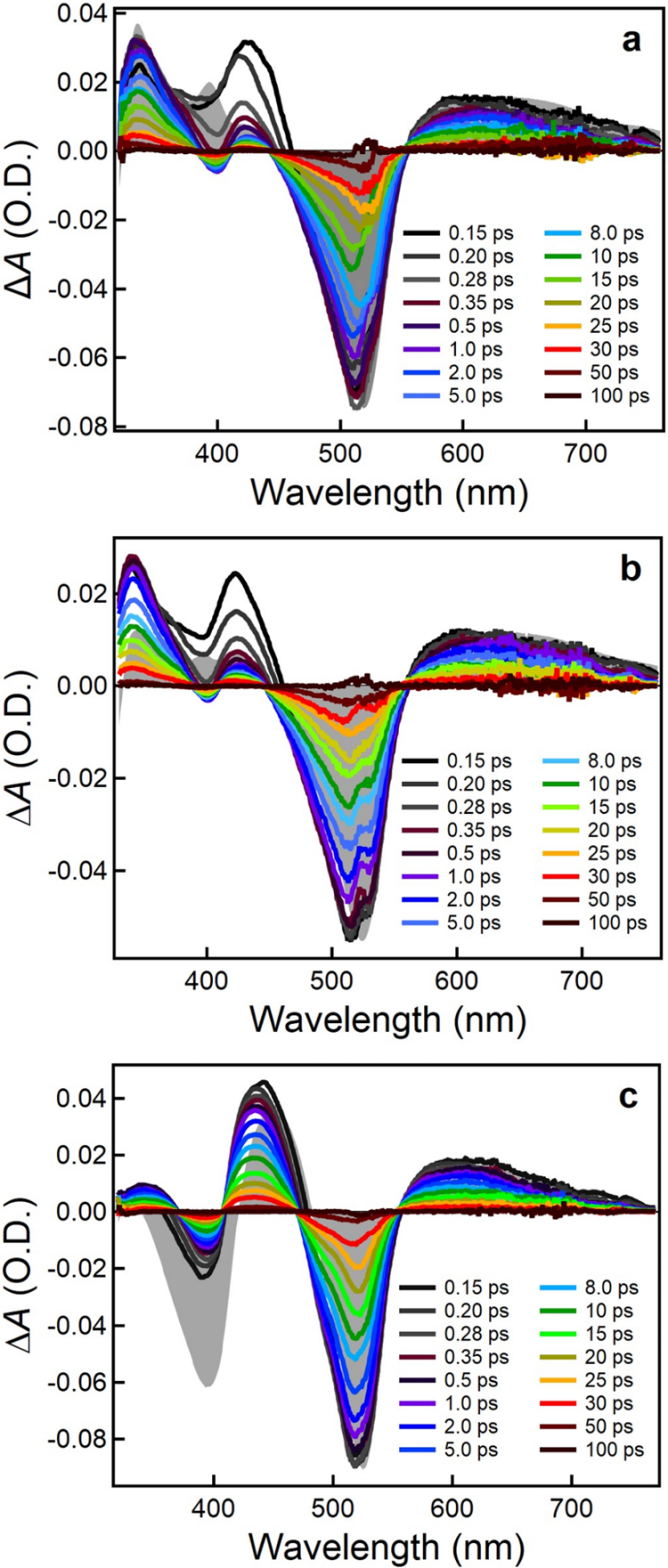
Transient absorption spectra at selected delay times of (a) [Fe­(pepbmi)_2_]­(PF_6_)_2_ (**2**), (b) [Fe­(bepbmi)_2_]­(PF_6_)_2_ (**3**), and (c) [Fe­(depbmi)_2_]­(PF_6_)_2_ (**4**). MLCT reference spectra (shaded area) from spectroelectrochemistry. All complexes in acetonitrile, excitation wavelength 525 nm.

Within 100 ps, also the longer-lived ESA features and the GSB decay essentially completely back to baseline, indicating quantitative recovery of the ground state and the absence of irreversible photoprocesses or longer-lived dark states. The decay is however biphasic and kinetic fits by global analysis require in total three exponential terms returning for all three complexes time constants of about 0.2, 3, and 15–20 ps, respectively (Table 4; see SI for fit results based on parallel or sequential fit models). Next to the ^3^MLCT state, also the ^3^MC state of Fe­(II)­NHC complexes might contribute to ESA in the visible spectral range with lifetimes in the low picosecond regime.^29,39^ Furthermore, it has been previously demonstrated that the latter state can be directly formed from an unrelaxed MLCT precursor, *i.e*., in parallel to the relaxed ^3^MLCT state of complex **1**,^40^ and its carboxylic acid derivative.^36^ Also for complexes **2**, **3**, and **4**, a corresponding kinetic model (Figure 9) resulted in good agreement between the fit results and the experimental TA traces across all wavelengths (Figure 8d–i). Species-associated differential spectra (SADS) returned by this model are shown in Figure 8a–c next to the MLCT reference spectra. For all three complexes, the decay of the initially observed species with SADS-1 is limited by the instrument response (∼150 fs) and gives rise to a long-lived (17 ps) successor described by SADS-3 and, in a parallel reaction with a branching ratio of about 1:2, to a third species characterized by SADS-2 with lifetimes ranging from 3 to 6 ps for the three complexes (Table 4). The TA data is therefore in line with excited-state dynamics analogous to those previously reported for the carboxylic acid derivative of parent complex **1**.^36^ Also, for the latter complex, it has been shown earlier that electron-withdrawing carboxylic acid substituents result in excited-state dynamics that are dominated by a relatively longer-lived relaxed ^3^MLCT state (19 ps) next to a shorter-lived ^3^MC state (3 ps) that forms in comparable amounts from a common precursor.^36^ Additional formation of the ^3^MC state via the long-lived ^3^MLCT state is in this situation slower than the ^3^MC decay (inverted kinetics) and therefore has little effective contribution to the ^3^MC population.

**Figure 8:**
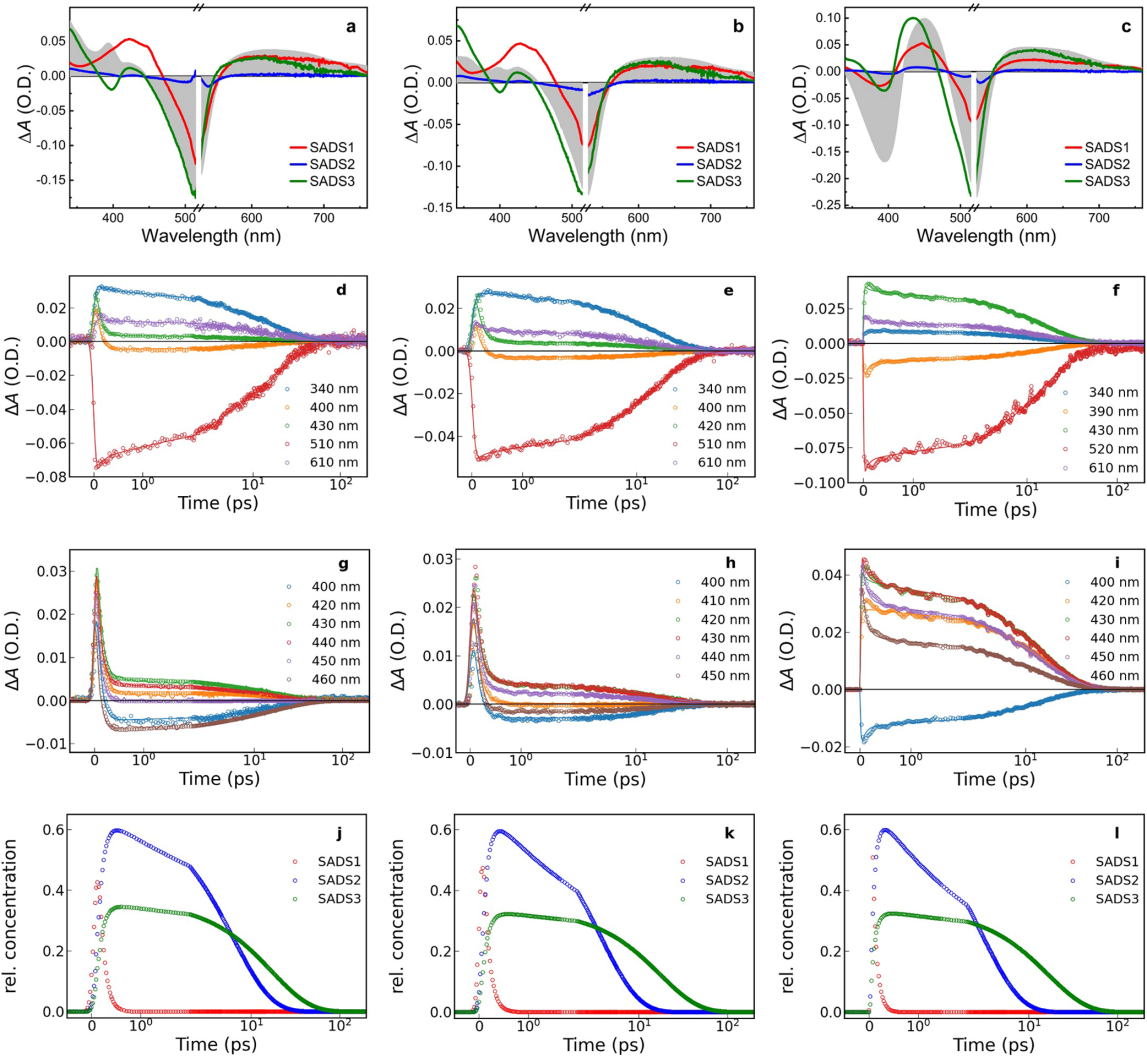
Global analysis results for the transient absorption spectra shown in Figure 7 based on target analysis according to the scheme in Figure 9. Left column: [Fe­(pepbmi)_2_]­(PF_6_)_2_ (**2**), middle column: [Fe­(bepbmi)_2_]­(PF_6_)_2_ (**3**), right column: [Fe­(depbmi)_2_]­(PF_6_)_2_ (**4**). (a–c) Species-associated difference spectra (SADS) and MLCT reference spectra (shaded area) from spectroelectrochemistry. (d–i) Transient absorption kinetics fit results (solid lines) at selected wavelengths. (j–l) Concentration kinetics.

**Table 4:** Excited-State Lifetimes^a^

complex	τ_1_	τ_2_	τ_3_
[Fe(pepbmi)_2_](PF_6_)_2_ (**2**)	<0.2 ps	6.2 ps	17.1 ps
[Fe(bepbmi)_2_](PF_6_)_2_ (**3**)	<0.2 ps	3.6 ps	17.4 ps
[Fe(depbmi)_2_](PF_6_)_2_ (**4**)	<0.2 ps	3.0 ps	16.5 ps

a Values obtained for the corresponding species associated difference spectra (SADS, Figure 8) from global fitting of TA data (Figure 7) according to the scheme in Figure 9.

In summary, the TA data indicates that **2**–**4** are characterized by a relatively long-lived ^3^MLCT state similar to the carboxylic acid and imidazolium derivatives.^29,30^ For parent complex **1**, clearly shorter ^3^MLCT lifetimes of 9 ps have been consistently determined from optical TA spectroscopy^13^ and time-resolved X-ray spectroscopy and scattering data.^40^ A recent report based on optical TA spectroscopy that attributes the ps dynamics to the ^3^MC state concludes that the ^3^MLCT state is deactivated even faster on the subpicosecond time scale.^29^ In analogy to the carboxylic acid and imidazolium derivatives, the longer ^3^MLCT lifetimes of **2**–**4** can be attributed to the electron-withdrawing effect of the phenylethynyl substituents that lower the ^3^MLCT energy to almost exactly the same extent as the carboxylic acid substituents judged by the very similar red shift of the lowest-energy MLCT absorption band relative to the parent complex.

**Figure 9:**
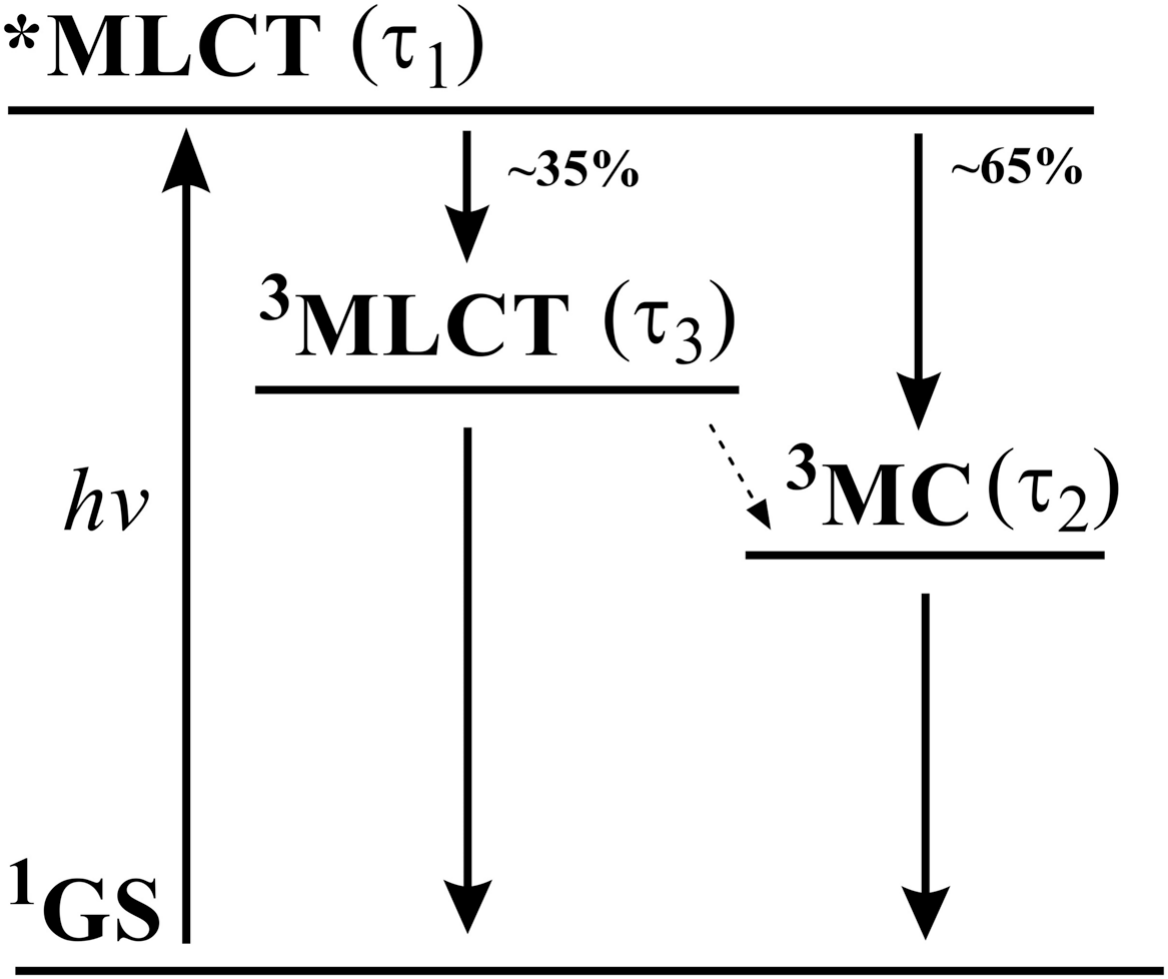
Schematic representation of the proposed excited-state relaxation of complexes **2**, **3**, and **4**.

## Conclusions

Three different iron-NHC complexes (**2**–**4**), with phenylethynyl moieties, were synthesized and characterized with respect to their ground- and excited-state properties. The introduction of these moieties on the imidazolylidene-type NHC ligands led to a significant stabilization of the ^3^MLCT states as apparent from the strongly red-shifted MLCT absorption bands, compared to parent unsubstituted complex **1**. This effect can be attributed to the electron-withdrawing effect of the phenylethynyl moieties, which could be slightly modulated with bromo or amino substituents on the phenyl rings. All three complexes are characterized by a longer-lived (17 ps) ^3^MLCT state, similar to what was reported for a carboxylic acid and imidazolium derivatives of the parent complex. These findings support the notion that the excited-state dynamics of iron complexes derived from parent complex **1** can be tuned toward extended ^3^MLCT lifetimes by electron-withdrawing substituents that adjust the ^3^MLCT energy to about 2.3 eV or below to disfavor ultrafast deactivation via MC states.

## Experimental Section

### Synthesis

In brief, precarbene ligand salt (**10**, **11**, or **12**; 2 equiv) was dried and suspended in THF. The resulting suspension was cooled, and to it was added base (KO*t*-Bu for **10** and **12**, LDA for **11**; 5 equiv). To the resulting solution was added FeBr_2_ (1 equiv), dissolved in THF. The resulting mixture was evaporated, and to the resulting residue were added methanol and hydrochloric acid. The complex was precipitated by addition of ammonium hexafluorophosphate (>40 equiv), followed by water. The crude product was purified twice by size-exclusion chromatography (BioBeads S-X1, MeCN/PhMe 1:1), collecting the red fractions. Detailed synthetic procedure of all new presented compounds is found in the Supporting Information.

### Single-Crystal X-ray Structure Determination

Suitable crystals for SC-XRD measurements were loaded on an Agilent Xcalibur Sapphire3 diffractometer high-brilliance IμS radiation source using graphite-monochromatized Mo Kα radiation (λ = 0.71073 Å). All of the structures were solved by direct methods using SHELXS-97 and refined by full-matrix least-squares on F2 using SHELXL-97. The details pertaining to the data collection and refinement for complexes **2** and **3** are given in Table S1. Nonhydrogen atoms were refined with anisotropic displacement parameters. All of the hydrogen atoms were included in idealized positions, and their positions were refined isotropically by a riding model. Nonhydrogen atoms were refined anisotropically. Complex **2** crystallized with 1.5 molecules of CH_3_CN as solvent of crystallization per formula unit. The OLEX2 solvent masking was used to treat diffuse scattering in complex **1**, which showed the presence of 250 electrons in a volume of 1418 Å3 in 4 voids per unit cell. This is consistent with the presence of 0.875 [CH_3_CN] and 0.25 [toluene] per formula unit, which account for 254 electrons per unit cell.^2^ A solvent mask was calculated for complex **2**, and 314 electrons were found in a volume of 1838 Å^3^ in 2 void per unit cell. This is consistent with the presence of 1.5 [CH_3_CN] and 1­[toluene] per formula unit, which account for 332 electrons per unit cell.

### Spectroscopy

Steady-state absorption measurements were performed on a Varian Cary5000 spectrophotometer. The complex was weighed and dissolved in filtered acetonitrile collected from a dry solvent dispenser (Sigma-Aldrich). The same quartz-glass cuvette of path length 1 mm (Hellma – Optical Special Glass) is used for the pure solvent baseline correction. The extinction coefficient was evaluated, where appropriate, by performing a linear fit to the absorbance as a function of concentration for each wavelength after the background had been corrected.

Femtosecond-TA spectra were measured on a Newport TAS system with Coherent Libra Ti:sapphire Amplifier, with the central output wavelength 800 nm, and 3 kHz repetition rate, delivering ∼40 fs pulse with power 1.5 mJ. The amplifier output is divided into two parts, one part is the pump, a collinear optical parametric amplifier (TOPAS-C, Light Conversion). The TOPAS generates a pump beam wavelength (525 nm), used as excitation to the sample. While the probe light is generated by the fundamental 800 nm beam, it was focused onto a 5 mm CaF2 crystal to generate a white supercontinuum (broadband) probe beam. The delay between pump and probe beams was introduced by a computer-controlled delay stage (Aerotech) placed in the probe beam’s path. The pump and probe light are being focused to an ∼100 μm spot size and overlapping with the pump pulse in the sample volume. After passing the sample, the probe beam is collimated again and relayed onto the entrance slit of a prism spectrograph. The reference beam is directly relayed on the said spectrograph. Pump–probe overlap was optimized at the sample, and the pump power was adjusted to ca. 1 mW. The intensity of excitation pulses was set to roughly 1 mW. Mutual polarization between pump and probe beams was set to the magic angle (54.7°) by placing a Berek compensator in the pump beam.

A solution of the phenylethynyl complexes in acetonitrile solvent was filled in a 1 mm optical path length quart cuvette, and measurements are performed at room temperature. No photodegradation was noticed in the sample, confirmed by the sample steady-state absorption spectra measured before and after TA experiments. Igor8.1 was used for chirp correction, and the corrected data were used for the global analysis using a Python script provided by Johannes Wega (University of Geneva).

### Electrochemistry

Electrochemical and spectroelectrochemical measurements were carried out in a standard three-electrode setup consisting of working (1 mm diameter, glassy carbon, CH Instruments), counter (platinum rod in a separate compartment), and reference electrodes (0.01 M Ag^+^/Ag). Spectroscopic-grade acetonitrile dried for 48 h over 3 Å activated molecular sieves was used as the solvent, together with 0.1 M tetrabutylammonium hexafluorophosphate (electrochemical grade, Sigma) dried for 24 h under vacuum at 80 °C as the supporting electrolyte. Sample solutions were deaerated by purging with solvent-saturated argon gas. Cyclic voltammograms were recorded at 0.05 V/s and differential pulse voltammograms with step potential: 5 mV, modulation amplitude: 25 mV, modulation time: 0.05 s, interval time: 0.1 s. UV–vis spectroelectrochemistry was carried out during controlled potential electrolysis in the same cell by switching the working electrode to a platinum mesh electrode placed in the 1 mm optical path. An Autolab potentiostat (PGSTAT302) was used to control the three-electrode setup using GPES 4.9 software, and an Agilent 8453 diode array spectrophotometer was used to record the spectral traces.

### Quantum Computation

All complexes investigated were optimized in their singlet ground states using the B3LYP^3^ functional and Grimme’s D2 dispersion correction.^41–45^ The Stuttgart/Dresden (SDD) effective core potential and its corresponding basis set were utilized for Fe and Br,^46^ while the 6–311G* basis set was employed for all other atoms (H, C, N, O).^47^ A polarizable continuum model (PCM) was utilized to include acetonitrile as the implicit solvent.^48^ All calculations on the molecular complexes were carried out using the Gaussian 16 software package.^49^

Fragment orbital (FO) analysis as implemented in AOMix was performed to quantify the amount of MO localization on the metal (Fe), C^∧^N^∧^C ligand-part and phenylethynyl ligand fragment.^50^ An MO was designated metal-based, C^∧^N^∧^C-based, or phenylethynyl-based if 60% or more of the electron density was localized on one of the assigned fragments. Some MOs show mixed C^∧^N^∧^C and phenylethynyl ligand fragments.

Absorption spectra were calculated with time-dependent DFT with additional Tamm–Dancoff Approximation (TDA),^51^ at several levels of theory including B3LYP,^41^ TPSSh,^52,53^ and CAM-B3LYP.^54^

## Supplementary Material



## Notes

Synthetic experimental details; ^1^H, ^13^C, and 2D NMR spectra for all compounds; additional information on crystal structure refinement data and methodology; absorption spectra of compounds **10–12** and complementary TA data for complexes **2–4**; Frontier orbital energy-level diagrams, Frontier MOs, UV/vis spectra, and potential energy curves, as obtained by quantum chemical calculations (PDF)
