# Supplementary material for: Iron N‑Heterocyclic Carbene Photoactive Complexes with Rigid Phenylethynyl Substituents as Ligand π‑System Extensions
Source: Inorg Chem. 2025 Jun 9;64(24):12120–31. doi: 10.1021/acs.inorgchem.5c01461 (PMC12188558; doi:10.1021/acs.inorgchem.5c01461)
Supplement: Supplementary file 1 [file ic5c01461_si_001.pdf]

Supplementary information for Iron *N*-Heterocyclic Carbene Photoactive complexes with Rigid Phenylethynyl Substituents as Ligand π-System Extensions

Samuel Persson[a†], Raj Kumar Koninti[b†], Mariam Barakat[c], Abhishek Mishra[a], Fredrik Lindgren[d], Tore Ericsson[d], Lennart Häggström[d], Sven Lidin[a], Ana Gonzalez[e], Elena Jakubikova[c]*, Reiner Lomoth[b]*, Kenneth Wärnmark[a]*

[a]Centre for Analysis and Synthesis, Department of Chemistry, Lund University, Box 124, SE-22100 Lund, Sweden. E-mail: kenneth.warnmark@chem.lu.se

[b]Department of Chemistry – Ångstrom Laboratory, Uppsala University, Box 523, SE-75120 Uppsala, Sweden. E-mail: reiner.lomoth@kemi.uu.se

[c]North Carolina State University, Department of Chemistry, Raleigh, NC 27695, USA. E-mail: ejakubi@ncsu.edu

[d]Department of Physics – Ångström Laboratory, Uppsala University, Box 530, SE-751 21 Uppsala, Sweden

[e]BioMAX, MAX IV, Lund University, Box 188, SE-221 00 Lund, Sweden.

†These authors contributed equally to this work.

*Corresponding author.

Table of contents

Synthesis and structure identification **1**
NMR spectra 6
Single Crystal X-ray Structure Determination 25
Steady-State Absorption Spectra **28**
Quantum chemical calculations **28**
Additional Analysis of the Calculated UV-Vis Spectra 29
Trends in the Excited-State Energetics 31
Transient absorption spectroscopy **33**
References **36**

Synthesis and structure identification

General. All commercial reagents and solvents were used as received, unless otherwise stated. NaH (60 % in mineral oil), imidazole, $Pd(PPh_3)_2Cl_2$ and CuI were purchased from Merck and stored in desiccator. NaH was additionally washed three times before use; this was done by stirring the reagent with dry hexane, obtained from MBraun MB-SPS 800 solvent purification system, before decanting of the solvent. Diisopropylamine (DIPA) was purchased in septa-fitted bottles from Merck. Hydrochloric acid (concd. aq.) was purchased from VWR. 2,6-Difluoro-4-iodopyridine was purchased from AmBeed (via Chemtronica) and stored in freezer. 1-Bromo-4-ethynylbenzene was purchased from TCI. Phenylacetylene and iodomethane (MeI) was purchased from Merck and stored in refrigerator. *n*-BuLi (2.5 M solution in hexanes) and KO*t*-Bu (1 M solution in THF) was purchased in septa-fitted bottles from Merck and stored in refrigerator. Methyl triflate was purchased from TheromScientific, fashioned with septum under Argon and stored in refrigerator. Ammonium hexafluorophosphate (NH_4PF_6) was purchased from Apollo Scientific and stored in desiccator. Anhydrous iron(II) bromide ($FeBr_2$) was

purchased from Alfa Aeser and stored and weighed under nitrogen in a glovebox. Dry Dichloromethane (DCM) was obtained from MBraun MB-SPS 800 solvent purification system. THF without stabilizer was dried over Na/benzophenone and subsequently distilled under N_2, further dried over activated molecular sieves (3Å) before use. DMF was dried over activated molecular sieves (3 Å) for approx. 1 h before use. Reactions run at room temperature (rt) are in the range of 18-23 °C. Metal heating mantels were used to achieve required reaction temperatures. Air- and moisture-sensitive reactions were carried out in oven-dried glassware capped with rubber septa, under a positive pressure of nitrogen. Flash column chromatography (⌀ x h) was carried out on silica gel (60 Å, 230-400 mesh, purchased from Merck) using reagent-grade eluents. Size exclusion chromatography (⌀ x h) was carried out on BioBeads S-X1 (200-400 mesh purchased from Bio Rad). Before loading on size exclusion column, all samples were dissolved in minimal amount of eluent and filtered through syringe filter (Fisherbrand, PTFE, 0.45 µm porosity, 13 mm diameter)). Analytical thin-layer chromatography (TLC) was carried out on TLC Silica gel 60 F254 and visualized with a UV-lamp (254 nm/365 nm). ^{1}H NMR and ^{13}C NMR spectra were recorded at rt on a Bruker Avance II spectrometer at 400.1 MHz and 100.6 MHz, respectively. The spectra were recorded in $CDCl_3$ or CD_3CN and the residual solvent signals (7.26/77.16; 1.94/118.26 ppm respectively) were used as a reference. Chemical shifts (δ) are expressed in parts per million (ppm) and coupling constants (J) are reported in Hertz (Hz). The following abbreviations are used to indicate apparent multiplicities: s, singlet; br, broad; d, doublet; dd, doublet of doublets; dq, doublet of quartets; t, triplet; td, triplet of doublets, q, quartet, m, multiplet. Melting points were measured using a Stuart melting point apparatus SMP3 and were corrected against 3,5-dinitrobenzoic acid (used for **7**, **8**, **9**, **10**, and **12**; mp 205 °C[S1]), saccharine (used for **2**, **3**, and **6**; mp 229 °C[S2]), 4-methoxybenzoic acid (used for **11**; mp 184 °C[S2]) or Benzoic acid (used for **4**; mp 122 °C[S2]). IR transmittance spectra were recorded on a Bruker-ALPHA II FT-IR Spectrometer and reported in cm^{-1} with the following abbreviations used to report relative signal strength: w, weak; m, medium; s, strong; vs, very strong; and br, broad. High resolution mass spectrometry was carried out using a Waters - XEVO-G2 QTOF spectrometer employing electrospray ionization and run in positive mode. Elemental analyses were performed by A. Kolbe, Mikroanalytisches Laboratorium, Germany.

2,6-Di(imidazol-1-yl)-4-iodopyridine (6). To a solution of NaH (446 mg, 18.6 mmol) in dry DMF (43 mL), cooled to 0 °C, was added imidazole (1134 mg, 16.66 mmol) and the resulting mixture was stirred at 0 °C for 1 h. The reaction was allowed to warm to rt and to it was added 2,6-difluoro-4-iodopyridine (2019 mg, 8.378 mmol) and the resulting solution was heated to 50 °C and stirred for 18 h. The reaction was poured into water (200 mL) and the resulting mixture was extracted with chloroform (3 x 200 mL). The combined organic phases were consecutively washed with water (400 mL) and dried over $MgSO_4$. The solution was evaporated *in vacuo* and the residue was consecutively washed with water and Et_2O, to give the product as an off-white solid (2620 mg, 93%). R_f = 0.60 (DCM/MeOH 9:1); Mp: 222 °C dec; ^{1}H NMR (400 MHz, $CDCl_3$) δ = 8.35 (t, J = 1.2 Hz, 2H), 7.64 (s, 2H), 7.62 (t, J = 1.5 Hz, 2H), 7.24 (t, J = 1.2 Hz, 2H) ppm; ^{13}C NMR (101 MHz, $CDCl_3$) δ = 148.49, 135.19, 131.64, 118.82, 116.18, 109.27 ppm; IR $\tilde{\nu}_{max}$ = 3108 (w, br), 1582 (s), 1563 (m), 1517 (w), 1476 (s), 1434 (s), 1344 (w), 1319 (w), 1288 (w), 1234 (m). 1102 (m), 1055 (m), 1010 (s), 903 (w), 828 (m), 774 (w), 729 (m), 650 (m), 616 (w) cm^{-1}; HRMS-ESI: m/z $[M+H]^+$ calcd for $C_{11}H_9N_5I$: 337.9903; found 337.9901; Anal. Calcd for $C_{11}H_8IN_5$ C, 39.19; H, 2.39; N, 20.77. Found: C 39.11, H 2.41, N 20.75.

2,6-Di(imidazol-1-yl)-4-(phenylethynyl)pyridine (7). To a dry, degassed solution of 2,6-di(imidazole-1-yl)-4-iodopyridine (250 mg, 1.04 mmol), $Pd(PPh_3)_2Cl_2$ (37 mg, 0.053 mmol) and CuI (16 mg, 0.084 mmol) in DIPA (1 mL) and DMF (17 mL) was dropwise added phenylacetylene (0.12 mL, 1.0 mmol), over 1 min. The resulting black solution was stirred at rt for 18 h. To the reaction was added NH_4Cl (saturated aq., 70 mL), giving precipitate. The resulting suspension was extracted with chloroform (3 x 70 mL). The combined organic phases were washed with brine (140 mL), dried over $MgSO_4$, and evaporated *in vacuo*. The residue was further purified by silica gel chromatography (2 x 16 cm, DCM/MeOH 94:6) to give the product as an off-white solid (209 mg, 67%). R_f = 0.28 (DCM/MeOH 94:6); Mp: 193 °C dec; ^{1}H NMR (400 MHz, $CDCl_3$) δ = 8.39 (t, J = 1.1 Hz, 2H, Im-*H*2), 7.67 (t, J = 1.4 Hz, 2H, Im-*H*5), 7.63 – 7.58 (m, 2H, Ph-*H*2), 7.49 – 7.39 (m, 3H, Ph-*H*3 & Ph-*H*4), 7.36 (s, 2H, Py-*H*3), 7.25 (t, J = 1.2 Hz, 2H, Im-*H*4) ppm; ^{13}C NMR (101 MHz, $CDCl_3$) δ = 148.73, 137.70, 135.19, 132.25, 131.42, 130.17, 128.84, 121.25, 116.26, 111.61, 96.83, 85.58 ppm; IR $\tilde{\nu}_{max}$ = 3093 (w, br), 2213 (w), 1606 (s), 1555 (s),1520 (w), 1466 (s), 1439 (s), 1329 (w), 1283 (s), 1261 (m), 1230 (w), 1161

(w),1056 (s), 1013 (s), 894 (w), 866 (w), 839 (w), 781 (w), 747 (m), 736 (m), 684 (m), 651 (m), 541 (w), 526 (w), 463 (w) cm^{-1}; HRMS-ESI: m/z $[M + H]^+$ calcd for $C_{19}H_{14}N_5$: 312.1249; found 312.1247; Anal. Calcd for $C_{19}H_{13}N_5$: C 73.30, H 4.21, N 22.49. Found: C 73.24, H 4.19, N 22.44.

4-((4-Bromophenyl)ethynyl)-2,6-di(imidazol-1-yl)pyridine (8). To a dry, degassed solution of 2,6-di(imidazol-1-yl)-4-iodopyridine (400 mg, 1.19 mmol), $Pd(PPh_3)_2Cl_2$ (85 mg, 0.12 mmol) and CuI (26 mg, 0.14 mmol) in DIPA (2 mL) and DMF (30 mL) was dropwise added a dry, degassed solution of 1-bromo-4-ethynylbenzene (229 mg, 1.26 mmol) in dry DMF (20 mL), over 8 min. The resulting yellow solution was stirred at rt for 16 h. To the reaction was added NH_4Cl (saturated aq., 100 mL), giving precipitate. The resulting suspension was extracted with chloroform (3 x 100 mL). The combined organic phases were washed with brine (200 mL), dried over Na_2SO_4 and evaporated *in vacuo*. The residue was further purified by silica gel chromatography (3 x 17 cm, DCM/MeOH 94:6) to give the product as an off-white solid (410 mg, 89%). R_f = 0.34 (DCM/MeOH 94:6); Mp: 206 °C dec; ^{1}H NMR (400 MHz, $CDCl_3$) δ = 8.39 (s, 2H, Im-*H*2), 7.67 (t, *J* = 1.4 Hz, 2H, Im-*H*5), 7.59 – 7.55 (m, 2H, Ph-*H*2/ Ph-*H*3), 7.49 – 7.44 (m, 2H, Ph-*H*2/ Ph-*H*3), 7.35 (s, 2H, Py-*H*3), 7.25 (s, 2H, Im-*H*4) ppm; ^{13}C NMR (101 MHz, $CDCl_3$) δ = 148.78, 137.32, 135.19, 133.57, 132.22, 131.48, 124.79, 120.16, 116.25, 111.53, 95.56, 86.55 ppm; IR $\tilde{\nu}_{max}$ = 3102 (w, br), 2215 (w), 1607 (s), 1585 (w), 1555 (s), 1519 (w), 1481 (s), 1439 (s), 1394 (w), 1328 (w), 1283 (s), 1264 (w), 1233 (w), 1103 (w), 1057 (m), 1011 (s), 895 (w), 823 (m), 781 (w), 751 (m), 651 (m), 543 (w), 523 (w) cm^{-1}; HRMS-ESI: m/z $[m + H]^+$ calcd for $C_{19}H_{13}N_5Br$: 390.0354; found 390.0358; Anal. Calcd for $C_{19}H_{12}BrN_5 \cdot 0.2\ H_2O$: C 57.86, H 3.21, N 17.71. Found: C 57.76, H 3.34, N 17.82.

4-(2,6-Di(imidazol-1-yl)pyridine-4-yl)ethynyl)-*N*,*N*-dimethylaniline (9). To a dry, degassed solution of 2,6-di(imidazole-1-yl)-4-iodopyridine (300 mg, 0.890 mmol), $Pd(PPh_3)_2Cl_2$ (62.3 mg, 0.0888 mmol) and CuI (18 mg, 0.095 mmol) in DIPA (1 mL) and DMF (15 mL) was dropwise added a dry, degassed solution of 4-ethynyl-*N*,*N*-dimethylaniline (193 mg, 1.33 mmol) in DMF (10 mL), over 7 min. The resulting solution was stirred at rt for 17 h. To the reaction was added NH_4Cl (saturated aq., 70 mL), giving yellow precipitate. The resulting suspension was extracted with chloroform (3 x 70 mL). The combined organic phases were washed with brine (140 mL), dried over $MgSO_4$, and evaporated *in vacuo*. The residue was further purified by silica gel chromatography (3 x 16 cm, DCM/MeOH 94:6) to give the product as a yellow solid (263 mg, 83%). R_f = 0.36 (DCM/MeOH 94:6)); Mp: 209 °C dec; ^{1}H NMR (400 MHz, $CDCl_3$) δ = 8.38 (t, *J* = 1.2 Hz, 2H, Im-*H*2), 7.67 (t, *J* = 1.4 Hz, 2H, Im-*H*5), 7.51 – 7.43 (m, 2H, Ani-*H*3), 7.29 (s, 2H, Py-*H*3), 7.24 (dd, *J* = 1.3, 0.9 Hz, 2H, Im-*H*4), 6.72 – 6.65 (m, 2H, Ani-*H*2), 3.04 (s, 6H, Ani-N-CH_3) ppm; ^{13}C NMR (101 MHz, $CDCl_3$) δ = 151.28, 148.61, 138.67, 135.19, 133.76, 131.25, 116.29, 111.83, 111.09, 107.28, 99.64, 84.83, 40.21 ppm; IR $\tilde{\nu}_{max}$ = 3107 (w, br), 2203 (m), 1594 (s), 1551 (m), 1527 (m), 1479 (s), 1366 (m), 1284 (w), 1231 (w), 1196 (w), 1142 (m), 1101 (w), 1056 (w), 1014 (w), 945 (w), 896 (w), 816 (w), 782 (w), 750 (w), 655 (w), 520 (w) cm^{-1}; HRMS-ESI: m/z $[M+H]^+$ calcd for $C_{21}H_{19}N_6$: 355.1671; found 355.1672; Anal. Calcd for $C_{21}H_{18}N_6$: C71.17, H 5.12, N 23.71. Found: C 70.96, H 5.19, N 23.59.

1,1'-(4-(Phenylethynyl)pyridine-2,6-diyl)bis(3-methylimidazolium) bis(hexafluorophosphate) (10). To a suspension of 2,6-di(imidazol-1-yl)-4-(phenylethynyl)pyridine (189 mg, 0.607 mmol) in DMF (4 mL) was dropwise added MeI (0.2 mL, 3 mmol) over 2 min. The reaction vessel was sealed with a screwcap and the resulting mixture was heated to 130 °C and stirred at that temperature for 1 h. The resulting brown mixture was allowed to cool to rt and diluted with methanol (20 mL), and hydrochloric acid (concd. aq., 0.5 mL) was added dropwise. To the resulting solution was added a solution of ammonium hexafluorophospate (866 mg, 5.31 mmol) in water (5 mL), followed by slow addition of water (15 mL). The resulting precipitate was collected on a glass filter (#3), consecutively washed with water (4 x 10 mL) and Et_2O (3 x 10 mL), and dissolved in acetonitrile and passed through the filter. The resulting filtrate was evaporated *in vacuo* giving the product as an off-white solid (362 mg, 94%). R_f = 0.36 (40:9:1, MeCN:H_2O:KNO_3 (sat. aq.)); Mp: 212 °C dec; ^{1}H NMR (400 MHz, CD_3CN) δ = 9.46 (dq, *J* = 1.7, 0.8 Hz, 2H, Im-*H*2), 8.17 (t, *J* = 2.0 Hz, 2H, Im-*H*5), 8.02 (s, 2H, Py-*H*3), 7.71 – 7.67 (m, 2H, Ph-*H*2), 7.63 (t, *J* = 1.9 Hz, 2H, Im-*H*4), 7.60 – 7.49 (m, 3H, Ph-*H*3 & Ph-*H*4), 4.02 (s, 6H, Im-CH_3) ppm; ^{13}C NMR (101 MHz, CD_3CN) δ = 146.87, 140.11, 136.39, 133.16, 131.73, 130.04, 126.30, 121.44, 120.34, 117.38, 99.71, 85.36, 37.78 ppm; IR $\tilde{\nu}_{max}$ = 3158 (w, br), 2228 (w), 1709 (w), 1624 (s), 1583 (w), 1537 (w), 1446 (w), 1224 (m), 1133 (w), 1087 (w), 1019 (w), 836 (vs),

766 (s), 694 (w), 643 (w), 621 (w), 558 (s) cm^{-1}; HRMS-ESI: m/z [M + PF_6]$^+$ calcd for $C_{21}H_{19}F_6N_5P$: 468.1282; found 486.1286; Anal. Calcd for $C_{21}H_{19}F_{12}N_5P_2$: C 39.95, H 3.03, N 11.09. Found: C 39.79, H 2.99, N 11.02.

1,1'-(4-((4-Bromophenyl)ethynyl)pyridine-2,6-diyl)bis(3-metylimidazolium)bis(hexafluorophosphate) (11). A suspension of 4-((4-Bromophenyl)ethynyl)-2,6-di(imidazol-1-yl)pyridine (74 mg, 0.19 mmol) in DCM (7 mL) was cooled to 0 °C. To the suspension was dropwise added MeOTf (0.08 mL, 0.7 mmol). The reaction vessel was sealed with a screwcap and the resulting mixture was allowed to warm to rt and stirred at that temperature for 16 h. The resulting mixture was evaporated *in vacuo* and the residue was dissolved in MeCN (5 mL). To the resulting solution was added hydrochloric acid (concd. aq., 0.1 mL) followed by KPF_6 (saturated aq., 5 mL) and water (4 mL), forming a white precipitate. The precipitate was collected on a glass filter (#4), consecutively washed with water (3 x 5 mL), and Et_2O (3 x 5 mL) to give the product as an off-white solid (125 mg, 93%). R_f = 0.41 (MeCN/H_2O/KNO_3 (saturated aq.) 40:9:1); Mp: 171 °C dec; ^{1}H NMR (400 MHz, CD_3CN) δ = 9.40 (app. td, *J* = 1.7, 0.8 Hz, 2H, Im-*H*2), 8.15 (t, *J* = 2.0 Hz, 2H, Im-*H*5), 8.01 (s, 2H, Py-*H*3), 7.73 – 7.67 (m, 2H, Ph-*H*2/Ph-*H*3), 7.63 (t, *J* = 1.9 Hz, 2H, Im-*H*4), 7.60 – 7.55 (m, 2H, Ph-*H*2/Ph-*H*3), 4.00 (s, 6H, Im-C*H*$_3$) ppm; ^{13}C NMR (101 MHz, CD_3CN) δ = 146.86, 139.76, 136.32, 134.77, 133.23, 126.30, 125.65, 120.63, 120.32, 117.36, 98.33, 86.28, 37.76 ppm; IR $\tilde{\nu}_{max}$ = 3166 (w, br), 2223 (w), 1703 (w), 1624 (s), 1582 (w), 1537 (m), 1492 (w), 1446 (m), 1225 (m), 1087 (w), 1070 (w), 1012 (w), 834 (vs), 753 (w), 643 (w), 620 (w), 558 (s) cm^{-1}; ESI-HRMS: m/z [M + PF_6]$^+$ calcd for $C_{21}H_{18}N_5BrPF_6$: 564.0387; found 564.0388; Anal. Calcd for $C_{21}H_{18}BrF_{12}N_5P_2$: C 35.51, H 2.55, N 9.86. Found: C 35.27, H 2.56, N 9.83.

1,1'-(4-((4-(dimethylamino)phenyl)ethynyl)pyridine-2,6-diyl)bis(3-methylimidazolium) bis(hexafluorophosphate) (12). To a suspension of 4-(2,6-Di(imidazol-1-yl)pyridine-4-yl)ethynyl)-*N*,*N*-dimethylaniline (252 mg, 0.711 mmol) in MeCN (13 mL) was dropwise added MeI (0.4 mL, 6 mmol). The reaction vessel was sealed with a screwcap and the resulting mixture heated to 85 °C and stirred at that temperature for 17 h. The resulting reaction mixture was allowed to cool to rt and diluted with methanol (50 mL) and hydrochloric acid (concd. aq., 0.3 mL). To the resulting solution was slowly added KPF_6 (sat. aq., 47 mL). The resulting precipitate was collected on a glass filter (#3) and was further purified by silica gel chromatography (4 x 16 cm, MeCN/H_2O/KNO_3 (saturated aq.) 40:9:1). The desired fractions were evaporated *in vacuo* and to the remaining aqueous suspension was added methanol (100 mL) and hydrochloric acid (concd. aq. 0.5 mL), giving an orange solution. To the solution was added ammonium hexafluorophospate (1.144 g, 7.018 mmol) in water (50 mL) in two portions and the resulting precipitate was filtered off on a glass filter (#4) and carefully washed with water (2 x 10 mL) followed by Et_2O (2 x 50 mL), to give the product after drying at 60 °C *in vacuo* as a yellow powder (330 mg, 69%). R_f = 0.43 (MeCN/H_2O/KNO_3 (saturated aq.) 40:9:1); Mp: 184 °C dec; ^{1}H NMR (400 MHz, CD_3CN) δ = 9.41 (d, *J* = 0.9 Hz, 2H, Im-*H*2), 8.15 (t, *J* = 2.0 Hz, 2H, Im-*H*5), 7.90 (s, 2H, Py-*H*3), 7.62 (t, *J* = 2.0 Hz, 2H, Im-*H*4), 7.53 – 7.46 (m, 2H, Ani-*H*2), 6.82 – 6.74 (m, 2H, Ani-*H*3), 4.01 (s, 6H, Im-C*H*$_3$), 3.03 (s, 6H, Ani-N-C*H*$_3$) ppm; ^{13}C NMR (101 MHz, CD_3CN) δ = 152.82, 146.75, 141.11, 136.25, 134.77, 126.19, 120.28, 116.27, 112.76, 106.69, 103.66, 85.21, 40.19, 37.73 ppm; IR $\tilde{\nu}_{max}$ = 2948 (w, br), 2181 (w), 1696 (w), 1622 (w), 1595 (s), 1530 (s), 1479 (w), 1402 (w), 1370 (w), 1265 (w), 1149 (w), 1115 (m), 1056 (w), 1006 (m), 834 (vs), 738 (w), 687 (w), 557 (m), 477 (w), 428 (w) cm^{-1}; ESI-HRMS: m/z [m + PF_6]$^+$ calcd for $C_{23}H_{24}F_6N_6P$: 529.1704; found 529.1711; Anal. Calcd for $C_{23}H_{24}F_{12}N_6P_2$: C 40.96, H 3.59, N 12.46. Found: C 40.74, H 3.54, N 12.42.

Bis(1,1´-(4-((phenyl)ethynyl)pyridine-2,6-diyl)bis(3-methylimidazolylidene))iron bis(hexafluorophosphate) (2). 1,1´-(4-((Phenyl)ethynyl)pyridine-2,6-diyl)bis(3-methylimidazolium) bis(hexafluorophosphate) (103 mg, 0.163 mmol) was heated to 60 °C *in vacuo*. The solid was allowed to cool to rt and suspended in dry, degassed THF (6 mL), and the resulting suspension was cooled to -78 °C. To the resulting suspension was added KO*t*-Bu (1 M in THF, 0.4 mL, 0.4 mmol) and the resulting yellow-brown solution was stirred for 30 min. To the resulting solution was dropwise added a solution of $FeBr_2$ (22 mg, 0.10 mmol) in THF (4 mL). The resulting dark red mixture was allowed to warm to rt and stirred at that temperature for 1 h. The resulting mixture was evaporated *in vacuo* and to the residue was added methanol (30 mL) and hydrochloric acid (concd. aq., 0.6 mL). The resulting mixture was filtered through a glass filter (#3). To the resulting filtrate was added a solution of

ammonium hexafluorophospate (805 mg, 4.94 mmol) in water (5 mL), followed by slow addition of water (20 mL). The resulting precipitate was collected on a glass filter (#4) and the washed with water (4 x 10 mL). The material was further purified twice by size-exclusion chromatography (3 x 140 cm, MeCN/PhMe 1:1) giving the target complex as a red solid (12 mg, 14%). R_f = 0.48 (MeCN/H_2O/KNO_3 (saturated aq.) 180:19:1); Mp: 230 °C dec; ^{1}H NMR (400 MHz, CD_3CN) δ = 8.02 (d, *J* = 2.2 Hz, 4H, Im-*H*5), 7.90 (s, 4H, Py-*H*3), 7.72 – 7.67 (m, 4H, Ph-*H*2), 7.56 – 7.50 (m, 6H, Ph-*H*3 & Ph-*H*4), 7.03 (d, *J* = 2.2 Hz, 4H, Im-*H*4), 2.58 (s, 12H, Im-C*H*3) ppm; ^{13}C NMR (101 MHz, CD_3CN) δ = 200.44, 154.74, 132.96, 132.76, 131.21, 130.02, 127.75, 122.10, 117.41, 108.03, 97.35, 87.42, 35.67 ppm; IR $\tilde{\nu}_{max}$ = 3145 (w, br), 2216 (w),1707 (w), 1621 (s), 1575 (w), 1534 (s), 1482 (s), 1404 (m), 1347 (m), 1266 (s), 1122 (w), 1088 (w), 1007 (w), 836 (vs), 763 (w), 740 (w), 689 (s), 557 (s), 463 (m) cm^{-1}; HRMS-ESI: m/z [M + PF_6]$^+$ calcd for $C_{42}H_{34}F_6FeN_{10}P$: 879.1935; found 879.1938; Anal. Calcd for $C_{42}H_{38}F_{12}FeN_{10}P_2$: C 49.24, H 3.34, N 13.67. Found: C 49.32, H 3.31, N 13.64.

Bis(1,1´-(4-((4-bromophenyl)ethynyl)pyridine-2,6-diyl)bis(3-methylimidazolylidene))iron bis(hexafluorophosphate) (3). 1,1´-(4-((4-Bromophenyl)ethynyl)pyridine-2,6-diyl)bis(3-methylimidazolium) bis(hexafluorophosphate) (251 mg, 0.353 mmol) was heated to 60 °C *in vacuo*. The solid was allowed to cool to rt and suspended in dry, degassed THF (15 mL) and the resulting suspension was cooled to -40 °C. Separately, to a solution of DIPA (0.1 mL, 0.7 mmol) in dry, degassed THF (11 mL), cooled to -78 °C, was added a solution of *n*-BuLi (2.5 M in hexanes, 0.3 mL, 0.8 mmol) and the resulting solution was stirred for 1 h, at -78 °C. The resulting solution was cannulated slowly to the suspension of 1,1´-(4-((4-bromophenyl)ethynyl)pyridine-2,6-diyl)bis(3-methylimidazolium) bis(hexafluorophosphate). The resulting yellow-brown solution was stirred for at -40 °C for 30 min. To the resulting solution was dropwise added a solution of $FeBr_2$ (44 mg, 0.20 mmol) in dry, degassed THF (4 mL). The resulting dark red mixture was allowed to warm to rt and stirred for 1 h. The resulting mixture was evaporated *in vacuo* and to the residue was added methanol (70 mL) and hydrochloric acid (concd. aq., 1 mL). The resulting mixture was filtered through a glass filter (#3). To the resulting filtrate was added a solution of ammonium hexafluorophospate (2.272 g, 13.94 mmol) in water (10 mL), followed by slow addition of water (50 mL). The resulting precipitate was collected on a glass filter (#4) and the washed with water (4 x 25 mL). The material was further purified twice by size-exclusion chromatography (3 x 140 cm, MeCN/PhMe 1:1) giving the target complex as a red solid (67 mg, 32%). R_f = 0.50 (MeCN/H_2O/KNO_3 (saturated aq.) 180:19:1); Mp: 227 °C dec; ^{1}H NMR (400 MHz, CD_3CN) δ = 8.00 (d, *J* = 2.2 Hz, 4H, Im-*H*5), 7.89 (s, 4H, Py-*H*3), 7.72 – 7.68 (m, 4H, Ph-*H*2/Ph-*H*3), 7.61 – 7.56 (m, 4H, Ph-*H*2/ Ph-*H*3), 7.02 (d, *J* = 2.2 Hz, 4H, Im-*H*4), 2.56 (s, 12H, Im-C*H*3) ppm; ^{13}C NMR (101 MHz, CD_3CN) δ = 200.34, 154.74, 134.63, 133.22, 132.37, 127.82, 125.05, 121.29, 117.48, 108.04, 96.09, 88.43, 35.75 ppm; IR $\tilde{\nu}_{max}$ = 3144 (w, br), 2216 (w), 1707 (w), 1619 (s), 1575 (w), 1533 (s), 1480 (vs), 1404 (m), 1347 (m), 1265 (s), 1121 (w), 1089 (w), 1069 (w), 1009 (m), 837 (vs), 739 (w), 688 (m), 558 (s), 528 (w), 484 (w) cm^{-1}; HRMS-ESI: m/z [M + PF_6]$^+$ calcd for $C_{42}H_{32}Br_2F_6Fe N_{10}P$: 1035.0169; found 1035.0159; Anal. Calcd for $C_{42}H_{36}Br_2F_{12}FeN_{10}P_2$: C 42.67, H 2.73, N 11.85. Found: C 42.41, H 2.79, N 11.74.

Bis(1,1´-(4-((4-(*N,N*-dimethylammino)phenyl)ethynyl)pyridine-2,6-diyl)bis(3-methylimidazolylidene))iron bis(hexafluorophosphate) (4). 1,1´-(4-((4-(*N, N*-Dimethylammino)phenyl)ethynyl)pyridine-2,6-diyl)bis(3-methylimidazolium) bis(hexafluorophosphate) (177 mg, 0.262 mmol) was heated to 60 °C *in vacuo*. The solid was allowed to cool to rt and suspended in dry degassed THF (11 mL) and the resulting suspension was cooled to -78 °C. To the resulting suspension was added KO*t*-Bu (1 M in THF, 0.65 mL, 0.65 mmol) and the resulting orange solution was stirred for 30 min. To the resulting solution was dropwise added a solution of $FeBr_2$ (31 mg, 0.14 mmol) in THF (7 mL). The resulting dark red mixture was allowed to warm to rt and stirred for 1 h. The resulting mixture was evaporated *in vacuo* and to the residue was added methanol (35 mL) and hydrochloric acid (concd. aq., 0.6 mL). The resulting mixture was filtered through a glass filter (#3). To the resulting filtrate was added a solution of ammonium hexafluorophospate (1.067 g, 6.546 mmol) in water (5 mL), followed by slow addition of water (30 mL). The resulting precipitate was collected on a glass filter and the washed with water (4 x 20 mL). The material was further purified twice by size-exclusion chromatography (3 x 140 cm, MeCN/PhMe 1:1) giving the target complex as a red solid (35 mg, 24 %). R_f = 0.48 (MeCN/H_2O/KNO_3 (saturated aq.) 180:19:1); Mp: 118 °C dec; ^{1}H NMR (400 MHz, CD_3CN) δ = 8.00 (d, *J* = 2.3 Hz, 4H, Im-*H*5), 7.80 (s, 4H, Py-*H*3), 7.54 – 7.47 (m,

4H, Ani-*H*2), 7.02 (d, $J = 2.2$ Hz, 4H, Im-*H*4), 6.82 – 6.76 (m, 4H, Ani-*H*3), 3.04 (s, 12H, Ani-N-CH_3), 2.58 (s, 12H, Im-CH_3) ppm; ^{13}C NMR (101 MHz, CD_3CN) δ = 200.76, 154.59, 152.49, 134.39, 133.93, 127.57, 117.28, 112.85, 107.60, 107.31, 100.61, 86.69, 40.23, 35.62 ppm; IR $\tilde{\nu}_{max}$ = 2948 (w, br), 2181 (w), 1696 (w), 1622 (w), 1595 (s), 1530 (s), 1479 (w), 1402 (w), 1370 (w), 1265 (w), 1149 (w), 1115 (m), 1056 (w), 1006 (m), 834 (vs), 738 (w), 687 (w), 557 (m), 477 (w), 428 (w) cm^{-1}; HRMS-ESI: m/z $[M + PF_6]^+$ calcd for $C_{46}H_{44}F_6FeN_{12}P$: 965.2803; found 965.2805; Anal. Calcd for $C_{46}H_{44}F_{12}FeN_{12}P_2 \cdot 1.2\ H_2O$: C 48.75, H 4.14, N 14.83. Found: C 48.78, H 4.14, N 14.81.

NMR spectra

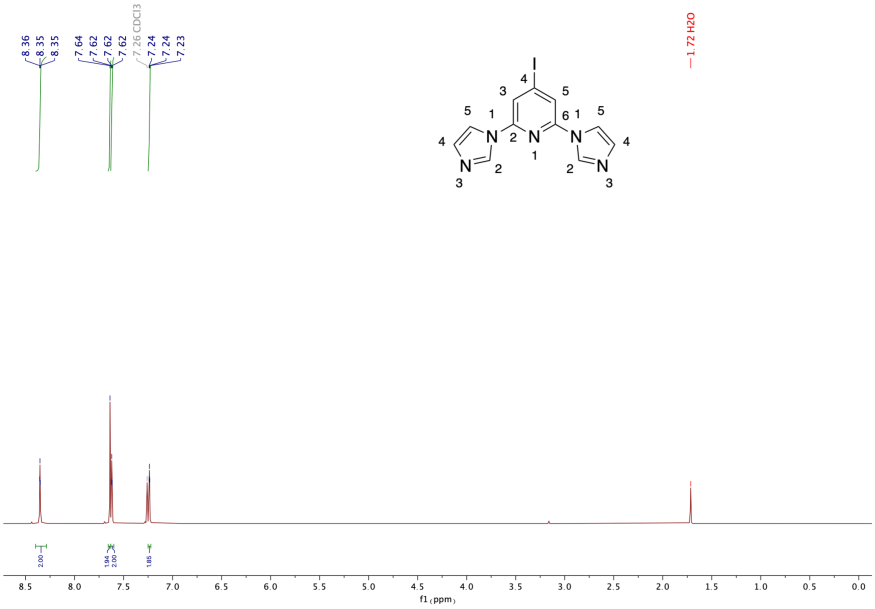

Figure S1: ^{1}H NMR ($CDCl_3$) spectrum of 2,6-di(imidazol-1-yl)-4-iodopyridine (**6**).

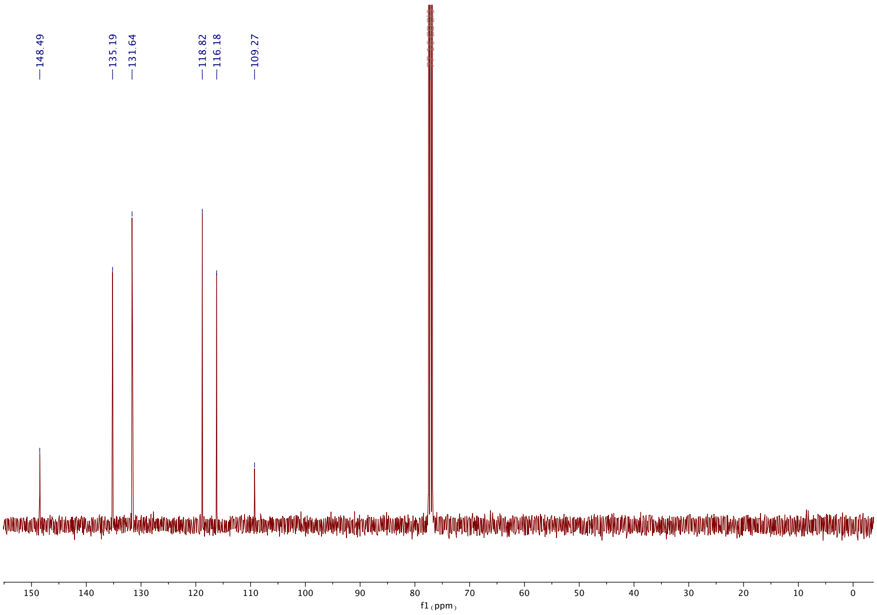

Figure S2: ^{13}C NMR ($CDCl_3$) spectrum of 2,6-di(imidazol-1-yl)-4-iodopyridine (**6**).

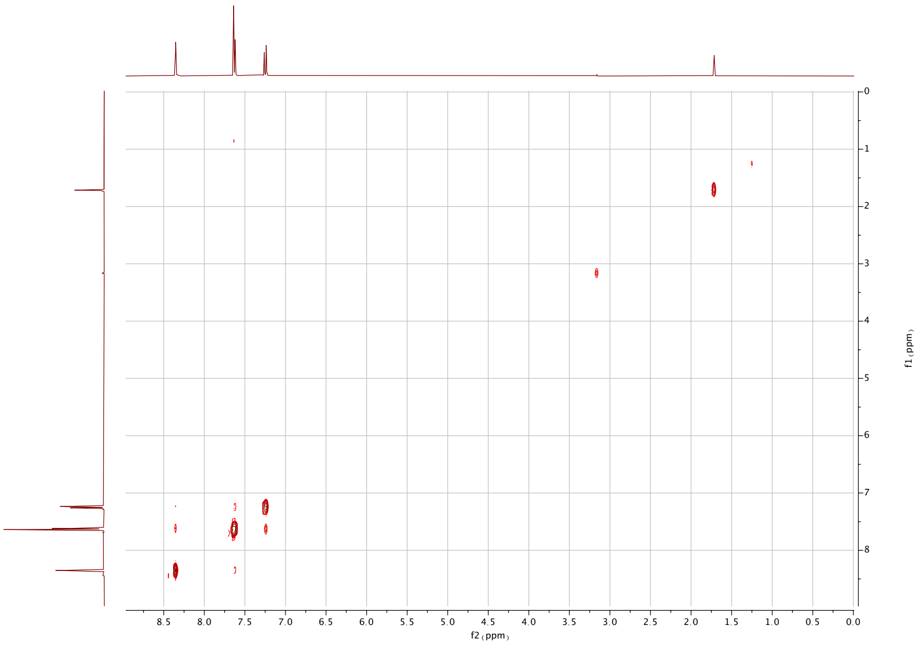

Figure S3: COSY NMR ($CDCl_3$) spectrum of 2,6-di(imidazol-1-yl)-4-iodopyridine (**6**).

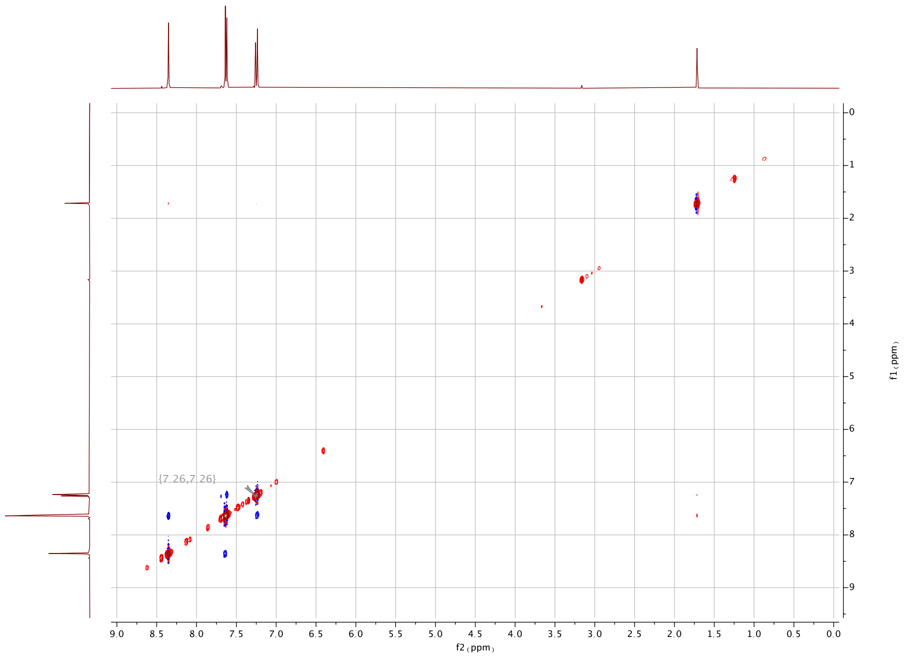

Figure S4: NOESY NMR ($CDCl_3$) spectrum of 2,6-di(imidazol-1-yl)-4-iodopyridine (**6**).

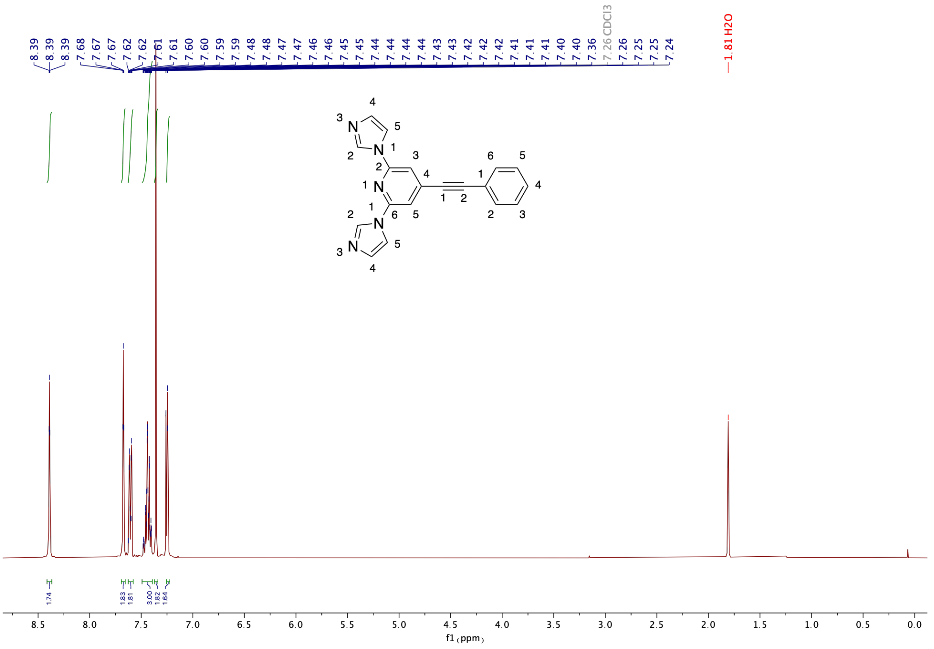

Figure S5: ^{1}H NMR ($CDCl_3$) spectrum of 2,6-di(imidazol-1-yl)-4-(phenylethynyl)pyridine (**7**).

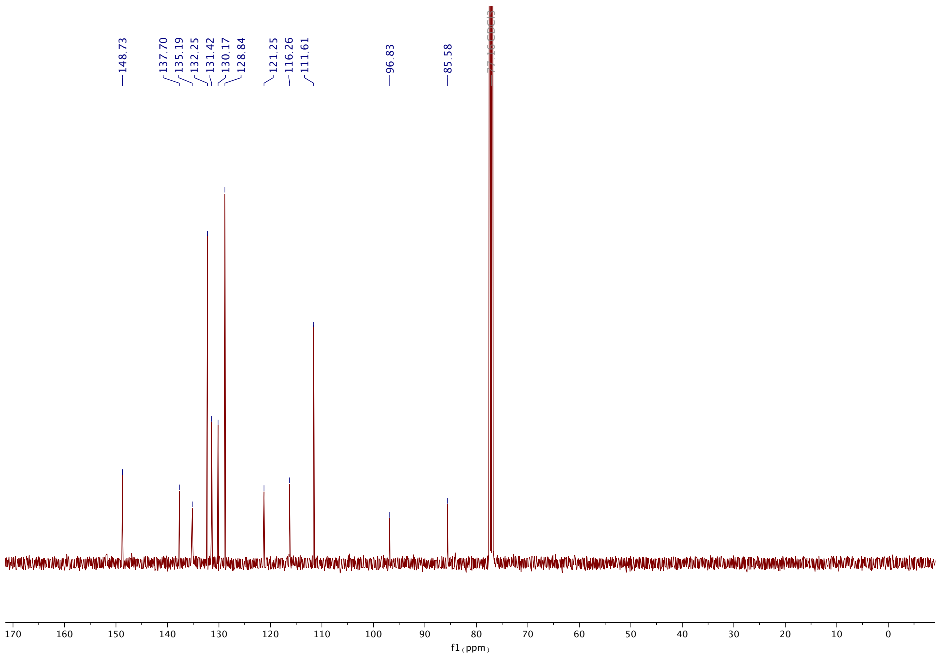

Figure S6: ^{13}C NMR ($CDCl_3$) spectrum of 2,6-di(imidazol-1-yl)-4-(phenylethynyl)pyridine (**7**).

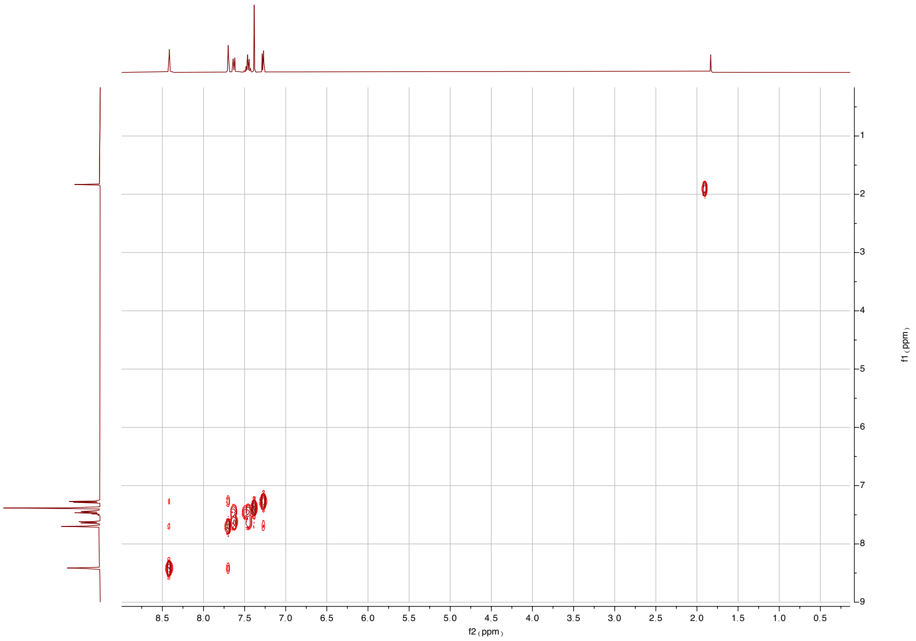

Figure S7: COSY NMR ($CDCl_3$) spectrum of 2,6-di(imidazol-1-yl)-4-(phenylethynyl)pyridine (**7**).

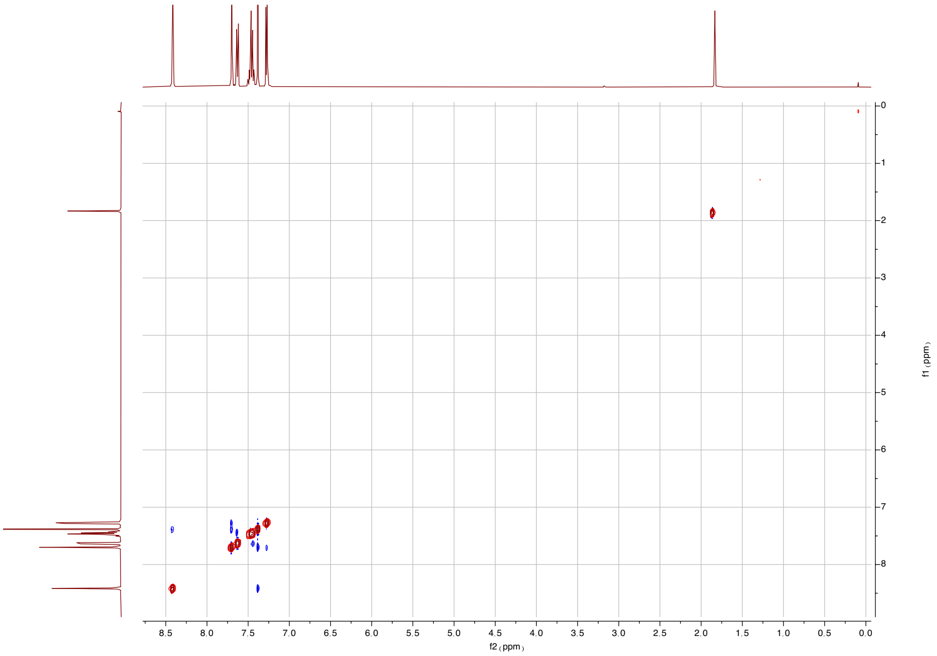

Figure S8: NOESY NMR ($CDCl_3$) spectrum of 2,6-di(imidazol-1-yl)-4-(phenylethynyl)pyridine (**7**).

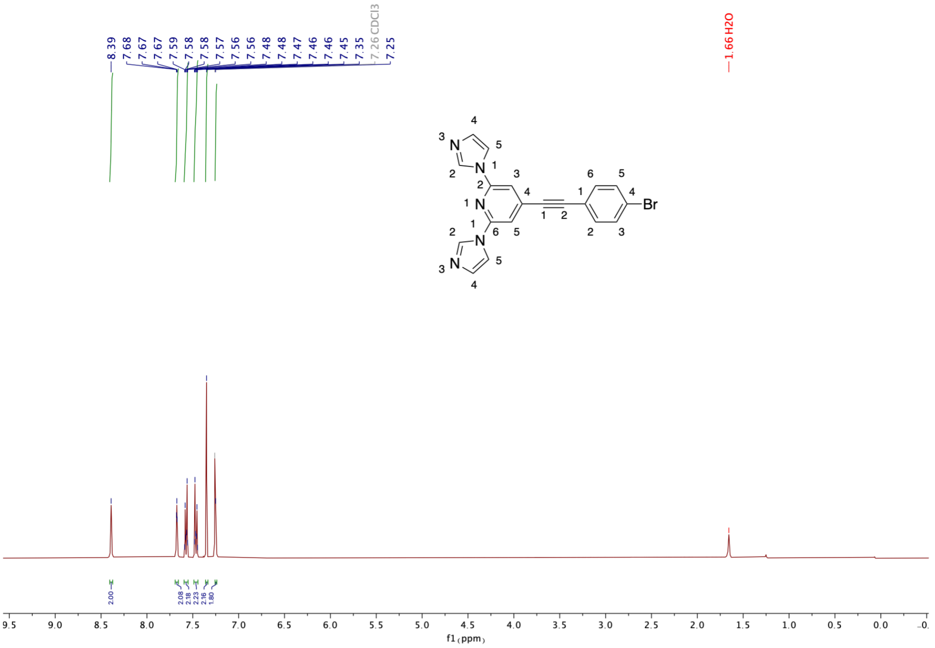

Figure S9: ^{1}H NMR ($CDCl_3$) spectrum of 4-((4-bromophenyl)ethynyl)-2,6-di(imidazol-1-yl)pyridine (**8**).

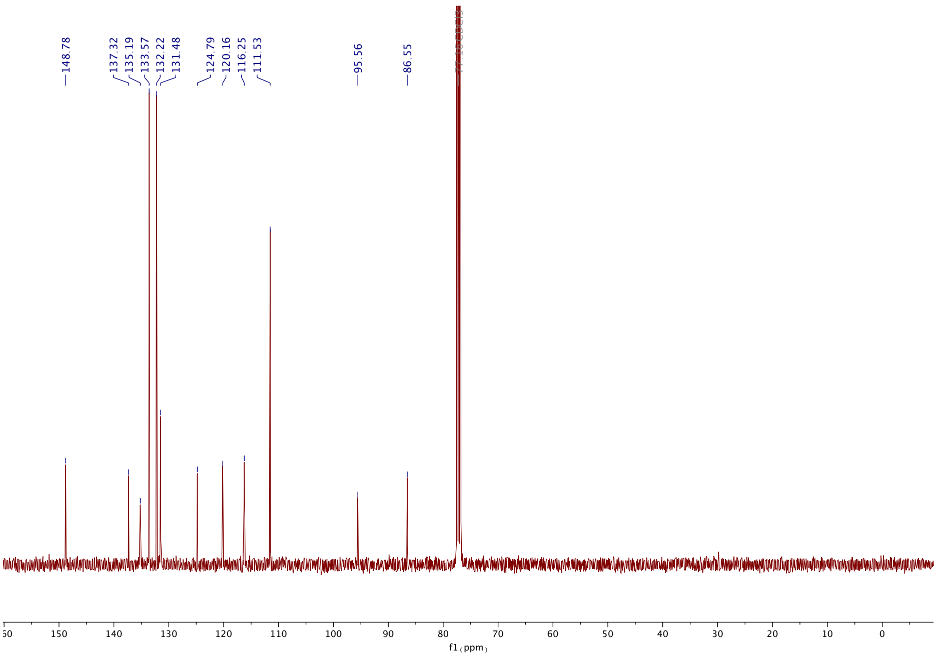

Figure S10: ^{13}C NMR ($CDCl_3$) spectrum of 4-((4-bromophenyl)ethynyl)-2,6-di(imidazol-1-yl)pyridine (**8**).

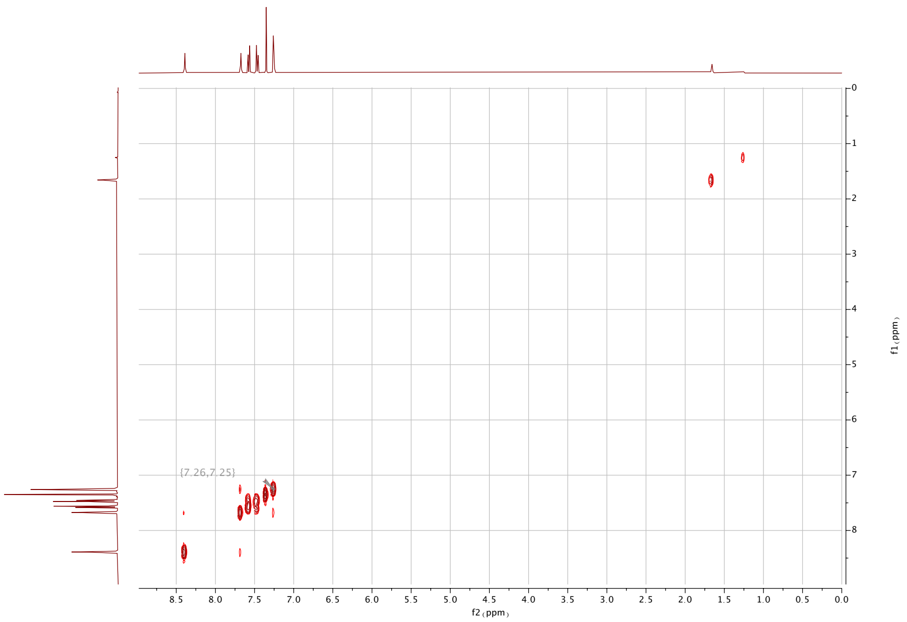

Figure S11: COSY NMR ($CDCl_3$) spectrum of 4-((4-bromophenyl)ethynyl)-2,6-di(imidazol-1-yl)pyridine (**8**).

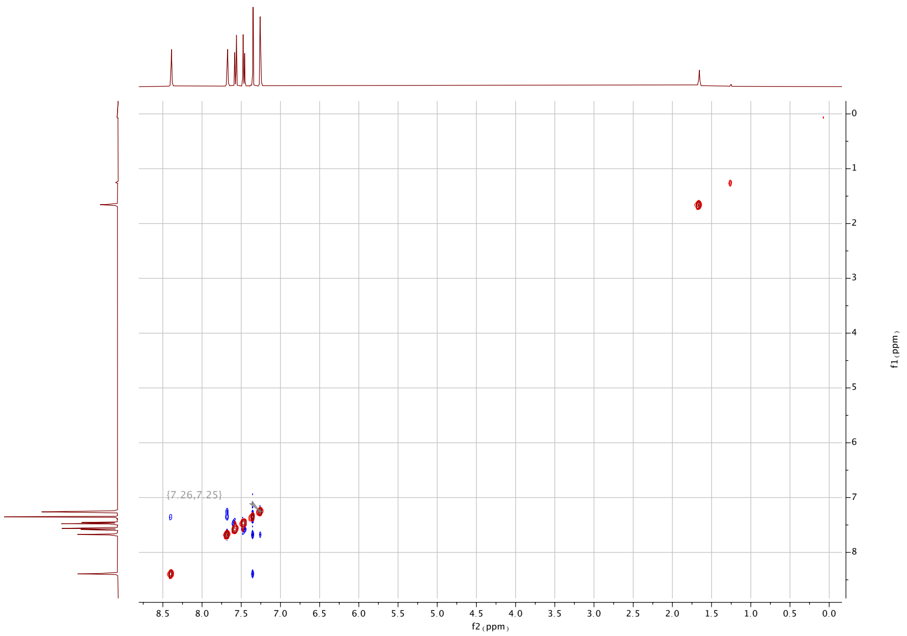

Figure S12: NOESY NMR ($CDCl_3$) spectrum of 4-((4-bromophenyl)ethynyl)-2,6-di(imidazol-1-yl)pyridine (**8**).

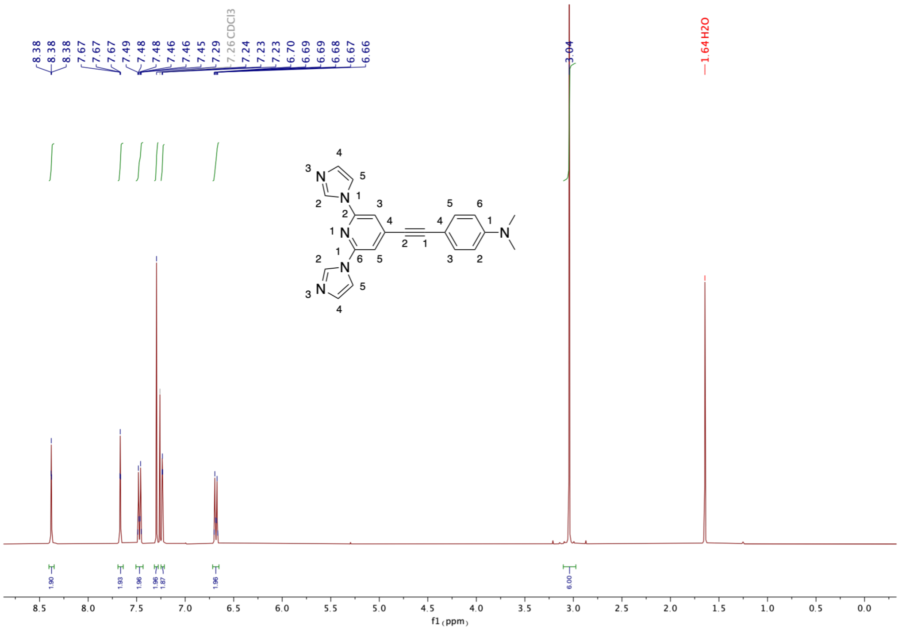

Figure S13: ^{1}H NMR ($CDCl_3$) spectrum of 4-(2,6-di(imidazol-1-yl)pyridine-4-yl)ethynyl)-*N*,*N*-dimethylaniline (**9**).

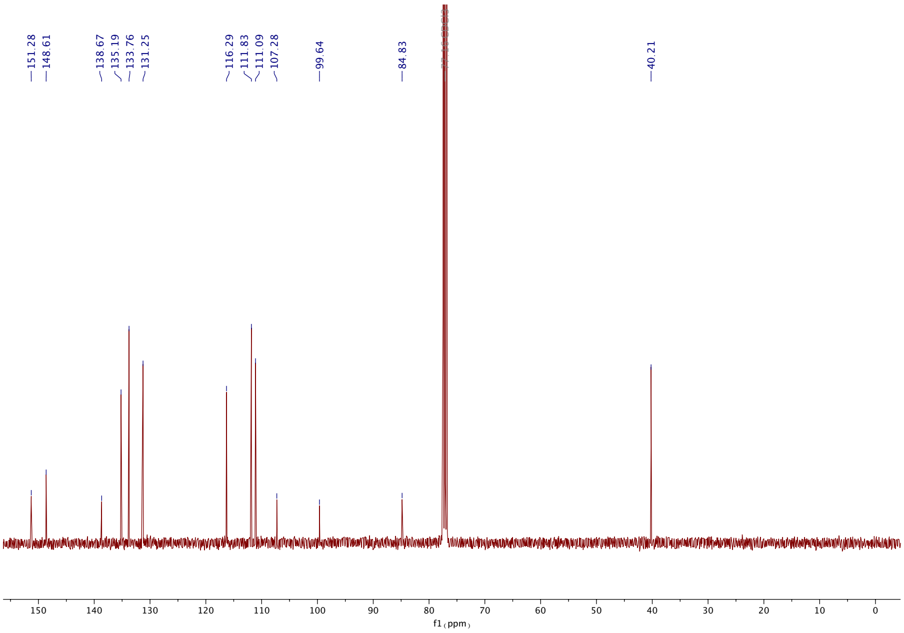

Figure S14: ^{13}C NMR ($CDCl_3$) spectrum of 4-(2,6-di(imidazol-1-yl)pyridine-4-yl)ethynyl)-*N*,*N*-dimethylaniline (**9**).

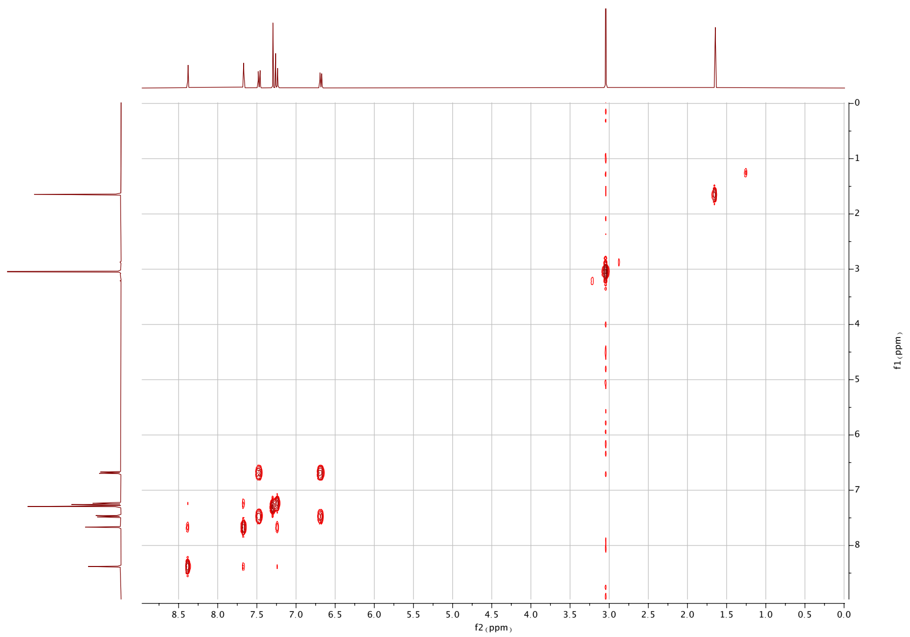

Figure S15: COSY NMR ($CDCl_3$) spectrum of 4-(2,6-di(imidazol-1-yl)pyridine-4-yl)ethynyl)-*N*,*N*-dimethylaniline (**9**).

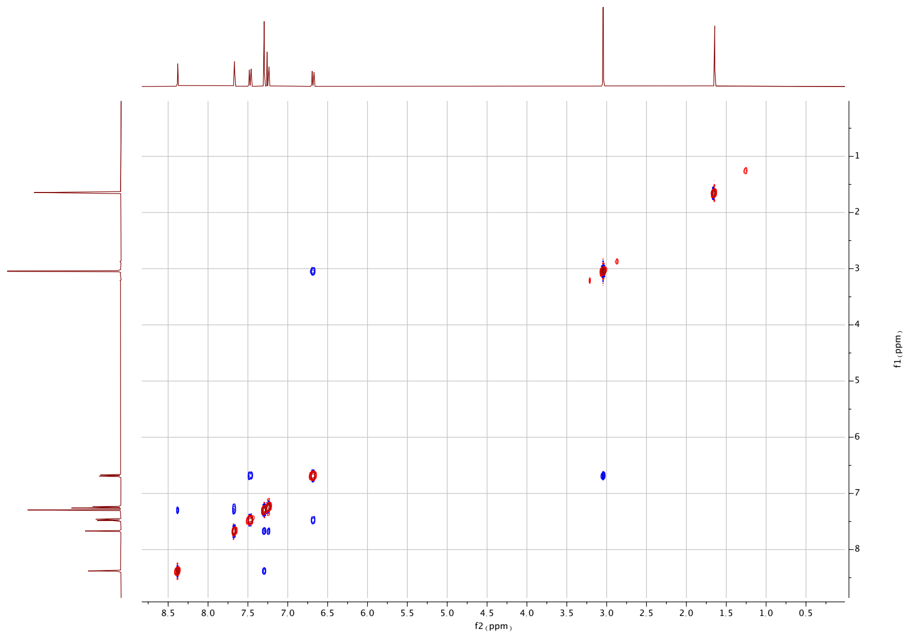

Figure S16: NOESY NMR ($CDCl_3$) spectrum of 4-(2,6-di(imidazol-1-yl)pyridine-4-yl)ethynyl)-*N*,*N*-dimethylaniline (**9**).

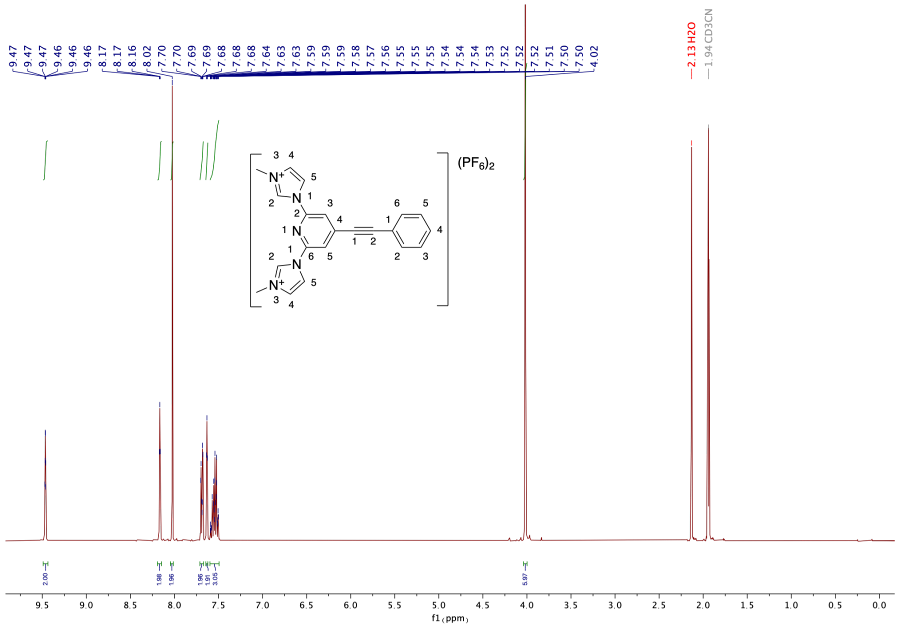

Figure S17: ^{1}H NMR (CD_3CN) spectrum of 1,1'-(4-(phenylethynyl)pyridine-2,6-diyl)bis(3-methylimidazolium) bis(hexafluorophosphate) (**10**).

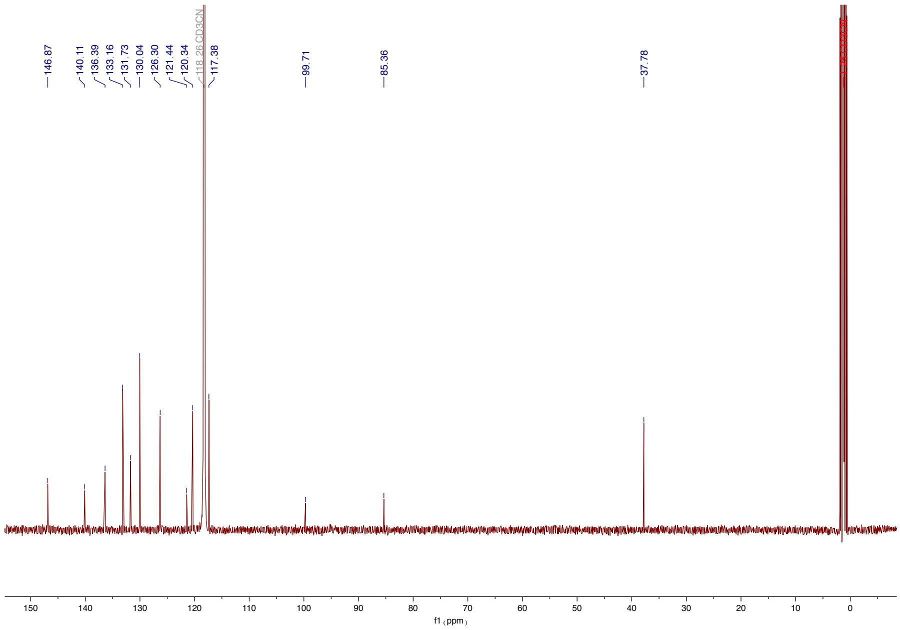

Figure S18: ^{13}C NMR (CD_3CN) spectrum of 1,1'-(4-(phenylethynyl)pyridine-2,6-diyl)bis(3-methylimidazolium) bis(hexafluorophosphate) (**10**).

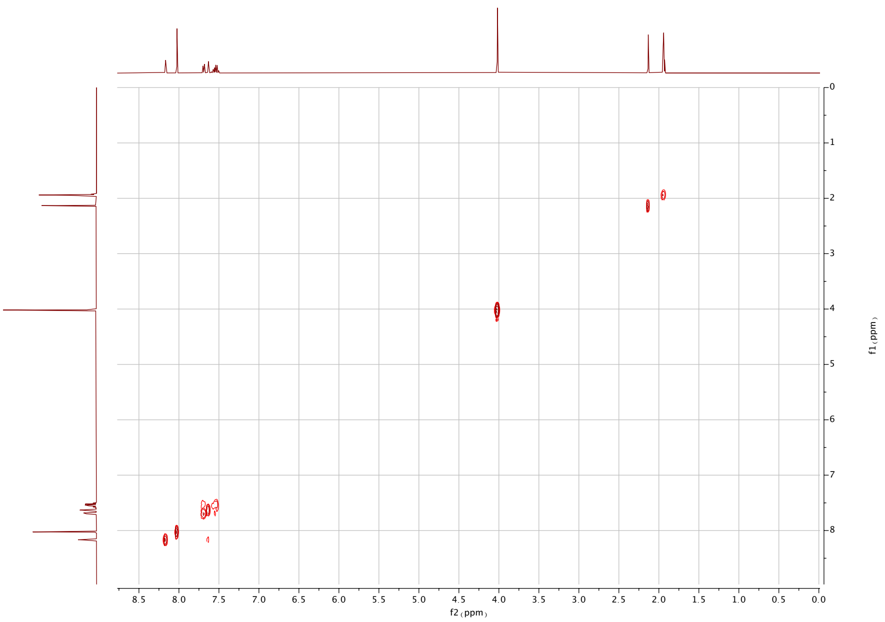

Figure S19: COSY NMR (CD_3CN) spectrum of 1,1'-(4-(phenylethynyl)pyridine-2,6-diyl)bis(3-methylimidazolium) bis(hexafluorophosphate) (**10**).

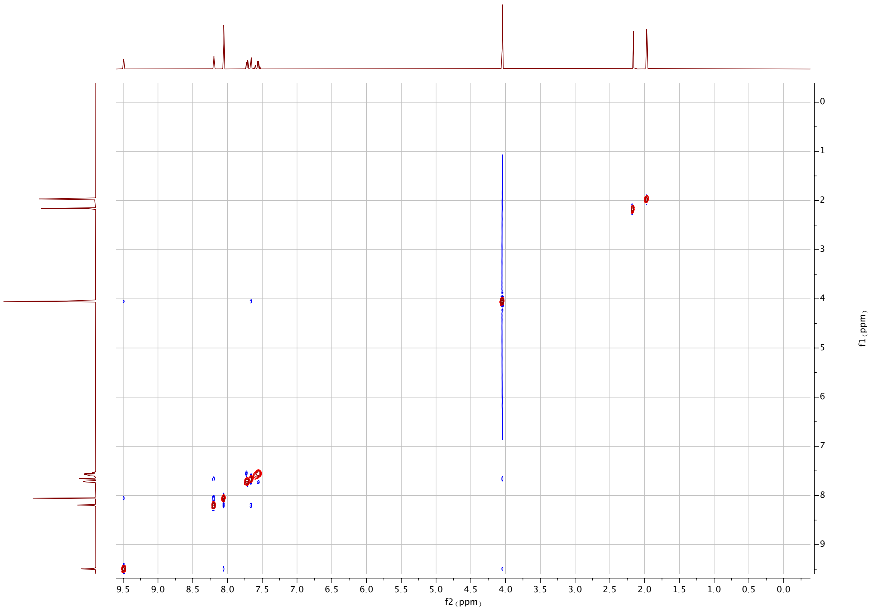

Figure S20: NOESY NMR (CD_3CN) spectrum of 1,1'-(4-(phenylethynyl)pyridine-2,6-diyl)bis(3-methylimidazolium) bis(hexafluorophosphate) (**10**).

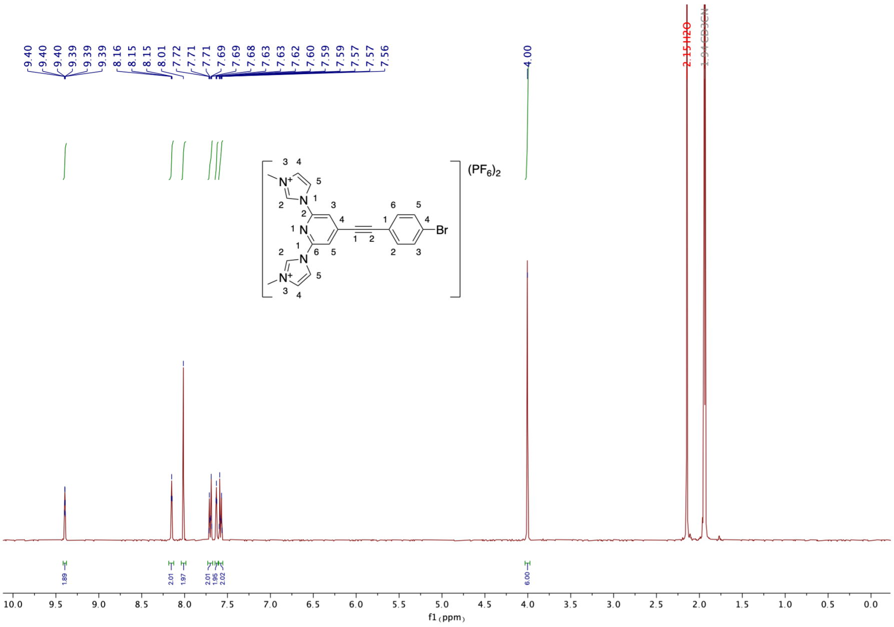

Figure S21: ^{1}H NMR (CD_3CN) spectrum of 1,1'-(4-((4-bromophenyl)ethynyl)pyridine-2,6-diyl)bis(3-metylimidazolium) bis(hexafluorophosphate) (**11**).

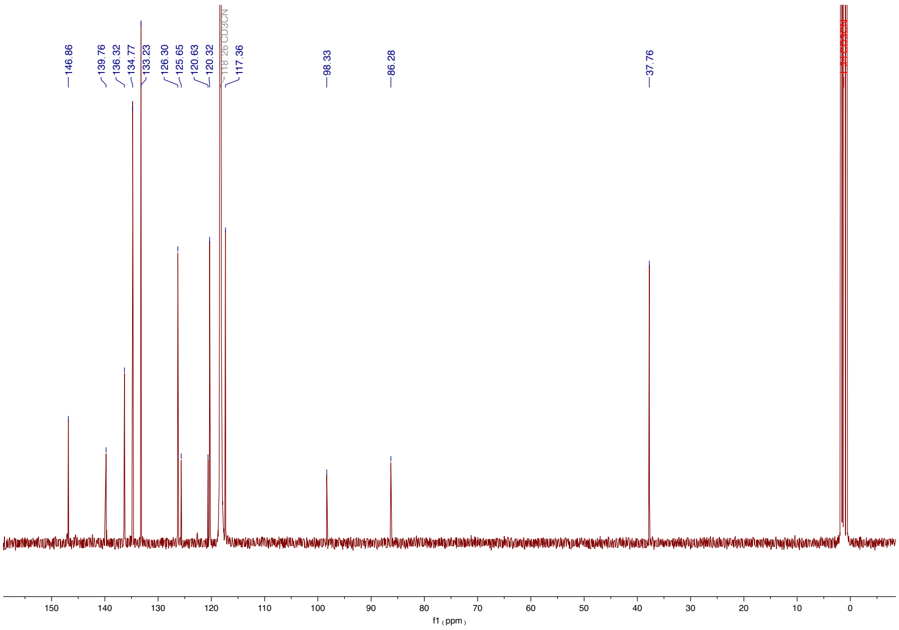

Figure S22: ^{13}C NMR (CD_3CN) spectrum of 1,1'-(4-((4-bromophenyl)ethynyl)pyridine-2,6-diyl)bis(3-metylimidazolium) bis(hexafluorophosphate) (**11**).

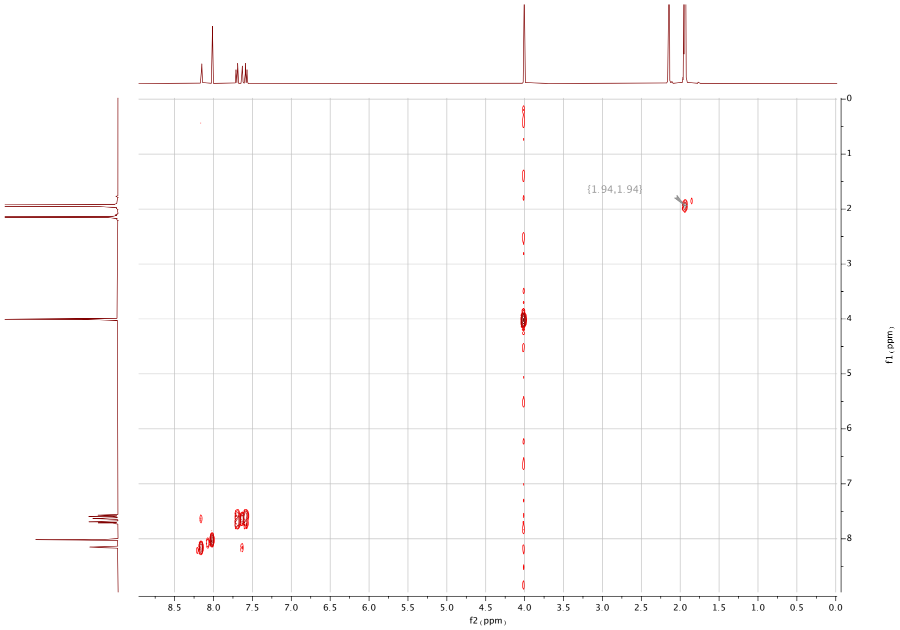

Figure S23: COSY NMR (CD_3CN) spectrum of 1,1’-(4-((4-bromophenyl)ethynyl)pyridine-2,6-diyl)bis(3-metylimidazolium) bis(hexafluorophosphate) (**11**).

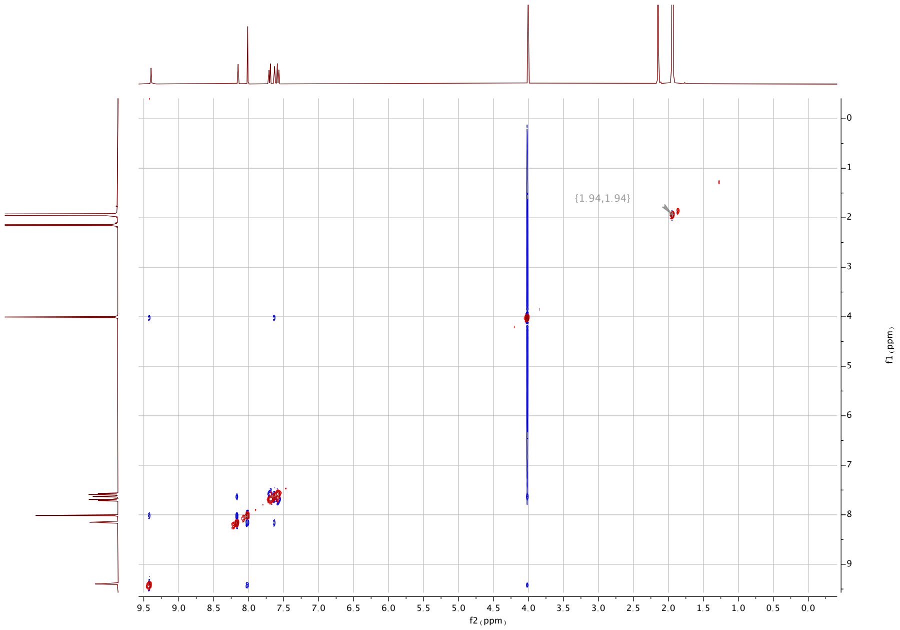

Figure S24: NOESY NMR (CD_3CN) spectrum of 1,1’-(4-((4-bromophenyl)ethynyl)pyridine-2,6-diyl)bis(3-metylimidazolium) bis(hexafluorophosphate) (**11**).

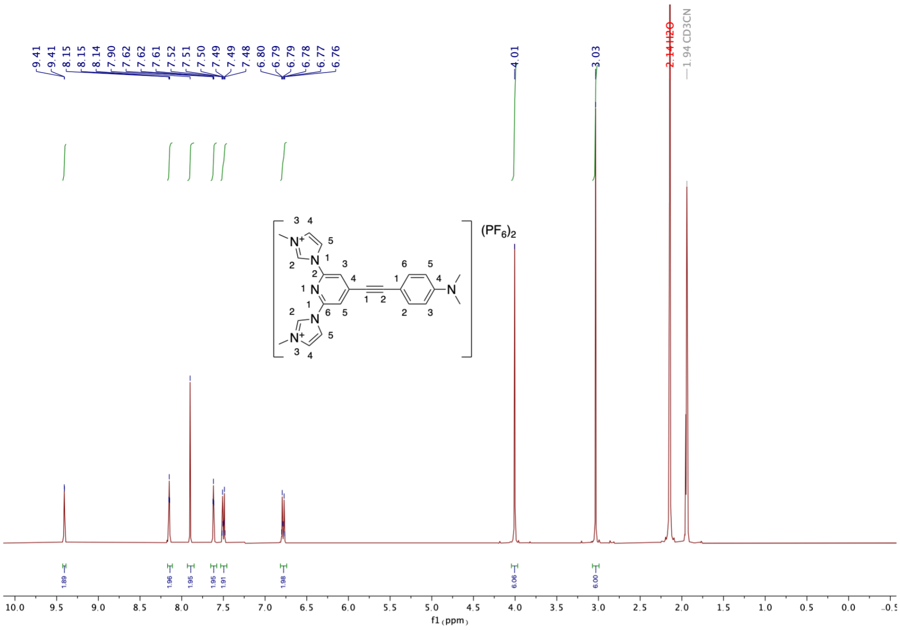

Figure S25: ^{1}H NMR (CD_3CN) spectrum of 1,1'-(4-((4-(dimethylamino)phenyl)ethynyl)pyridine-2,6-diyl)bis(3-methylimidazolium)bis(hexafluorophosphate) (**12**).

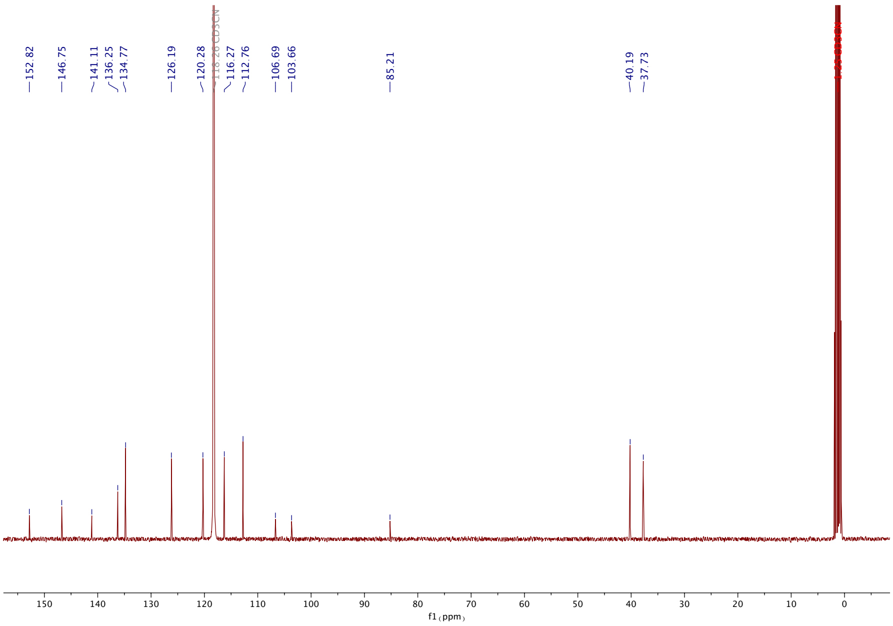

Figure S26: ^{13}C NMR (CD_3CN) spectrum of 1,1'-(4-((4-(dimethylamino)phenyl)ethynyl)pyridine-2,6-diyl)bis(3-methylimidazolium)bis(hexafluorophosphate) (**12**).

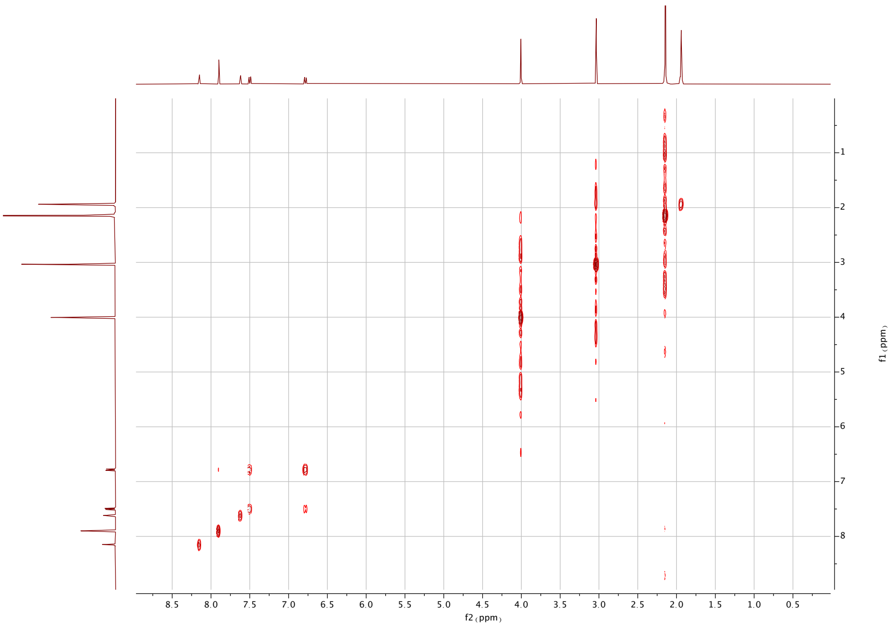

Figure S27: COSY NMR (CD_3CN) spectrum of 1,1'-(4-((4-(dimethylamino)phenyl)ethynyl)pyridine-2,6-diyl)bis(3-methylimidazolium)bis(hexafluorophosphate) (**12**).

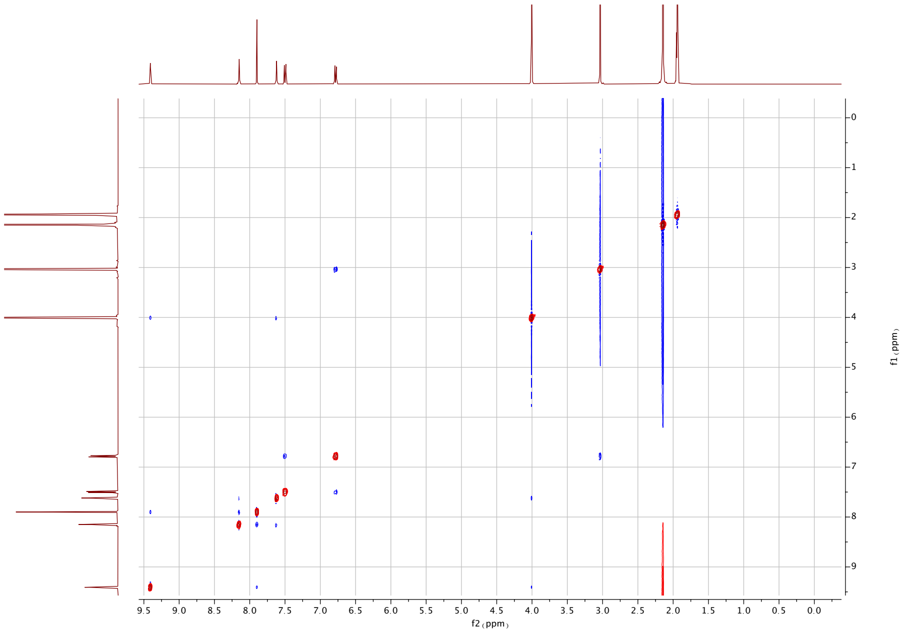

Figure S28: NOESY NMR (CD_3CN) spectrum of 1,1'-(4-((4-(dimethylamino)phenyl)ethynyl)pyridine-2,6-diyl)bis(3-methylimidazolium)bis(hexafluorophosphate) (**12**).

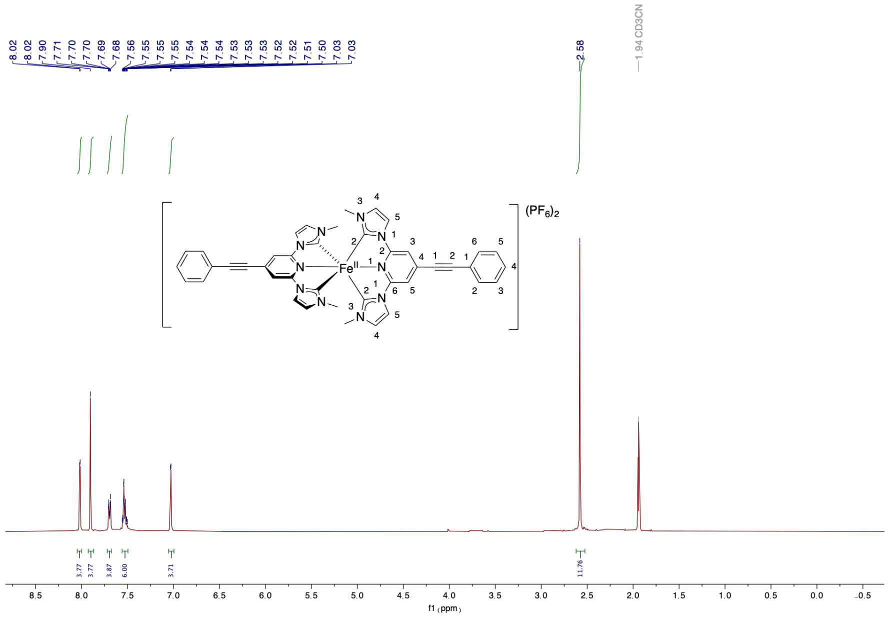

Figure S29: ^{1}H NMR (CD_3CN) spectrum of bis(1,1´-(4-((phenyl)ethynyl)pyridine-2,6-diyl)bis(methylimidazolylidene))iron bis(hexafluorophosphate) (**2**).

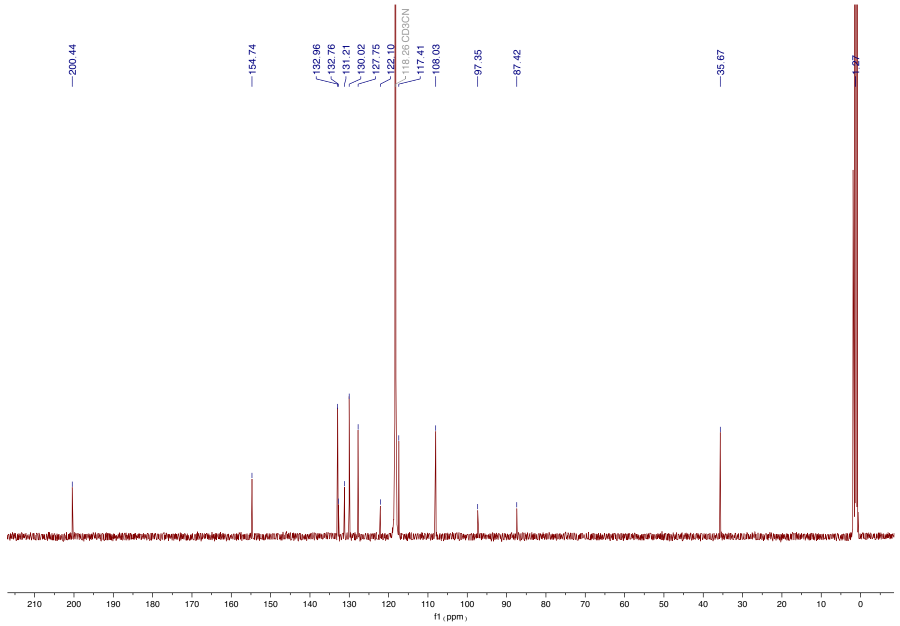

Figure S30: ^{13}C NMR (CD_3CN) spectrum of bis(1,1´-(4-((phenyl)ethynyl)pyridine-2,6-diyl)bis(methylimidazolylidene))iron bis(hexafluorophosphate) (**2**).

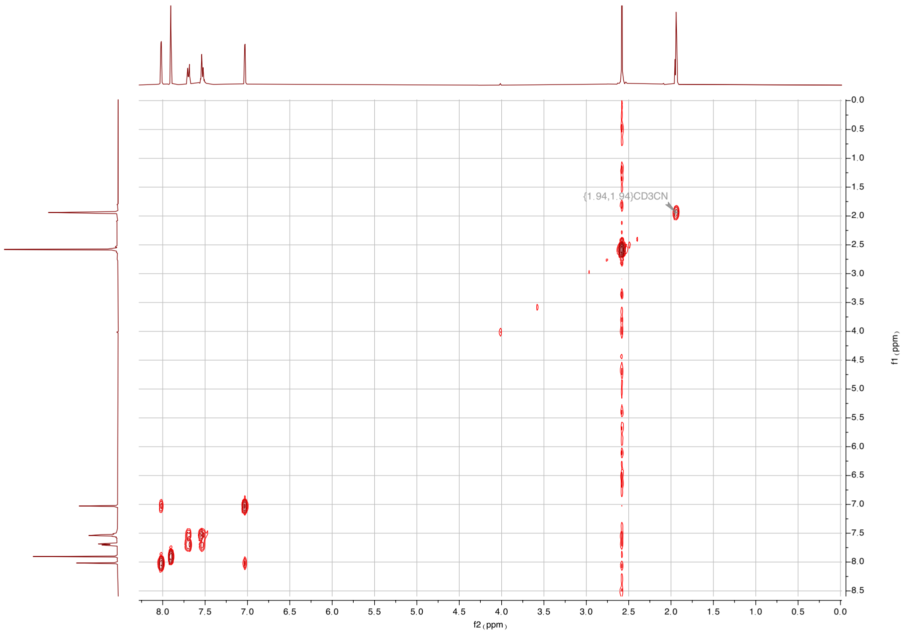

Figure S31: COSY NMR (CD_3CN) spectrum of bis(1,1´-(4-((phenyl)ethynyl)pyridine-2,6-diyl)bis(methylimidazolylidene))iron bis(hexafluorophosphate) (**2**).

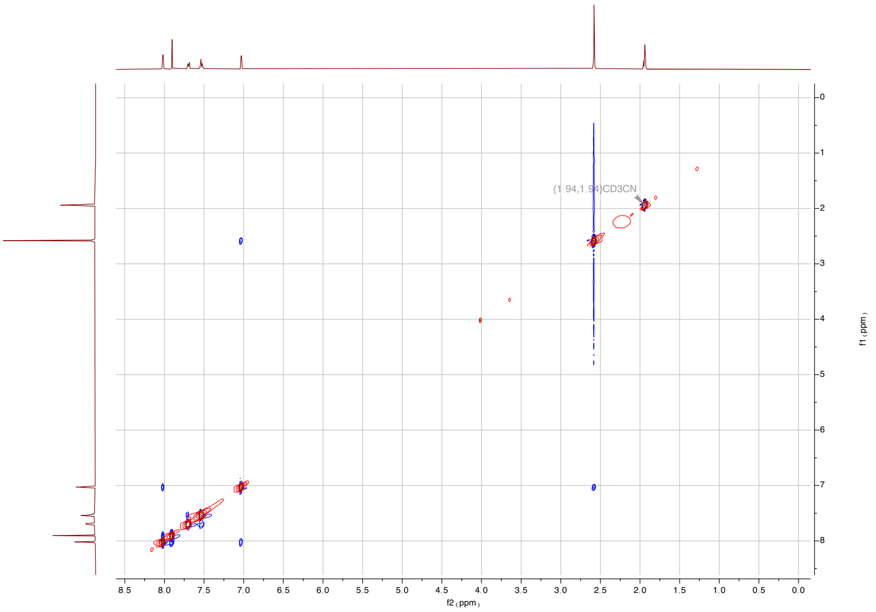

Figure S32: NOESY NMR (CD_3CN) spectrum of bis(1,1´-(4-((phenyl)ethynyl)pyridine-2,6-diyl)bis(methylimidazolylidene))iron bis(hexafluorophosphate) (**2**).

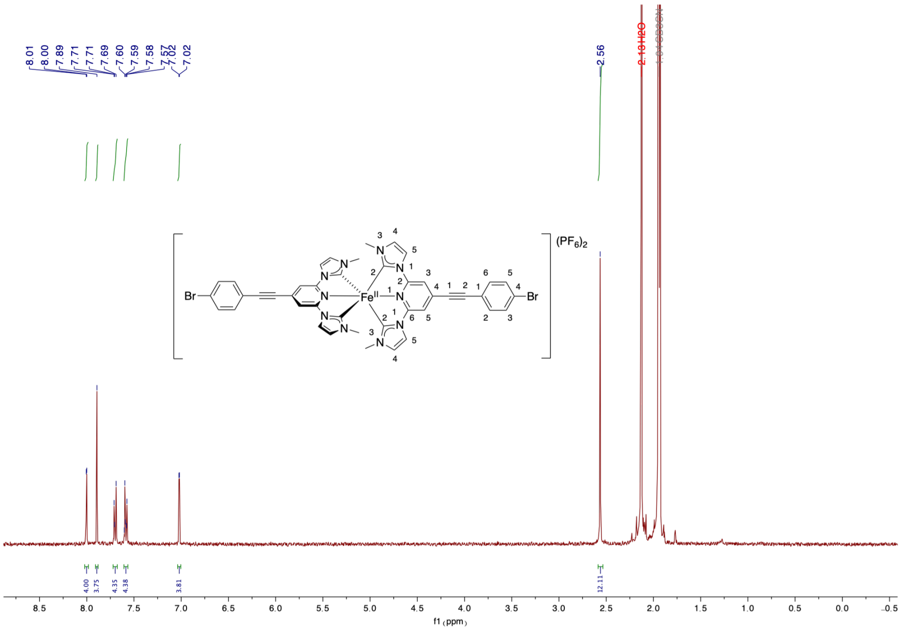

Figure S33: ^{1}H NMR (CD_3CN) spectrum of bis(1,1´-(4-((4-bromophenyl)ethynyl)pyridine-2,6-diyl)bis(methylimidazolylidene))iron bis(hexafluorophosphate) (**3**).

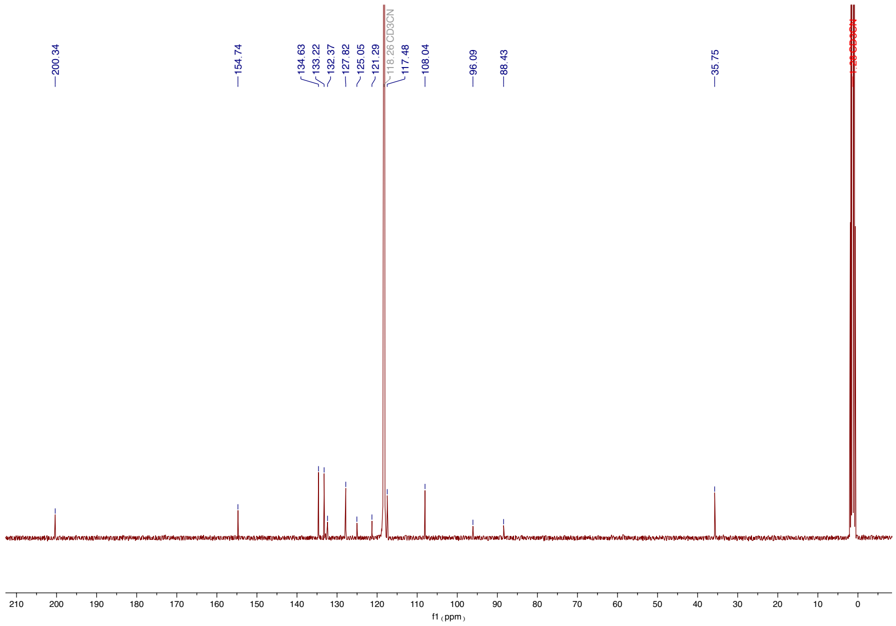

Figure S34: ^{13}C NMR (CD_3CN) spectrum of bis(1,1´-(4-((4-bromophenyl)ethynyl)pyridine-2,6-diyl)bis(methylimidazolylidene))iron bis(hexafluorophosphate) (**3**).

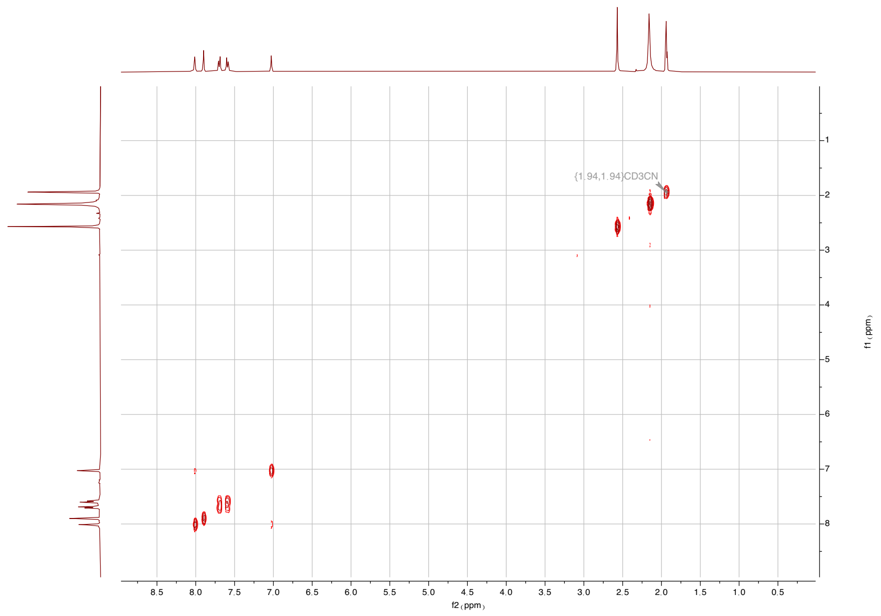

Figure S35: COSY NMR (CD_3CN) spectrum of bis(1,1´-(4-((4-bromophenyl)ethynyl)pyridine-2,6-diyl)bis(methylimidazolylidene))iron bis(hexafluorophosphate) (**3**).

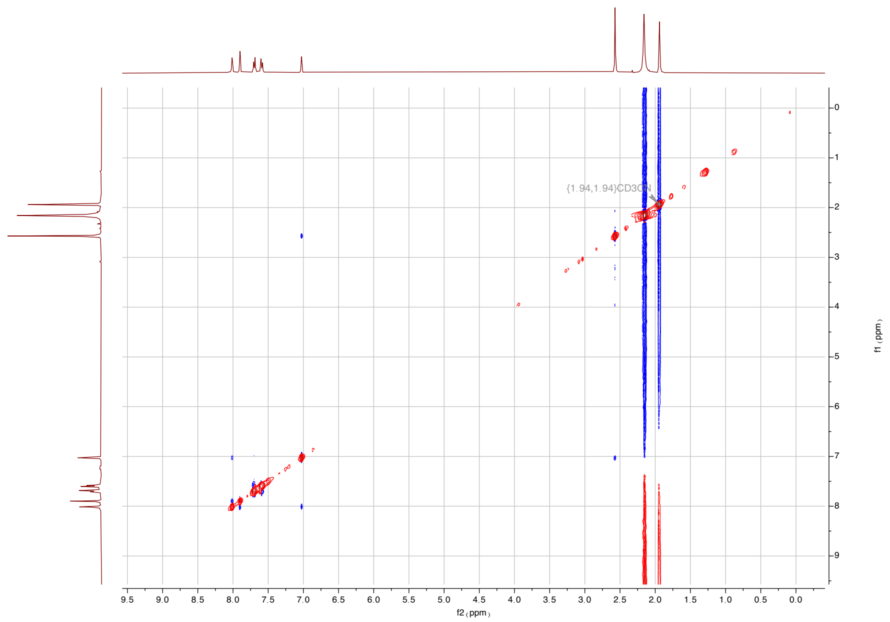

Figure S36: NOESY NMR (CD_3CN) spectrum of bis(1,1´-(4-((4-bromophenyl)ethynyl)pyridine-2,6-diyl)bis(methylimidazolylidene))iron bis(hexafluorophosphate) (**3**).

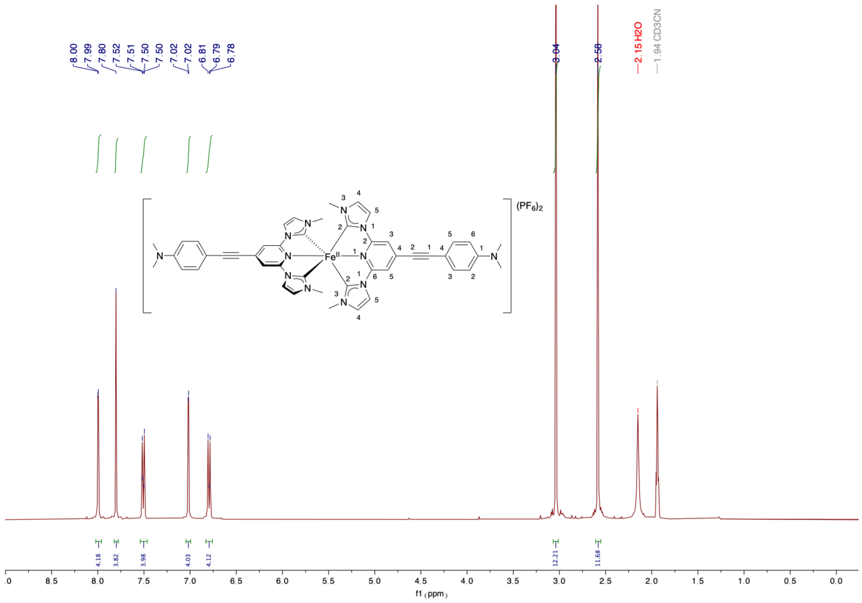

Figure S37: ^{1}H NMR (CD_3CN) spectrum of bis(1,1´-(4-((4-(*N,N*-dimethylammino)phenyl)ethynyl)pyridine-2,6-diyl)bis(methylimidazolylidene))iron bis(hexafluorophosphate) (**4**).

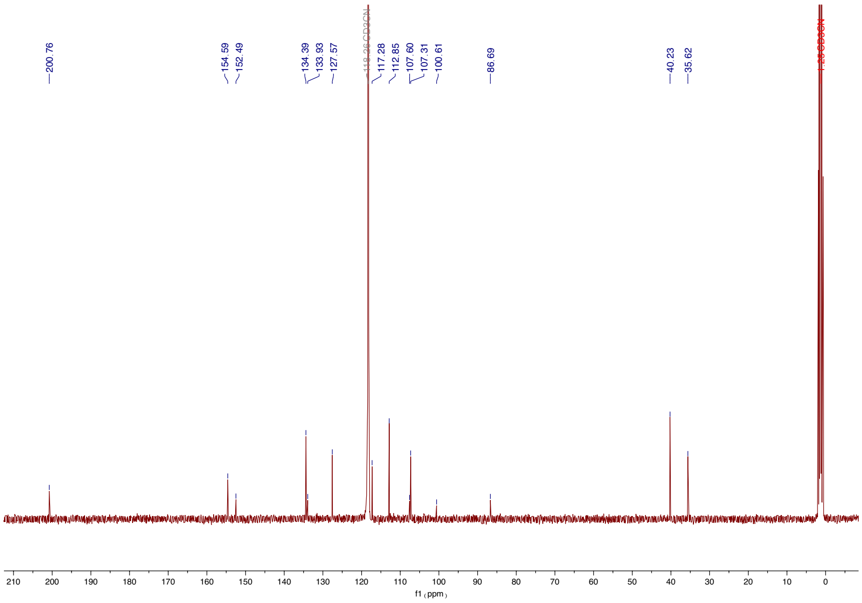

Figure S38: ^{13}C NMR (CD_3CN) spectrum of bis(1,1´-(4-((4-(*N,N*-dimethylammino)phenyl)ethynyl)pyridine-2,6-diyl)bis(methylimidazolylidene))iron bis(hexafluorophosphate) (**4**).

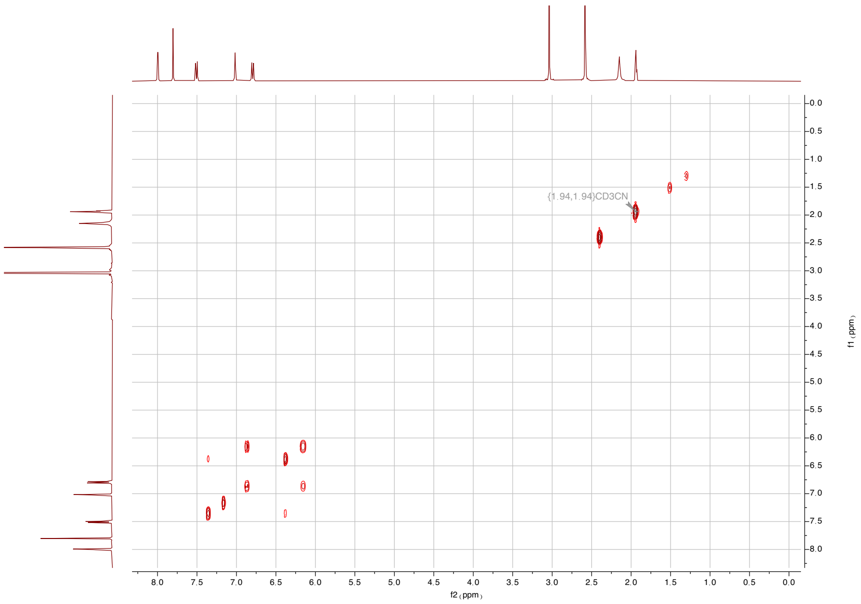

Figure S39: COSY NMR (CD_3CN) spectrum of bis(1,1´-(4-((4-(*N,N*-dimethylammino)phenyl)ethynyl)pyridine-2,6-diyl)bis(methylimidazolylidene))iron bis(hexafluorophosphate) (**4**).

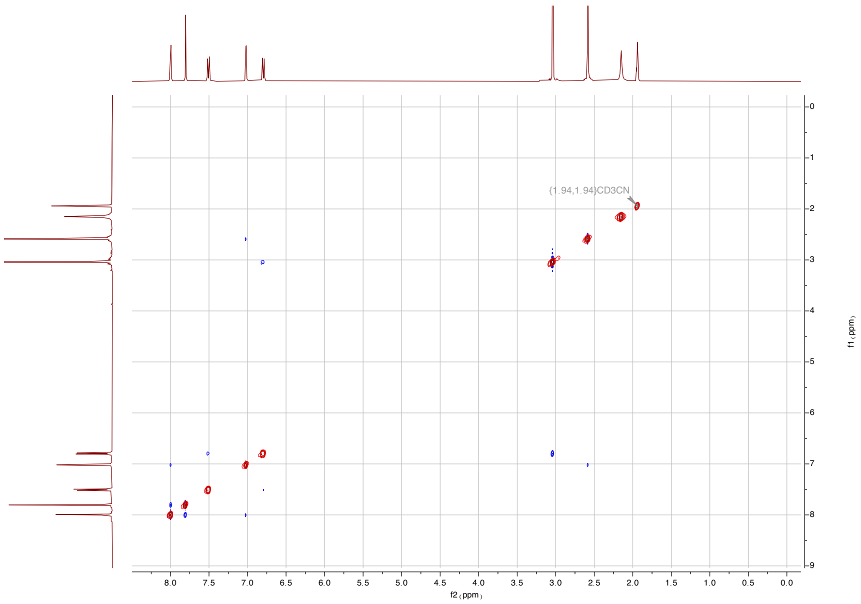

Figure S40: NOESY NMR (CD_3CN) spectrum of bis(1,1´-(4-((4-(*N,N*-dimethylammino)phenyl)ethynyl)pyridine-2,6-diyl)bis(methylimidazolylidene))iron bis(hexafluorophosphate) (**4**).

Single Crystal X-ray Structure Determination

The diffraction data for $[Fe(II)(depbmi)_2](PF_6)_2$ (**4**) suggests the space group I-42d or $I4_1md$. The structure was somewhat difficult to solve, due to symmetry breaking features in structure. The solution in I-42d is meaningful, and showed clearly that the axial mirror plane in $I4_1$**m**d is not present. If this space group were used as a starting point, it would therefore have to be reduced at least to $I4_1$.

We therefore started in I-42d as this provided a starting model that could readily be interpreted. There was however an issue with this space group choice. First, the Fe position shows a large anisotropic thermal parameter U33, indicating an extension of this position over 1Å along the c-axis, a clearly unphysical situation. If anisotropy was applied to all atoms, they show similar behavior with large,

anisotropic thermal parameters. This could alternatively be modelled using split positions, indicating a lower local symmetry, violating the I-42d space group.

This can be explained in two different ways. First, the structure did not crystallize in I-42d, but in a subgroup where the operation that relate the two (split) Fe positions is absent. There are two operations that relate the two Fe positions, the -4 rotoinversion and the 2-fold axis along a (and b). The 2-fold axis that was the result of applying the -4 operation twice is unproblematic, as are the d-glides. Thus, Fdd2 would be a possible subgroup. However, Fdd2 unfortunately related the Fe positions in the unit cell in such a way that although the local symmetry relating the split pair directly is absent, there is still a global relationship between the Fe positions such that all Fe positions must be in z, z+1/4, z+1/2 and z+3/4. the structure is allowed to slide along the c axis, but the relative positions between Fe atoms remains the same. The symmetry must be reduced all the way down to I2 for this relationship to be released. Performing this reduction is possible using a large number of constraints, but the refinement remains unsatisfactory. The high symmetry model with splits or large thermal parameters remains superior to a reduced symmetry model.

The second way to explain the above issues was that the split model indicated that the structure is disordered at a relatively small length scale. This is different to a low symmetry model only in practice, but not really in principle. A split model indicates that the low symmetry structure (I2) does not form as large blocks of many unit cells, but that the twinning is much more frequent, and thus the diffraction pattern is better described with disorder than with macroscopic twinning. This means a rather high degree of disorder and results in rather poor R-values.

We therefore refined the structure in I-42d, using anisotropic thermal displacement parameters. The reason for doing this was that it allowed more reliable modelling of interatomic distances. In a split model, the whole ligand molecule must move as a rigid body together with Fe and this meant constraining the Fe-N distance. The model allowed for a straightforward measurement of the torsion angles between the two six-ring systems and between the C6 ring and the terminal dimethylamine group.

Table S1: Crystal data and structure refinement for Complexes **2**, **3** and **4**.

Identification code	[Fe(pepbmi)$_2$](PF$_6$)$_2$ (**2**)	[Fe(bepbmi)$_2$](PF$_6$)$_2$ (**3**)	[Fe(depbmi)$_2$](PF$_6$)$_2$ (**4**)
Empirical formula	$C_{48.51}H_{43.12}F_{12}FeN_{12.38}P_2$	$C_{52}H_{44.5}Br_2F_{12}FeN_{11.5}P_2$	$C_{46}H_{44}F_{13.019}FeN_{12}P_{1.941}$
Formula weight	1145.30	1336.10	1128.2
Temperature/K	100	100.00	293
Crystal system	monoclinic	orthorhombic	tetragonal
Space group	C2	Pbcn	I-42d
a/Å	30.4919(5)	10.3416(5)	11.4354(15)
b/Å	11.70450(10)	40.272(3)	11.4354(15)
c/Å	32.4889(6)	14.0711(10)	44.724(7)
α/°	90	90	90
β/°	116.096(2)	90	90
γ/°	90	90	90
Volume/Å^3	10413.0(3)	5860.3(6)	5848.5(14)
Z	8	4	4
ρ_{calc} g/cm^3	1.461	1.514	1.2814
μ/mm^{-1}	0.442	1.760	0.393
F(000)	4679.0	2684.0	2301.0
Crystal size/mm^3	0.11 × 0.07 × 0.05	0.15 × 0.09 × 0.03	0.2 x 0.08 x 0.04
Radiation	Mo Kα (λ = 0.71073)	Mo Kα (λ = 0.71073)	Mo Kα (λ = 0.71073)
2Θ range for data collection/°	6.9 to 49.998	6.726 to 49.996	3.56 to 30.87
Index ranges	-36 ≤ h ≤ 36, -13 ≤ k ≤ 13, -38 ≤ l ≤ 38	-12 ≤ h ≤ 12, -47 ≤ k ≤ 47, -16 ≤ l ≤ 16	-9 ≤ h ≤ 15, -15 ≤ k ≤ 9, -18 ≤ l ≤ 59
Reflections collected	62298	35414	7397
Independent reflections	18141 [R_{int} = 0.0338, R_{sigma} = 0.0401]	5162 [R_{int} = 0.1266, R_{sigma} = 0.1118]	3858 [R_{int} = 3.14/6.80 (obs/all), R_{sigma} = 4.24/13.86 obs/all]
Data/restraints/parameters	18141/85/1203	5162/84/293	194/33/89
Goodness-of-fit on F^2	1.024	1.024	1.90

Final R indexes [I>=2σ (I)]	R_1 = 0.0432, wR_2 = 0.0997	R_1 = 0.1022, wR_2 = 0.2654	R_1 = 0.1277, wR_2 = 0.1299
Final R indexes [all data]	R_1 = 0.0527, wR_2 = 0.1044	R_1 = 0.1847, wR_2 = 0.3208	R_1 = 0.4109, wR_2 = 0.1532
Largest diff. peak/hole / e $Å^{-3}$	0.68/-0.33	1.32/-0.41	0.55/-0.45
Flack parameter	-0.015(4)	-	-
CCDC number	2403470	2403472	2434263

Table S2: Angles between the bonds on either side of ethynyl bridge in complex **2**.

**Bonds**	**Angle (°)**
(C5-C4) – (C9-C13)	12.643
(C5-C4) – (C9-C10)	16.951
(C3-C4) – (C9-C10)	17.854
(C3-C4) – (C9-C13)	13.546
Average	15.249

Table S3: Angles between the bonds on either side of ethynyl bridge in complex **3**.

**Bonds**	**Angle (°)**
(C5-C4) – (C9-C13)	18.187
(C5-C4) – (C9-C10)	18.316
(C3-C4) – (C9-C10)	16.279
(C3-C4) – (C9-C13)	16.150
Average	17.233

Table S4: Angles between units in complex **4**.

**Units compared**	**Angle (°)**
Phenylene-pyridine	3.7
Me_2N-Phenylene	2.48

Steady-State Absorption Spectra

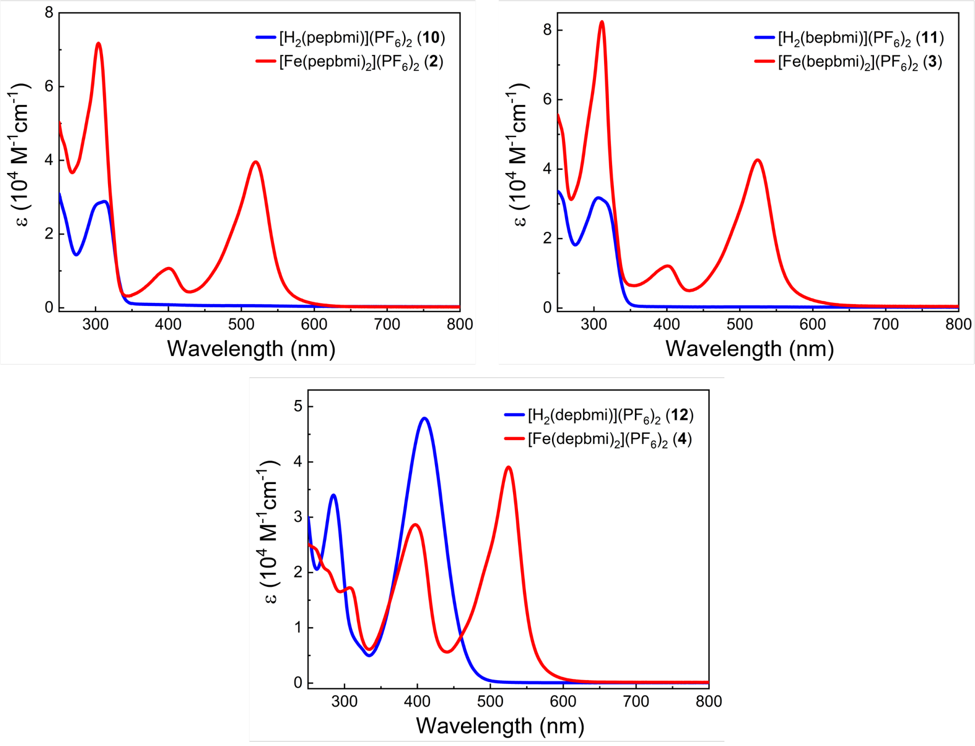

Figure S41: UV-Vis Absorption spectra of ligands **10**, **11** and **12**, and complexes **2**, **3** and **4** in acetonitrile.

Quantum chemical calculations

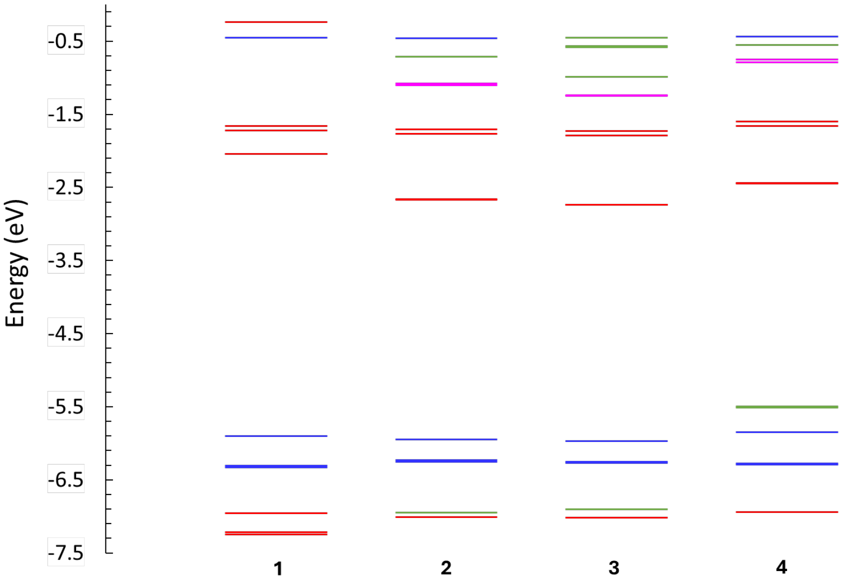

Figure S42: Molecular orbital energy-level diagrams of complexes **1-4**. MOs centered predominantly on Fe are shown in blue, C^N^C-ligand centered MOs are in red, phenyl-ethynyl–centered MOs are in green, and MOs delocalized over the entire ligand are shown in pink.

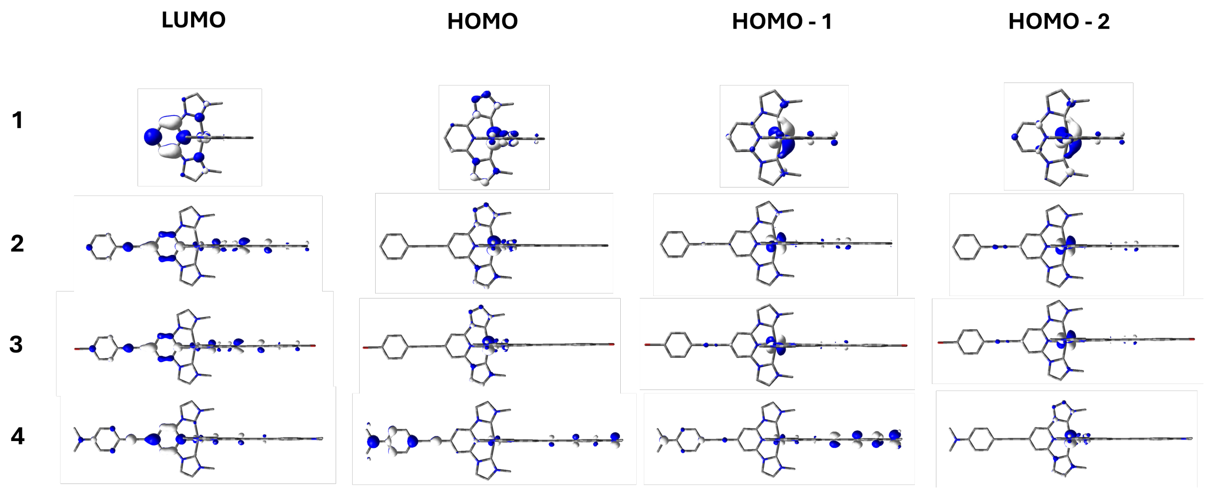

Figure S43: Frontier MOs of complexes **1**-**4**.

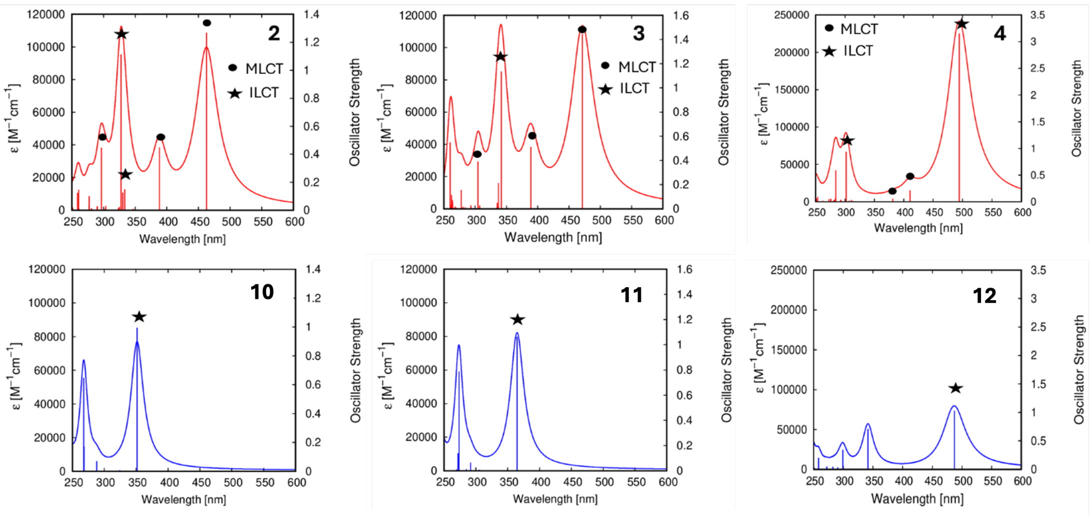

Figure S44: Calculated UV-Vis spectra for complexes **2**-**4** are shown in red, while the UV-Vis spectra for the uncoordinated ligands of complexes **2**-**4** (**10**-**12** respectively) are shown in blue. (For consistency, all ligand-based trannsitions are reffered to as ILCT, although the transitions in complexes **2** and **3** have a mixed ligand-centered/intra-ligand charge transfer character).

Additional Analysis of the Calculated UV-Vis Spectra

The calculated UV–Vis spectra for complexes **2**-**4** along with spectra of their uncoordinated ligands are shown in Figure S44. While complexes **2** and **3** provide a relatively good match for the experimental spectrum, the calculated spectrum of **4** does not reproduce the experimental spectrum. Looking more carefully at the spectra of **2** and **3**, we can observe a blue shift in the calculated MLCT bands (at 460 nm for **2** and 470 nm for **3**) relative to the experimental spectrum (520 nm for **2** and **3**). On the other hand, the calculated spectra for the uncoordinated ligands are red shifted with ligand center (LC) transitions at 350 nm for **2** (vs. 300 nm experimental) and 360 nm for **3** (vs. 300 nm experimental), these transitions have a slightly charge transfer character as shown in Figure S45. Despite the blue shift of the MLCT bands and the slight red shift of the LC bands, the MLCT bands of complexes **2** and **3** are still calculated at lower energies than the LC bands, leading to overall acceptable shape of the calculated spectra.

For complex **4**, the lowest energy ligand-based transition holds a higher percentage of charge transfer as shown in Figure S45, where it could be characterized as Inter-ligand charge transfer (ILCT), which results in a more prominent shift than for complexes **2** and **3**. This is likely due to the presence of the strongly donating dimethyl amine substituent. As shown in Figure S44, the lowest energy transition corresponding to HOMO-LUMO excitation for complex **4** is ILCT, appearing at around 500 nm. This

is consistent with similar peak appearing in same region of ligand spectrum as shown in Figure S44. However, MLCT states are calculated at a higher energy (400 nm) due to lower t_{2g} set in complex **4** than that in complexes **2** & **3.**

We have calculated the spectra of complexes **2-4** at different levels of theory to see if we can mitigate the problem with the red shift of the ILCT transitions vs. the blue shift of the MLCT transitions. Unfortunately, the use of various functionals, including the long-range corrected functionals such as CAM-B3LYP, results in the calculated spectra of similar shape as those obtained with the B3LYP functional (see Figure S46). Additionally, we examined the impact of conformational flexibility on all three complexes by rotating the phenylacetylene substituent to a perpendicular position relative to the CAB ligand moiety (Figure S47). The rotational barrier is around 1.5 kcal/mol for **2** and **3** and 2.7 kcal/mol for **4**, indicating that such ligand rotations are feasible. Averaging the UV–Vis spectra of different conformers revealed minor effects on peak intensities rather than their positions as shown in Figure S48 suggesting that the observed spectral shifts are more likely due to DFT failure rather than conformational flexibility.

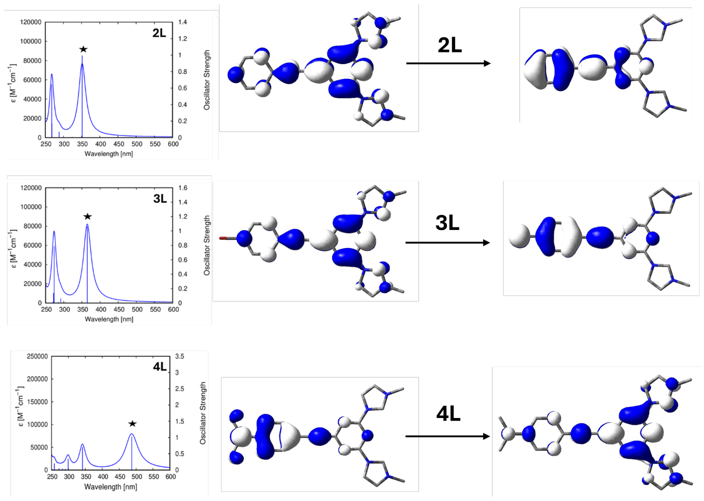

Figure S45: Calculated UV-Vis spectra for the uncoordinated ligands of complexes **2-4** (**10-12** respectively) with the assigned character of the lowest energy transition.

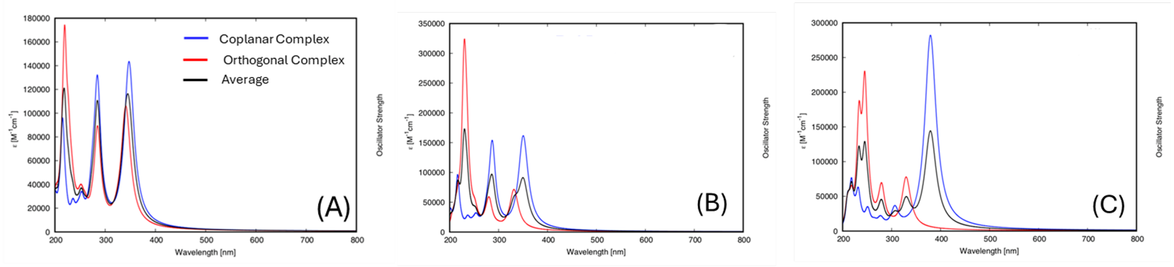

Figure S46: Calculated UV-Vis spectra for complexes 2(A), 3(B), 4(C) at CAM-B3LYP.

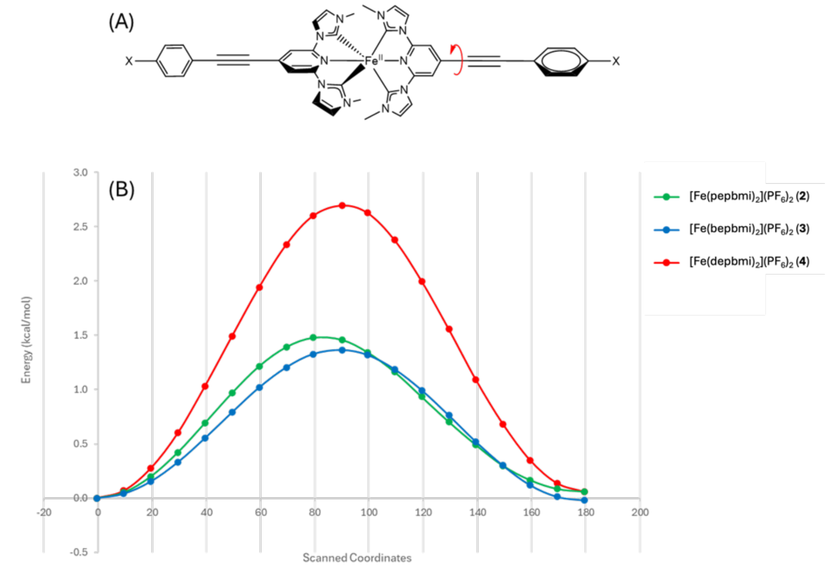

Figure S47: (A) Scheme showing the rotated dihedral angle in ligand. (B) Potential energy surface for the scanned dihedral angle.

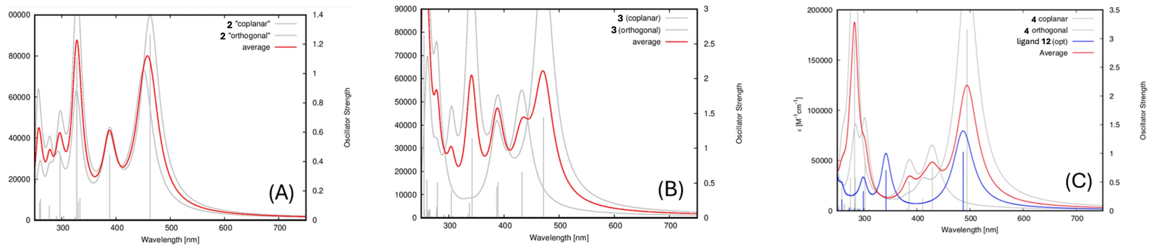

Figure S48: Averaged UV-vis spectra of orthogonal and coplanar conformers of complexes **2** (A), **3** (B), and **4** (C). (C) also include spectrum for the ligand of **4**, **12**, in its fully optimized form.

Trends in the Excited-State Energetics

To better understand how the excited states with different characteristics evolve across the series of Fe(II) complexes (**2, 3** and **4**), the energies of the key excited states relative to the ground state energy for each complex are plotted in Figure S47. The singlet ground state energy is set to zero, and the energies of the fully optimized metal-centred triplet and quintet states (denoted as ^{3}MC and ^{5}MC, respectively) are included. Additionally, the energies of the lowest-energy singlet and triplet excited states with MLCT are presented, as determined from time-dependent density functional theory with additional Tamm-Dancoff Approximation (TDA) calculations using the ^{1}A reference state.

From the vertical excitation potential energy curve (PEC) of complex **2,** Figure S47A, it is observed that the calculated vertical excitation reveals a ^{3}MLCT state to be the lowest excited state with ^{1}MLCT and ^{3}MC of 0.14, and 0.03 eV higher in energy, respectively at the ground state optimized geometry. Deactivation from a higher energy ^{1}MLCT leads back to ^{1}A through a radiative decay since an ISC to ^{3}MC is spin forbidden.

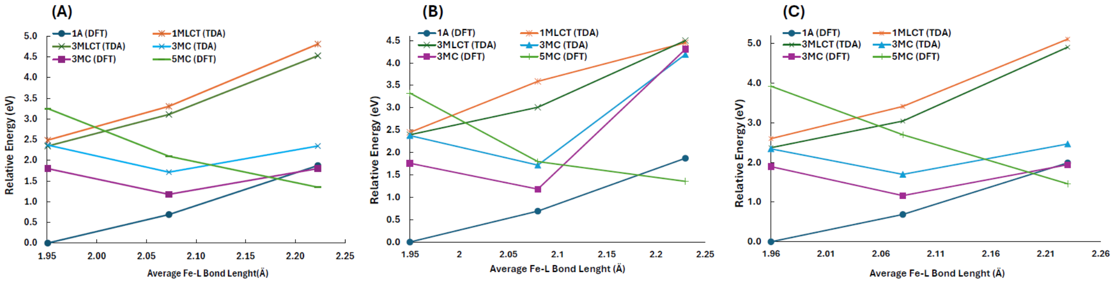

Figure S49: Potential energy curves for the relevant electronic states of Complex **2** (A), while that of Complex **3** (B), & Complex **4** (C), from energies obtained at the ^{1}A (R = 1.95 Å), ^{3}MC (R = 2.08 Å), and ^{5}MC (R = 2.23 Å) optimized structures from single point energy calculations at the DFT (^{1}A, ^{3}MC, ^{5}MC) and TDA levels of theory (^{1}MLCT, ^{3}MLCT) utilizing ^{1}A as the reference state. The reaction coordinate is given as the average of Fe-L bond lengths at each optimized structure.

For complex **3**, the PEC displayed in Figure S47B shows a different character than that of **2**, where the lowest energy excited state is a ^{3}MC which is slightly lower in energy than the ^{3}MLCT and the ^{1}MLCT at 0.02 and 0.07 eV respectively at the ground state optimized geometry. Similar case is observed for complex **4**, where lowest energy excitation computed in ^{3}MC resembling that of complex **3**, which is lower in energy than ^{3}MLCT and ^{1}MLCT at 0.03 and 0.26 eV respectively.

For both complexes, deactivation from ^{1}MLCT leads back to ^{1}A through radiative decay, due to spin forbidden ISC to the lower ^{3}MC excited states. However, upon excitation to the higher ^{3}MLCT state, deactivation through the lower ^{3}MC state is most likely to take place through a non-radiative decay process. Note that ^{5}MC for complex **4** is not a fully optimized metal-centered state, yet it is essentially a converged minimum.

Transient absorption spectroscopy

400 nm excitation

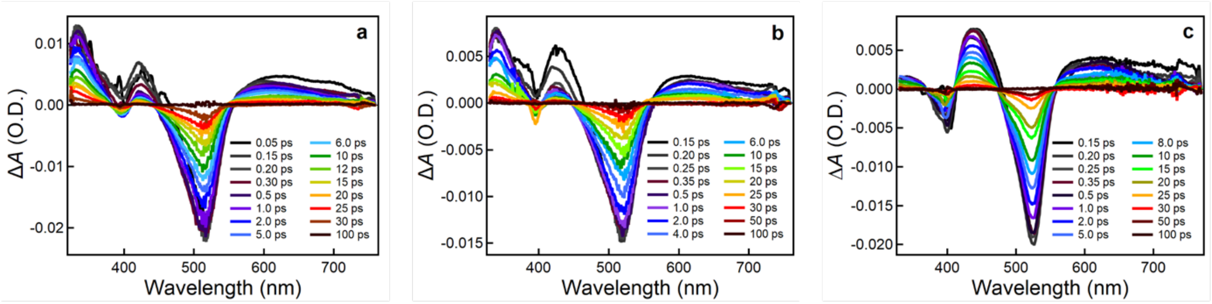

Figure S50: Transient absorption spectra at selected delay times of (**a**) $[Fe(pepbmi)_2](PF_6)_2$ (**2**), (**b**) $[Fe(bepbmi)_2](PF_6)_2$ (**3**) and (**c**) $[Fe(depbmi)_2](PF_6)_2$ (**4**). All complexes in acetonitrile, excitation wavelength 400 nm.

MLCT reference spectra

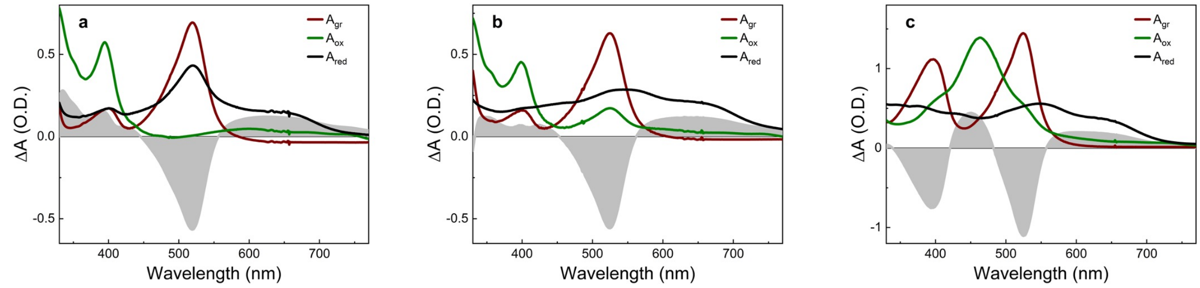

Figure S51: MLCT reference spectra obtained from ground state absorption spectra (A_{gr}) and spectra of the oxidized (A_{ox}) and reduced (A_{red}) complexes according to ref.S17 with η=0.4 for all complexes. (**a**) $[Fe(pepbmi)_2](PF_6)_2$ (**2**), (**b**) $[Fe(bepbmi)_2](PF_6)_2$ (**3**) and (**c**) $[Fe(depbmi)_2](PF_6)_2$ (**4**).

Data fitting

For global analysis of the TA data[S18-S20] a Python script provided by Johannes Wega (University of Geneva) was used.

Fit results: Sequential model

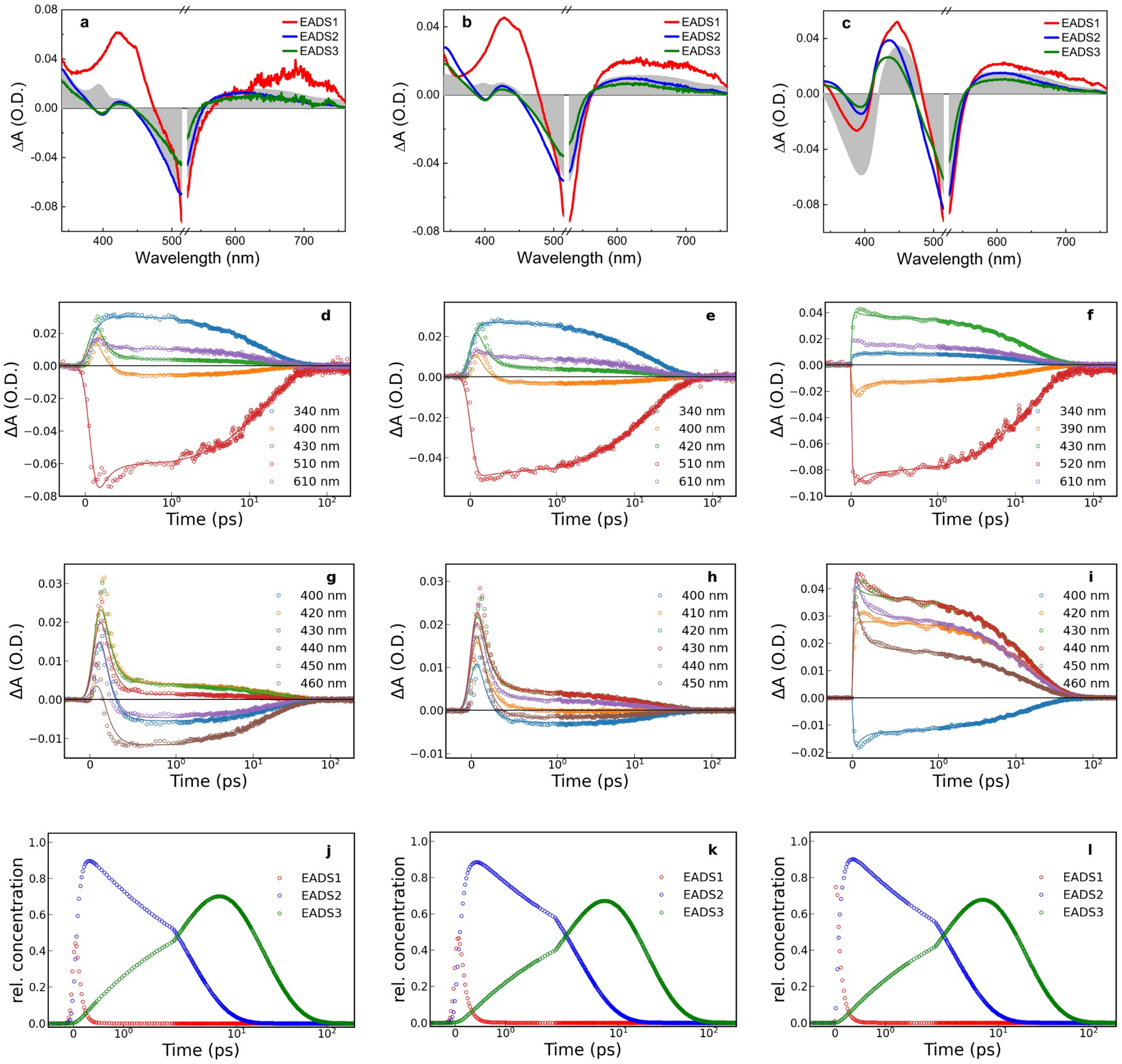

Figure S52: Global analysis results for the transient absorption spectra shown in Figure 7 (excited at 525 nm) based on sequential decay model. Left column: $[Fe(pepbmi)_2](PF_6)_2$ (**2**), middle column: $[Fe(bepbmi)_2](PF_6)_2$ (**3**), right column: $[Fe(depbmi)_2](PF_6)_2$ (**4**). **a-c:** Evolution-associated difference spectra (EADS) and MLCT reference spectra (shaded area) from spectroelectrochemistry. **d-i:** Transient absorption kinetics fit results (solid lines) at selected wavelengths. **j-l:** Concentration kinetics.

Table S5: Time constants returned by global analysis for the fit results shown in Figure S52.

**Complex**	**τ_1 (ps)**	**τ_2 (ps)**	**τ_3 (ps)**
**$[Fe(pepbmi)_2](PF_6)_2$ (2)**	< 0.2 ps	2.9 ps	17.9 ps
**$[Fe(bepbmi)_2](PF_6)_2$ (3)**	< 0.2 ps	3.4 ps	17.3 ps
**$[Fe(depbmi)_2](PF_6)_2$ (4)**	< 0.2 ps	3.2 ps	17.0 ps

Fit results: Parallel model

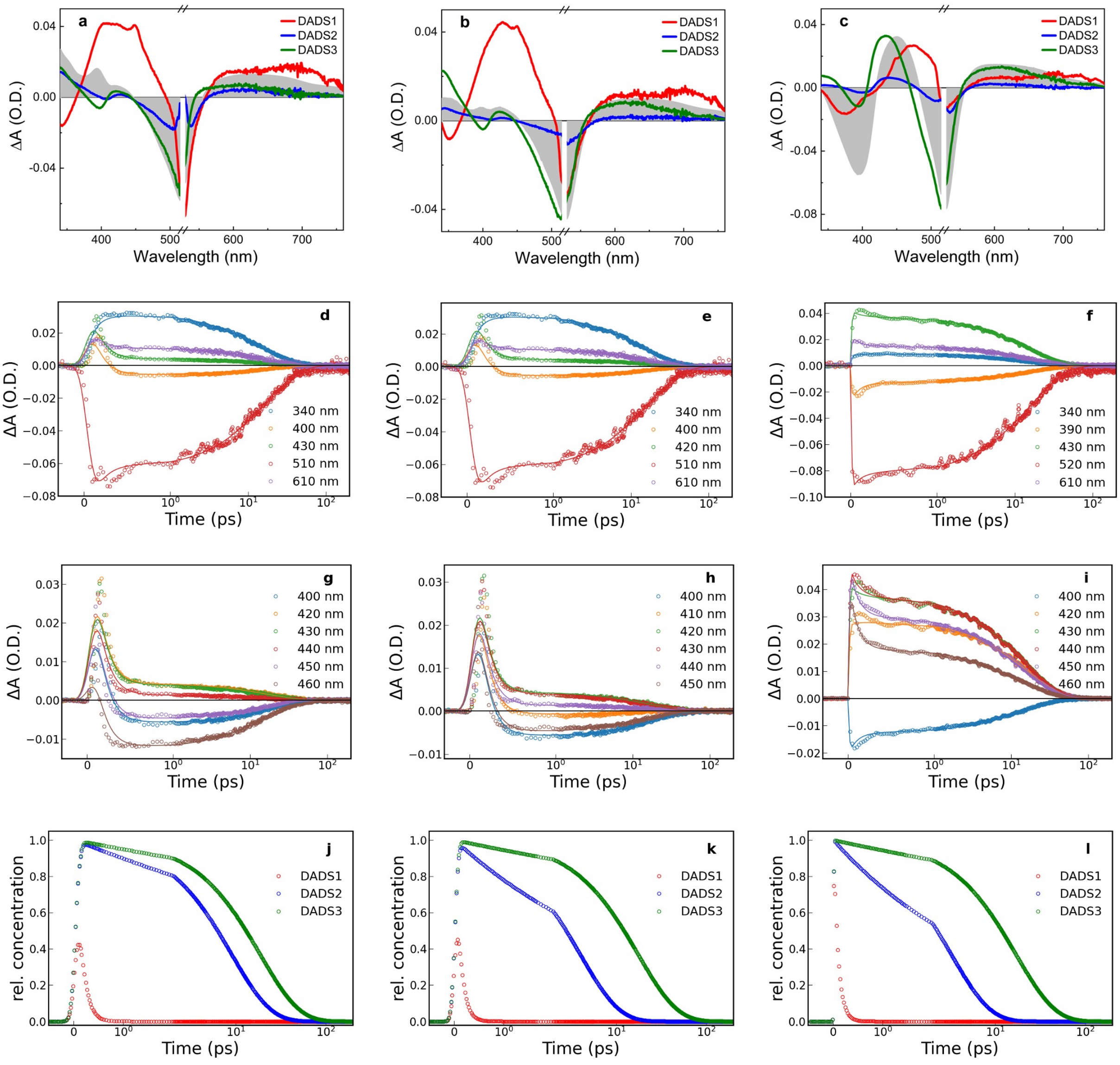

Figure S53: Global analysis results for the transient absorption spectra shown in Figure 7 (excited at 525 nm) based on a parallel decay model. Left column: $[Fe(pepbmi)_2](PF_6)_2$ (**2**), middle column: $[Fe(bepbmi)_2](PF_6)_2$ (**3**), right column: $[Fe(depbmi)_2](PF_6)_2$ (**4**). **a-c:** Decay-associated difference spectra (DADS). **d-i:** Transient absorption kinetics fit results (solid lines) at selected wavelengths. **j-l:** Concentration kinetics.

Table S6: Time constants returned by global analysis for the fit results shown in Figure S53.

**Complex**	**τ_1 (ps)**	**τ_2 (ps)**	**τ_3 (ps)**
**$[Fe(pepbmi)_2](PF_6)_2$ (2)**	< 0.2 ps	8.9 ps	18.6 ps
**$[Fe(bepbmi)_2](PF_6)_2$ (3)**	< 0.2 ps	3.9 ps	17.5 ps
**$[Fe(depbmi)_2](PF_6)_2$ (4)**	< 0.2 ps	3.2 ps	17.0 ps

References

S1. Haynes, W. M., *CRC Handbook of Chemistry and Physics, 97th Edition*. CRC Press: 2016.

S2. Weast, R. C., *CRC handbook of chemistry and physics Sixty-seventh edition*. CRC Press Inc: United States, 1986.

S3. Becke, A. D., Densityfunctional thermochemistry. III. the role of exact exchange. *J. Chem. Phys.* **1993,** *98* (7), 5648–5652.

S4. Grimme, S., Semiempirical GGA-type density functional constructed with a long-range dispersion correction. *J. Comput. Chem.* **2006,** *27* (15), 1787-1799.

S5. Becke, A. D., A new mixing of Hartree–Fock and local density-functional theories. *J. Chem. Phys.* **1993,** *98* (2), 1372-1377.

S6. Lee, C.; Yang, W.; Parr, R. G., Development of the Colle-Salvetti correlation-energy formula into a functional of the electron density. *Physical review B* **1988,** *37* (2), 785.

S7. Becke, A. D., Density-functional exchange-energy approximation with correct asymptotic behavior. *Physical review A* **1988,** *38* (6), 3098.

S8. Kaupp, M.; Schleyer, P. v. R.; Stoll, H.; Preuss, H., Pseudopotential approaches to Ca, Sr, and Ba hydrides. Why are some alkaline earth MX2 compounds bent? *J. Chem. Phys.* **1991,** *94* (2), 1360-1366.

S9. Hehre, W. J.; Ditchfield, R.; Pople, J. A., Self—consistent molecular orbital methods. XII. Further extensions of Gaussian—type basis sets for use in molecular orbital studies of organic molecules. *J. Chem. Phys.* **1972,** *56* (5), 2257-2261.

S10. Scalmani, G.; Frisch, M. J., Continuous surface charge polarizable continuum models of solvation. I. General formalism. *J. Chem. Phys.* **2010,** *132* (11).

S11. Frisch, M. e.; Trucks, G.; Schlegel, H. B.; Scuseria, G.; Robb, M.; Cheeseman, J.; Scalmani, G.; Barone, V.; Petersson, G.; Nakatsuji, H., Gaussian 16, Revision A.03. Gaussian, Inc., Wallingford CT: 2016.

S12. Gorelsky, S. I., AOMix: Program for molecular orbital analysis, version 6.94. 2013.

S13. Hirata, S.; Head-Gordon, M., Time-dependent density functional theory within the Tamm–Dancoff approximation. *Chem. Phys. Lett.* **1999,** *314* (3-4), 291-299.

S14. Staroverov, V. N.; Scuseria, G. E.; Tao, J.; Perdew, J. P., Comparative assessment of a new nonempirical density functional: Molecules and hydrogen-bonded complexes. *J. Chem. Phys.* **2003,** *119* (23), 12129-12137.

S15. Tao, J.; Perdew, J. P.; Staroverov, V. N.; Scuseria, G. E., Climbing the density functional ladder: Nonempirical meta–generalized gradient approximation designed for molecules and solids. *Phys. Rev. Lett.* **2003,** *91* (14), 146401.

S16. Yanai, T.; Tew, D. P.; Handy, N. C., A new hybrid exchange–correlation functional using the Coulomb-attenuating method (CAM-B3LYP). *Chem. Phys. Lett.* **2004,** *393* (1-3), 51-57.

S17. Brown, A. M.; McCusker, C. E.; McCusker, J. K., Spectroelectrochemical identification of charge-transfer excited states in transition metal-based polypyridyl complexes. *Dalton Trans.* **2014,** *43* (47), 17635-17646.

S18. Beckwith, J. S.; A., R. C.; and Vauthey, E., Data analysis in transient electronic spectroscopy – an experimentalist's view. *Int. Rev. Phys. Chem.* **2020,** *39* (2), 135-216.

S19. Fernández-Terán, R. J.; Sucre-Rosales, E.; Echevarria, L.; Hernández, F. E., A Sweet Introduction to the Mathematical Analysis of Time-Resolved Spectra and Complex Kinetic Mechanisms: The Chameleon Reaction Revisited. *J. Chem. Educ.* **2022,** *99* (6), 2327-2337.

S20. van Stokkum, I. H. M.; Larsen, D. S.; van Grondelle, R., Global and target analysis of time-resolved spectra. *Biochim. Biophys. Acta – Bioenerg.* **2004,** *1657* (2), 82-104.
